# AHSA1 Promotes Proliferation and EMT by Regulating ERK/CALD1 Axis in Hepatocellular Carcinoma

**DOI:** 10.3390/cancers14194600

**Published:** 2022-09-22

**Authors:** Jiakang Zhang, Zhixuan Ren, Dayong Zheng, Zhenghui Song, Junhao Lin, Yue Luo, Xiaopei Zou, Yingying Pan, Na Qi, Aimin Li, Xinhui Liu

**Affiliations:** 1 Cancer Center, Integrated Hospital of Traditional Chinese Medicine, Southern Medical University, Guangzhou 510315, China; 2 Cancer Center, Southern Medical University, Guangzhou 510515, China; 3 Research Center of Carcinogenesis and Targeted Therapy, Xiangya Hospital, Central South University, Changsha 410008, China; 4 Department of Pharmacy, Guilin Medical University, Guilin 541004, China

**Keywords:** Hepatocellular carcinoma, AHSA1, ERK1/2, CALD1

## Abstract

**Simple Summary:**

Globally, hepatocellular carcinoma is one of the leading causes of cancer-related death. Activator of HSP90 ATPase activity 1(AHSA1) was reported to be involved in regulating the maturation, stability, and degradation of related cancer-promoting proteins. However, the biological function and regulatory mechanism of AHSA1 in hepatocellular carcinoma were unclearly. In this study, we found AHSA1 was upregulated and associated with poor clinical characteristics and prognosis of hepatocellular carcinoma patients. Moreover, AHSA1 promoted proliferation, metastasis, epithelium-mesenchymal transition of hepatocellular carcinoma both in vitro and in vivo by recruiting ERK1/2 and promoting the phosphorylation and inactivation of CALD1. Taken together, our findings provided a mechanistic insight into the role of AHSA1 in hepatocellular carcinoma progression and implied AHSA1 may be a biomarker and therapeutic target for hepatocellular carcinoma patients.

**Abstract:**

Hepatocellular carcinoma (HCC) is one of the major causes of cancer-related death worldwide. AHSA1 as a chaperone of HSP90 promotes the maturation, stability, and degradation of related cancer-promoting proteins. However, the regulatory mechanism and biological function of AHSA1 in HCC are largely unknown. Actually, we found that AHSA1 was significantly upregulated in HCC tissues and cell lines and was notably correlated with the poor clinical characteristics and prognosis of HCC patients in this study. Furthermore, both in vitro and in vivo, gain- and loss-of-function studies demonstrated that AHSA1 promoted the proliferation, invasion, metastasis, and epithelial-mesenchymal transition (EMT) of HCC. Moreover, the mechanistic study indicated that AHSA1 recruited ERK1/2 and promoted the phosphorylation and inactivation of CALD1, while ERK1/2 phosphorylation inhibitor SCH772984 reversed the role of AHSA1 in the proliferation and EMT of HCC. Furthermore, we demonstrated that the knockdown of CALD1 reversed the inhibition of proliferation and EMT by knocking AHSA1 in HCC. We also illustrated a new molecular mechanism associated with AHSA1 in HCC that was independent of HSP90 and MEK1/2. In summary, AHSA1 may play an oncogenic role in HCC by regulating ERK/CALD1 axis and may serve as a novel therapeutic target for HCC.

## 1. Introduction

Primary liver cancer is one of the primary causes of cancer-related death worldwide. Hepatocellular carcinoma (HCC) is the most common type of primary liver cancer, risk factors of which include hepatitis B virus (HBV) infection, hepatitis C virus (HCV) infection, alcohol abuse, and so on [1]. Since most HCC patients were diagnosed in advanced stage and therapeutic strategies are limited, the 5-year survival rate of HCC is still under 20% [2]. Targeted therapy, as a vital method for advanced HCC patients, includes lenvatinib and sorafenib [3,4,5]. However, the clinical efficacy of current therapies is still not satisfactory for HCC patients. Thus, it remains critical to understand the molecular mechanism and identify therapeutic targets for HCC.

The activator of 90 kDa heat shock protein ATPase homolog 1 (AHSA1) was reported to play a role in the regulation of related cancer-promoting proteins [6,7,8,9] depending on a chain reaction between the central region of HSP90 and the AHSA1 N-terminal domain [10]. Recently, AHSA1 has been found to play an oncogenic role by promoting the proliferation and metastasis of malignant tumors, including osteosarcoma, colorectal adenocarcinoma, and multiple myeloma [11,12,13]. However, the functional role and underlying mechanism of AHSA1 in the occurrence and development of HCC have not yet been characterized.

In this study, we systematically evaluated the expression pattern of AHSA1 in the HCC public database and tissue, and we identified that AHSA1 was significantly unregulated and positively correlated with poor clinical characteristics and prognosis in HCC patients. Furthermore, we demonstrated that AHSA1 promoted the proliferation, invasion, metastasis, and epithelial-mesenchymal transition (EMT) of HCC both in vitro and in vivo. Moreover, the mechanistic study indicated that AHSA1 recruited ERK1/2 and promoted the phosphorylation and inactivation of CALD1, while ERK1/2 phosphorylation inhibitor SCH772984 reversed the role of AHSA1 in the proliferation and EMT of HCC. Knockdown of CALD1 reversed the inhibition of proliferation and EMT by knocking AHSA1 in HCC. The findings in this study reveal a novel carcinogenic role of AHSA1 in HCC and provides a new direction for HCC treatment.

## 2. Materials and Methods

### 2.1. Bioinformatics Analysis

Data required for bioinformatics analysis were downloaded from the GEO (GSE14520 and GSE50579) and TCGA-LIHC databases. R software (version 3.5.1) and its packages were employed to analyze these data. The median expression value of AHSA1 was used to divide the HCC patients into AHSA1 high-expression and AHSA1 low-expression groups from the TCGA-LIHC database. The available clinical information from the TCGA-LIHC database were displayed in Table 1. AHSA1-related genes (|R > 0.5|, *p* < 0.001) in TCGA-LIHC were identified and used for functional and pathway enrichment analyses, including the Kyoto Encyclopedia of Genes and Genomes (KEGG) pathway and gene ontology (GO). We used a publicly available online tool to perform survival analysis to show the relationship between AHSA1 expression and HCC patients’ overall survival (OS) and disease-free survival (DFS) (http://gepia.cancer-pku.cn/index.html, accessed on 12 September 2022).

### 2.2. Antibodies

AHSA1, N-cadherin, 6-his, vimentin, HSP90, MEK1/2, E-cadherin, and CALD1 antibodies were obtained from Proteintech (Wuhan, China). ERK1/2, Phospho-MEK1/2, and Phospho-ERK1/2 antibodies were obtained from Cell Signaling Technology (Beverly, MA, USA). Ki67 antibody was obtained from Abcam (Cambridge, MA, USA). Phospho-CALD1 antibody was purchased from Thermo Fisher Scientific (Waltham, MA, USA). β-tubulin antibody was obtained from Bioworld Technology (Bloomington, MN, USA).

### 2.3. Cell Culture and Transfection

The HCC cell lines PLC/PRF/5, HCCLM3, Huh-7, Hep3B, and HepG2, and the control liver epithelial cell line LO2 were purchased from Zhong Qiao Xin Zhou Biotechnology (Shanghai, China). The cells were inoculated into culture dishes purchased from Guangzhou Jet Biofiltration (Guangzhou, China) and added to Dulbecco’s Modified Eagle’s Medium (DMEM) containing 10% fetal bovine serum to maintain growth. The growth environment temperature was maintained at 37 °C with 5% CO_2_. The overexpression plasmid pcDNA3.1-AHSA1, empty vector pcDNA3.1, shRNAs targeting AHSA1, and sh-control were purchased from OBiO Technology (Shanghai, China). The sequences of shRNAs targeting AHSA1 and sh-control were as follows: sh-AHSA1-1, GCATGATCTTACCTACAAT; sh-AHSA1-2, CCATCACCTTGACCTTCAT; sh-control, CCTAAGGTTAAGTCGCCCTCG. The siRNAs targeting CALD1 and si-NC were obtained from RIBOBIO (Guangzhou, China). The sequences of siRNAs targeting CALD1 were as follows: si-CALD1-1, AGAGCTTCATGGATCGAAA; si-CALD1-2, GTACGCAACATCAAGA GTA; si-CALD1-3, GAAGGAGTTCGACCCAACA. Lipofectamine 3000 (Thermo Scientific, Waltham, MA, USA) was used for conventional cell transfection for 72 h. AHSA1 expression was detected by western blotting.

### 2.4. Immunohistochemistry (IHC)

Referring to the manufacturer’s instructions, the IHC detection kit (PV-9000) (ZsBio, Beijing, China), was used to assess the expression of AHSA1 in the HCC tissue arrays (HLivH180Su15) (Shanghai Outdo Biotech Company, Shanghai, China). The IHC score was obtained by determining the intensity of staining and the positive area (intensity score and area score). The intensity of staining was scored 0–3 (0 = no staining, 1 = weak staining, 2 = moderate staining and 3 = strong staining). The positive area of staining was scored 0–4 (0 < 5%, 1 = 5–25%, 2 = 26–50%, 3 = 51–75%, and 4 > 75%). The IHC score was calculated by multiplication of the two scores and described by the following rules: scores between 0 and 6 represented low expression, and those >6 were considered high expression.

### 2.5. Total RNA Extraction and Real-Time PCR

By means of the total RNA isolation kit (Foregene, Chengdu, China) and the PrimeScript RT kit (Takara Biomedical Technology, Beijing, China), RNA was extracted from the cells and reverse transcribed into DNA. Referring to the operation manual of the LightCycler480 system (Roche, Basel, Switzerland), DNA was added into the 8-tube system containing primers and TB Green Premix Ex TaqII (Takara Biomedical Technology, Beijing, China) to conduct Real-Time PCR. DNA expression was standardized to the expression of β-actin, and the relative expression level was calculated using 2^–ΔΔCt^. The primer sequences included: AHSA1: (forward) 5′-GGCACTA AGCGGTCCTGAG-3′, (reverse) 5′-CTCCACTTCATCCACGCTGT-3′; β-actin: (forward) 5′-AGAAGGATTCCTATGG GCGAC-3′, and (reverse) 5′–AGTACTTGCGCTCA GGAGGA-3′.

### 2.6. Western Blotting, Coimmunoprecipitation (Co-IP) Assay, and Mass Spectrometer (MS) Assay

In a brief, lysis mixture (including phosphatase inhibitor and protease inhibitor) was added to cells at 4 °C for 1 h. Next, the cells were crushed with an ultrasonic instrument 3 times according to the instructions of the instrument. The samples were centrifuged at 12,000× *g* rpm for 15 min. Protein supernatant was retained and was added to Protein Sample Loading Buffer at 100 °C for 10 min. Protein samples were separated on 10% pre-gel and transferred to a polyvinylidene fluoride (PVDF) membrane. Using 5% BSA at 37 °C for 1 h, the bands were blocked and incubated with a primary antibody at 37 °C for 2 h. After 3 cleanings using TBST, the band was incubated with a secondary antibody on a shaker at 37 °C for 1.5 h. Via the MiniChemi chemiluminescence imaging and analysis system (Sage Creation Science, Beijing, China), the special protein bands of the membrane became visible on the computer screen after spraying enhanced chemiluminescence reagents from Millipore (Billerica, MA, USA). HCC cell protein samples were coimmunoprecipitated using the Pierce Co-IP kit (Thermo Scientific, Waltham, MA, USA), the target protein that corresponded to the cured antibody and its stable binding protein were obtained, and the final special protein samples were analyzed by Western blotting. For the MS assay, the protein sample obtained from the Co-IP experiment contained proteins interacting with AHSA1, and 8 M of urea was used to supplement the volume of the protein sample to 200 uL. Next, the sample was added with a final concentration of 2 mM of DTT, reacted in an incubator at 56 °C for 30 min, and cooled to room temperature. The sample was added with a final concentration of 10 mM of IAA and reacted at room temperature for 30 min. Then, the sample was added to a 10 KD protein ultrafiltration tube and centrifuged (14,000× *g*, 30 min), washed three times using ammonium bicarbonate solution, and then digested by trypsin (37 °C, 12 h). Finally, the sample was washed with water for mass spectrometry, centrifuged (14,000× *g*, 20 min), dried, and analyzed by one mass spectrometer (Thermo Scientific^TM^ Orbitrap Fusion^TM^ Tribrid^TM^, American). The original data was compared with the data in the UniProt database.

### 2.7. EdU Assay

An EdU proliferation test was conducted using the in vitro Cell-Light EdU Apollo 567 imaging kit (RiboBio, Guangzhou, China). In brief, HCC cells were transfected with a plasmid or transduced with lentivirus and cultured for 48 h before being seeded in 96-well plates (2 × 10^4^ cells/well) and cultured for another 24 h before EdU according to the manufacturer’s instructions. The figures of EdU-positive cells (red) in three random observation fields were captured via an inverted fluorescence microscope (Olympus, Beijing, China).

### 2.8. Clone Formation Experiment

Cell population dependence and proliferation were evaluated using a cell cloning experiment. In brief, HepG2 and HCCLM3 cells, receiving transduction of AHSA1 knockdown lentivirus or control lentivirus, were evenly distributed at the bottom of 6-well plates (500 cells/well) after the cells were incubated for three weeks. The cells were then infiltrated with paraformaldehyde for 60 min and stained with crystal violet (0.1% methanol) for 20 min. Finally, the number of clones was calculated.

### 2.9. Cell Counting Kit 8 (CCK-8) Assay

Hep3B cells were evenly inoculated in 96-well plates (2000 cells/well), and the corresponding SCH772984 (Selleck Chemicals, Shanghai, China) was added via the concentration gradient. CCK-8 (Dojindo Laboratories, Mashikimachi, Japan) was used two hours before, and the absorbance of the sample in the hole at 450 nm was measured at 24, 48, and 72 h.

### 2.10. Cell Wound Healing, Migration, and Invasion Assays

To conduct the cell wound healing test, cells were added to a six-well plate, the tip of a 1-mL pipette was used to make scratches, and cell migration across the gaps was observed via a microscope at 0, 24, 48, and 72 h. For the Transwell experiment, the suspended cells in DMEM without serum were inoculated in the chamber (the aperture of the basement membrane was 8.0 mm, 1 × 10^5^ cells/chamber) and placed in a 24-well plate. The corresponding wells were supplemented with DMEM containing 20% serum and incubated for 24 h. Cells were removed which did not enter the basement membrane, and the membrane was placed in paraformaldehyde for cell immobilization. The membrane was then dyed with crystal violet solution (0.1% methanol) for 20 min. Positive cells on the membrane (purple) were counted using three fields of vision. For the invasion experiment, a matrix gel (Corning, Shanghai, China) was diluted referring to the manufacturer’s instructions, and evenly spread on the surface of the membrane. Cells suspended in DMEM without serum were inoculated in the chamber (the aperture of the basement membrane was 8.0 mm, 1 × 10^5^ cells/chamber) and placed in a 24-well plate. DMEM with 20% serum was supplemented to the corresponding well of each chamber. After 24 h, cells were removed which did not enter the basement membrane, and the membrane was placed in paraformaldehyde for cell immobilization. The membrane was then dyed in crystal violet solution (0.1% methanol) for 20 min. Positive cells on the membrane (purple) were counted using three fields of vision.

### 2.11. Subcutaneous Model of Nude Mice

According to the international animal care and maintenance regulations, the mouse experiment was conducted and was approved by the Experimental Animal Ethics Committee, Guilin Medical University. In short, 2.5 × 10^6^ HCCLM3 cells with knocked-down AHSA1 or control lentivirus were used to inject into the posterior right lateral thigh of nude mice (male, bodyweight: ~19 g, 6 weeks) (Hunan SJA laboratory animals, Hunan, China). The mice were euthanized on day 17 to evaluate tumor size. All tumor tissues were observed and photographed through a microscope (Olympus, Beijing, China). Each tumor tissue specimen was then fixed with formalin, embedded in paraffin, and H&E and HRP-DAB immunohistochemical staining were performed.

### 2.12. Nude Mouse Lung Metastasis Model 

In brief, 1 × 10^6^ cells/80 μL AHSA1 knockdown or empty vector control HCCLM3 cells were intravenously injected into the tail vein of nude mice. Lung metastasis model mice were euthanized on day 58 to evaluate the number of tumors. All lung tissues were observed and photographed through a microscope (Olympus, Beijing, China). Each lung tissue specimen was fixed with formalin, embedded in paraffin, and H&E and HRP-DAB immunohistochemical staining were performed.

### 2.13. Statistical Analysis

Student T-tests were used for analysis. The relationship between AHSA1 expression and patient clinical and prognostic characteristics was analyzed by χ^2^ or Fisher’s exact tests. OS and DFS were calculated by log-rank test. All statistical results are expressed as the mean ± standard error by GraphPad Prism6 (San Diego, CA, USA) and met the conditions of a bilateral test. Each non-animal experiment was repeated in three independent experiments.

## 3. Results

### 3.1. AHSA1 Was Upregulated and Predicted a Poor Prognosis of HCC

**Figure 1:**
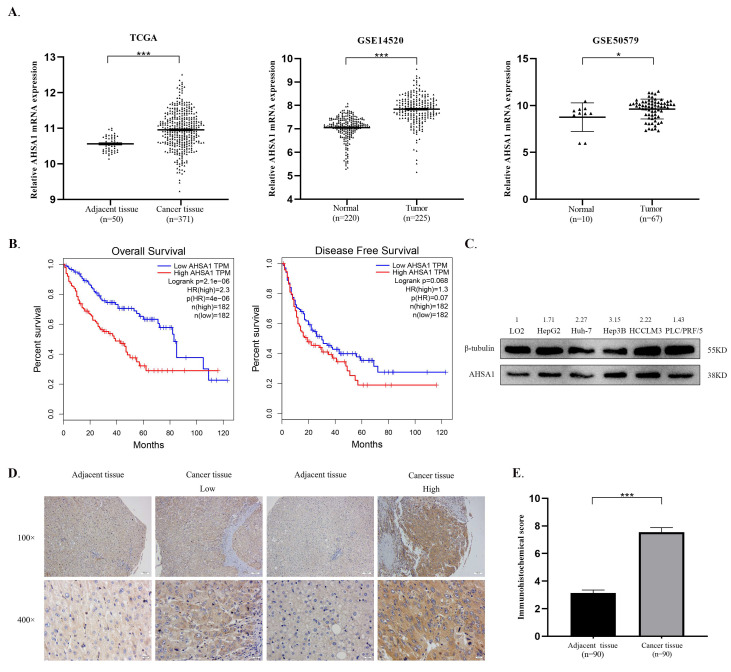
AHSA1 was upregulated and predicted a poor prognosis of HCC. (**A**). AHSA1 was significantly upregulated in HCC cancerous tissues compared with adjacent normal liver tissues in the indicated HCC database. (**B**). HCC patients with high expression of AHSA1 had shorter OS and DFS than those with low expression of AHSA1in TCGA-LIHC database. (**C**). Protein expression of AHSA1 in five common HCC cells and one normal immortalized hepatic epithelial cell (LO2). (**D**,**E**). Representative IHC images and IHC scores of AHSA1 expression in HCC cancer and adjacent tissues of 90 HCC patients. *, *p* < 0.05; ***, *p* < 0.001. The uncropped blots are shown in Supplementary Materials.

To explore the role of AHSA1 in HCC development, we first detected the expression of AHSA1 in the TCGA-LIHC, GSE14520, and GSE50579 HCC databases [14]. As shown in Figure 1A, AHSA1 was significantly upregulated in HCC cancerous tissues compared to adjacent tissues. Moreover, we found that the expression of AHSA1 was positively correlated with TNM stage (*p* < 0.01, Figure S1A) and pathological grade (*p* < 0.05, Figure S1B) of HCC patients in TCGA-LIHC database. The top five genes with the most frequent mutations were TERT, TP53, CTNNB1, AXIN1, and ARID1A in the TCGA-LIHC database. Furthermore, we detected the association of AHSA1 expression with these five mutation genes in HCC patients. Interestingly, the expression of AHSA1 was significantly associated with the mutation of TP53, CTNNB1 and ARID1A in HCC (Figure S1C–G). Next, we performed survival analysis of AHSA1 in the TCGA-LIHC dataset and found that HCC patients with high expression of AHSA1 had shorter OS and DFS than those with low expression of AHSA1 (Figure 1B). Moreover, we performed the univariate and multivariate Cox regression analysis of AHSA1 and clinical characteristics in TCGA-LIHC database (Table 2). We found that expression of AHSA1 as well as tumor stage are the independent prognosis predictors for HCC patients. Furthermore, the basic expression levels of AHSA1 in HCC cell lines were higher than those in the normal cell line LO2, which were detected by quantitative real-time PCR and Western blot (Figure 1C and Figure S2A). To confirm the protein level of AHSA1 in HCC, IHC staining was employed in the HCC microarray, including 90 HCC paired tissues. Consistently, AHSA1 expression was significantly higher in HCC tissues than in adjacent tissues (*p* < 0.001, Figure 1D,E). Subsequently, the potential association between AHSA1 and HCC patients’ clinicopathological features was explored to understand the significance of AHSA1 in HCC. Notably, AHSA1 expression was positively correlated with HCC recurrence (*p* < 0.001, Table 3). 

### 3.2. AHSA1 Promoted the Proliferation of HCC Both In Vitro and In Vivo

**Figure 2:**
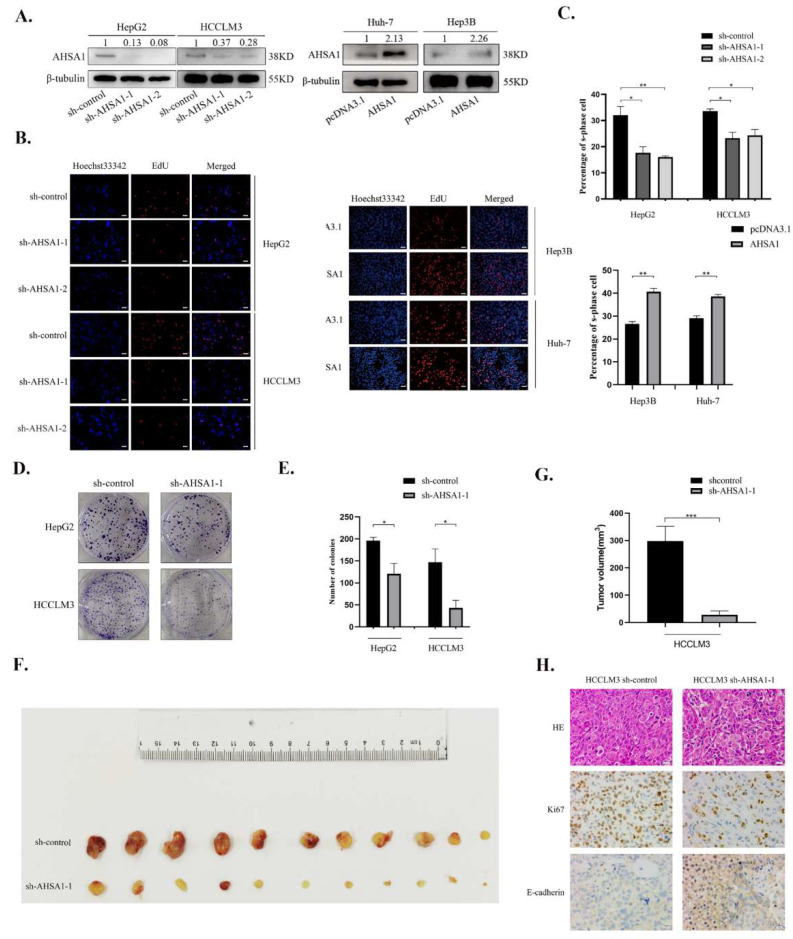
AHSA1 promoted the proliferation of HCC both in vitro and in vivo. (**A**). Transfection efficiency was determined by Western blotting in indicated HCC cell lines. (**B**,**C**). EdU detection and corresponding statistical analysis were performed on HCC cells. (**D**,**E**). Cell cloning and statistical analysis were performed on HCC cells after knocking down AHSA1. (**F**,**G**). Subcutaneous tumor and statistical analysis of tumor volume of HCCLM3-con and HCCLM3-shAHSA1 cells in nude mice. (**H**). HE and IHC staining of Ki-67 and E-cadherin in subcutaneous tumor tissue. *, *p* < 0.05; **, *p* < 0.01; ***, *p* < 0.001. The uncropped blots are shown in Supplementary Materials.

To assess the role of AHSA1 in HCC, AHSA1 was knocked down in HepG2 and HCCLM3 cells using lentiviral transduction and overexpressed in Huh-7 and Hep3B cells. Transfection efficiency was determined by Western blotting (Figure 2A). EdU analysis showed that knockdown of AHSA1 significantly inhibited cell proliferation ability in HepG2 and HCCLM3 cells compared to control cells. Moreover, the cell proliferation ability of Hep3B and Huh-7 cells were significantly upregulated by overexpression of AHSA1 (Figure 2B,C). Consistently, the results of the clone formation experiment indicated that proliferation was obviously reduced in HepG2 and HCCLM3 cells after being transfected with sh-AHSA1-1 compared to the control group (*p* < 0.05, Figure 2D,E). 

A nude mouse subcutaneous tumor model was used to further analyze the effect of AHSA1 on the proliferation of HCC in vivo. HCCLM3 cells transfected with AHSA1 knockdown lentivirus or control lentivirus were injected into the skin of the right posterolateral thigh of male nude mice. As shown in Figure 2F,G and Figure S2B, a significant reduction of the tumor was found in mice injected with HCCLM3-sh-AHSA1-1, compared with those injected with HCCLM3-sh-control cells (*p* < 0.001). Furthermore, a reduction of Ki67 expression was observed in the HCCLM3-sh-AHSA1-1 group than HCCLM3-sh-control group by employing IHC staining (Figure 2H). Interestingly, the expression of E-cadherin expression was upregulated in the tumor of the HCCLM3-sh-AHSA1-1 group. Overall, AHSA1 upregulated the proliferation ability of HCC both in vitro and in vivo.

### 3.3. AHSA1 Promoted the Invasion, Migration, and EMT of HCC Both In Vitro and In Vivo

**Figure 3:**
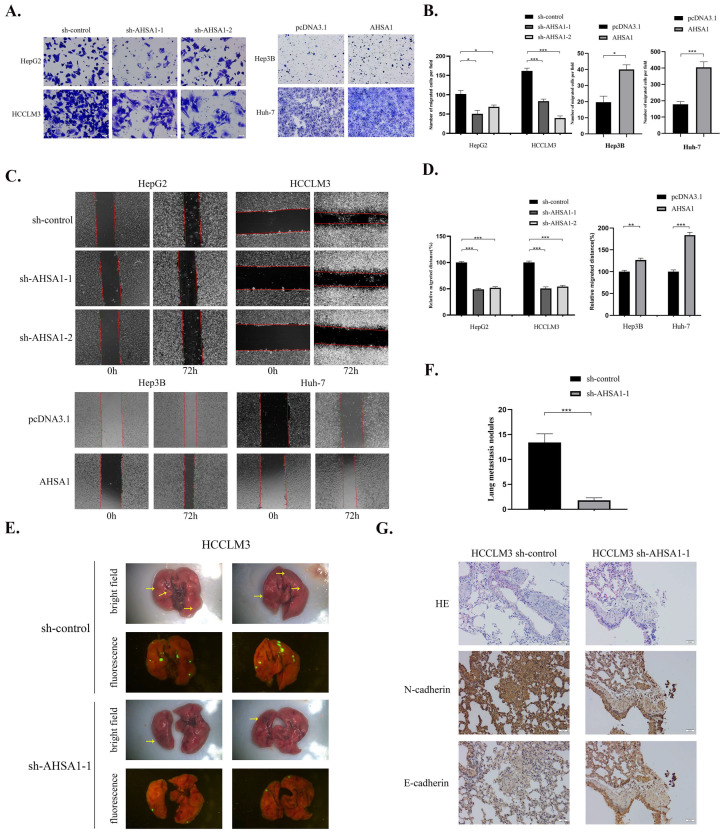
AHSA1 promoted the invasion, migration, and EMT of HCC both in vitro and in vivo (**A**,**B**). Representative images and quantitative analysis of Transwell assays in indicated HCC cell lines. (**C**,**D**). Representative images and the corresponding quantitative analysis of wound healing assays. (**E**,**F**). Representative images and quantitative analysis of the number of lung metastatic nodules in a nude mouse lung metastasis model by tail vein injection of indicated HCC cells; yellow arrow represents metastasis. (**G**). Representative images of HE and IHC staining of N-cadherin and E-cadherin in lung tissue of nude mice. *, *p* < 0.05; **, *p* < 0.01; ***, *p* < 0.001.

Previous studies have shown that high AHSA1 expression is related to the occurrence of metastases [15], and the E-cadherin expression was upregulated by knockdown AHSA1 in vivo. We further explore the influence of AHSA1 on HCC cell migration, and invasion by employing Transwell invasion, migration, and wound healing assays. The results showed that cell invasion and migration were significantly inhibited in the sh-AHSA1-1 group compared with the control group in both HepG2 and HCCLM3 cells, while overexpression of AHSA1 significantly promotes Hep3B and Huh-7 cells invasion and migration (Figure 3A,B and Figure S2C,D). Consistently, a shorter healing distance was detected in the sh-AHSA1-1 group compared with the control group, while reinforcement of cell healing was observed following AHSA1 overexpression (Figure 3C,D). These results indicated that AHSA1 has a positive effect on cell invasion and migration of HCC.

To ascertain whether AHSA1 knockdown affects tumor metastasis in vivo, HCCLM3 cells transfected with AHSA1 knockdown lentivirus or control lentivirus were intravenously injected into 6-week-old male nude mice via tail vein. All mice were euthanized after 58 days to analyze the effect of AHSA1 on tumor cell migration. As shown in Figure 3E,F, there were significantly fewer fluorescent tumor nodules in the lungs of mice injected with HCCLM3-sh-AHSA1-1, compared with those injected with HCCLM3-sh-control cells (*p* <0.001). In addition, N-cadherin was downregulated and E-cadherin was upregulated in tumor tissues of mice injected with HCCLM3-sh-AHSA1-1, suggesting that loss of AHSA1 reduced the EMT of HCC in vivo (Figure 3G). Thus, AHSA1 promoted cell migration and invasion ability of HCC in vitro and in vivo.

### 3.4. AHSA1 Promoted the Phosphorylation and Inactivation of CALD1 by Phosphorylation of ERK1/2

**Figure 4:**
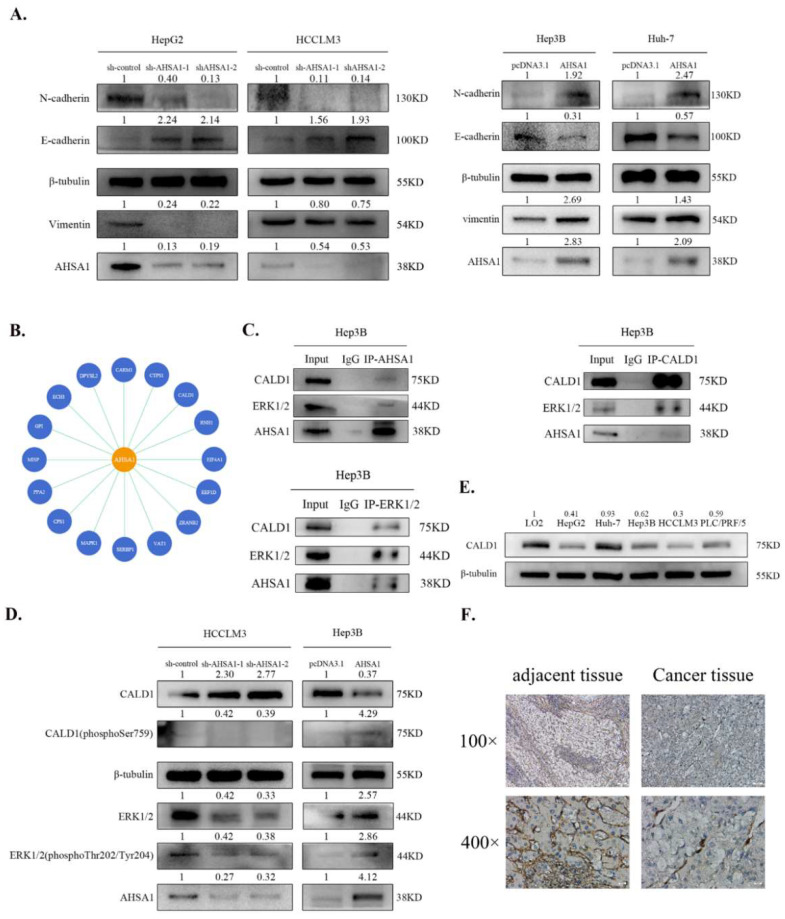
AHSA1 promoted the phosphorylation and inactivation of CALD1 by phosphorylation of ERK1/2 (**A**). EMT markers were analyzed by Western blot by changing AHSA1 expression in HCC cells. (**B**). Proteins that may interact with AHSA1 were obtained by qualitative mass spectrometry. (**C**). AHSA1-ERK1/2-CALD1 was analyzed using Co-IP of the Hep3B cell lysate and Western blot. (**D**). Protein levels of downstream molecules in HCC cells with AHSA1 knockdown or overexpression were analyzed by Western blot. (**E**). CALD1 protein expression in HCC cells lines and normal immortalized liver epithelial cells were analyzed. (**F**). Representative IHC images of CALD1 expression in cancer and adjacent tissues from HCC patients. The uncropped blots are shown in Supplementary Materials.

Additional experiments were determined to explore the molecular mechanism of AHSA1 in HCC. Notably, overexpression of AHSA1 promoted the EMT of Hep3B and Huh-7 by upregulating the mesenchymal markers N-cadherin and vimentin and downregulating the epithelial marker E-cadherin, while knockdown of AHSA1 had an opposite effect on HepG2 and HCCLM3 cells (Figure 4A). In addition, AHSA1-related genes detected in the TCGA-LIHC database were employed in KEGG and GO enrichment analysis. Through bioinformatic analysis, we found that AHSA1-related genes were predominantly enriched in cell cycle, spliceosome, and DNA replication (Figure S2E). Moreover, the Co-IP protein sample solution was pulled down by AHSA1 antibody through protein Co-IP and assessed by qualitative mass spectrometry to determine the interaction protein of AHSA1 in HCC. As shown in Figure 4B, sixteen proteins were strongly correlated with AHSA1, of which MAPK1 (ERK1/2) and its downstream target CALD1 were particularly noteworthy [16,17]. Next, Co-IP was used to verify the interaction between AHSA1, CALD1, and ERK1/2 in Hep3B and Huh-7 cells (Figure 4C and Figure S3A). Furthermore, knockdown of AHSA1 promoted the protein level of CALD1 in HCCLM3, while overexpression of AHSA1 had a reverse effect on Hep3B cells (Figure 4D). Prior studies have reported that phosphorylated ERK1/2 promoted CALD1 phosphorylation and inactivation. As shown in Figure 4D, knockdown of AHSA1 inhibited CALD1 phosphorylation at Ser759 and ERK1/2 phosphorylation at Thr202/Tyr204, while overexpression of AHSA1 had a reverse effect on Hep3B cells. Taken together, these results suggested that AHSA1 might promote phosphorylation of CALD1 at Ser759 and reduce CALD1 activity (in its non-phosphorylated form) by inducing phosphorylation of ERK1/2 at Thr202/Tyr204. In addition, we detected that CALD1 expression was significantly lower in HCC tissues and cell lines (Figure 4E,F, and Figure S3B). Interestingly, the molecular mechanism associated with AHSA1 in HCC cells was independent of HSP90 and MEK1/2 (Figure S3C,D).

### 3.5. ERK1/2 Phosphorylation Inhibitor Reversed the Proliferation and EMT of HCC That was Promoted by AHSA1 Overexpression

**Figure 5:**
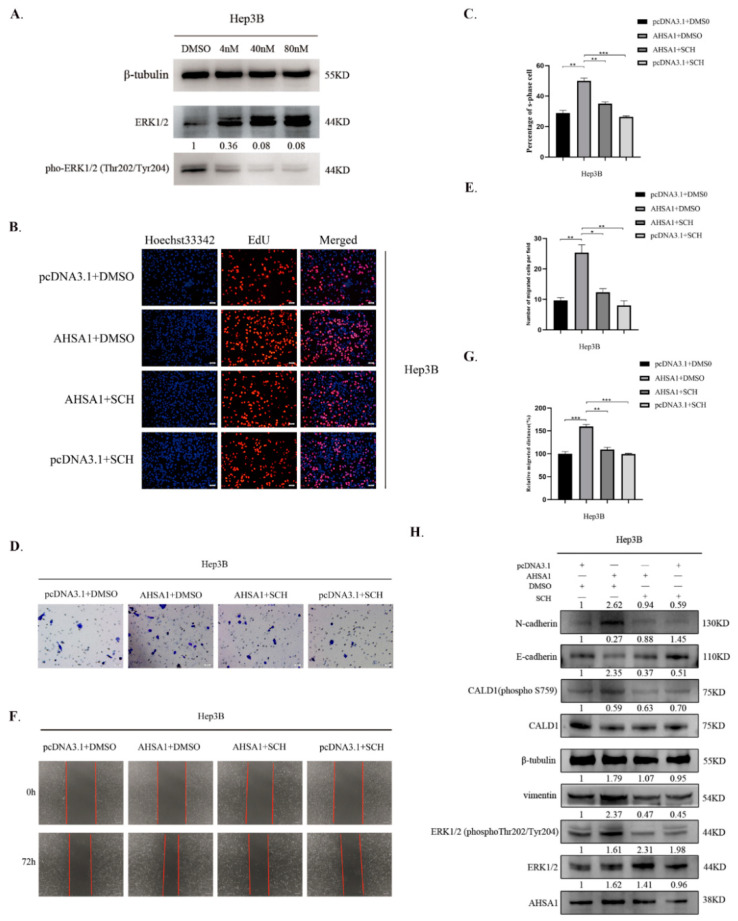
Inhibition of ERK1/2 phosphorylation reversed the HCC cell proliferation and EMT that was promoted by AHSA1 overexpression (**A**). The inhibitory efficiency of different concentrations of SCH772984 on Hep3B cells was analyzed by Western blot. (**B**,**C**). Representative images and quantitative analysis of EdU staining showed that SCH772984 reversed the proliferation induced by overexpression of AHSA1 in Hep3B cells. (**D**–**G**). Representative images and quantitative analysis of Transwell and wound healing assays showed that SCH772984 reversed the migration induced by overexpression of AHSA1 in Hep3B cells. (**H**). The changes in protein expression after inhibiting ERK1/2 phosphorylation in Hep3B cells that overexpressed AHSA1 were detected by Western blot. *, *p* < 0.05; **, *p* < 0.01; ***, *p* < 0.001. The uncropped blots are shown in Supplementary Materials.

To verify the effect of ERK1/2 on the promotion of proliferation and EMT by AHSA1 in HCC, rescue experiments were performed with an ERK1/2 phosphorylation inhibitor, SCH772984. Prior studies set forth that SCH772984 inhibits the phosphorylation of residues in the ERK self-activation ring [18]. Within a certain range, CCK-8 results showed that as the concentration of SCH772984 increased, its effect on the survival of Hep3B cells was negligible (Figure S3E). Based on the drug manufacturer’s instructions and related literature reports [18], 4, 40, and 80 nM doses of SCH772984 were used, and as expected, ERK1/2 phosphorylation decreased after SCH772984 treatment (Figure 5A). 40 nmol/l (nM) doses of SCH772984d as the effective concentration were used for further rescue assays. Using EdU assay, we found that overexpression of AHSA1 significantly promoted HCC proliferation, which could be reversed by SCH772984 (Figure 5B,C). Subsequent Transwell and wound healing test assays revealed that overexpression of AHSA1 led to an increase in HCC migration, which could be reversed by SCH772984 (Figure 5D–G). In addition, the EMT and CALD1 regulated by AHSA1 overexpression were also reversed after treatment with SCH772984 (Figure 5H). Taken together, SCH772984, as the ERK1/2 phosphorylation inhibitor, abrogated AHSA1-induced HCC proliferation and migration.

### 3.6. Inhibition of CALD1 Reversed the Inhibition of Cell Proliferation and EMT in HCC by Knockdown of AHSA1

**Figure 6:**
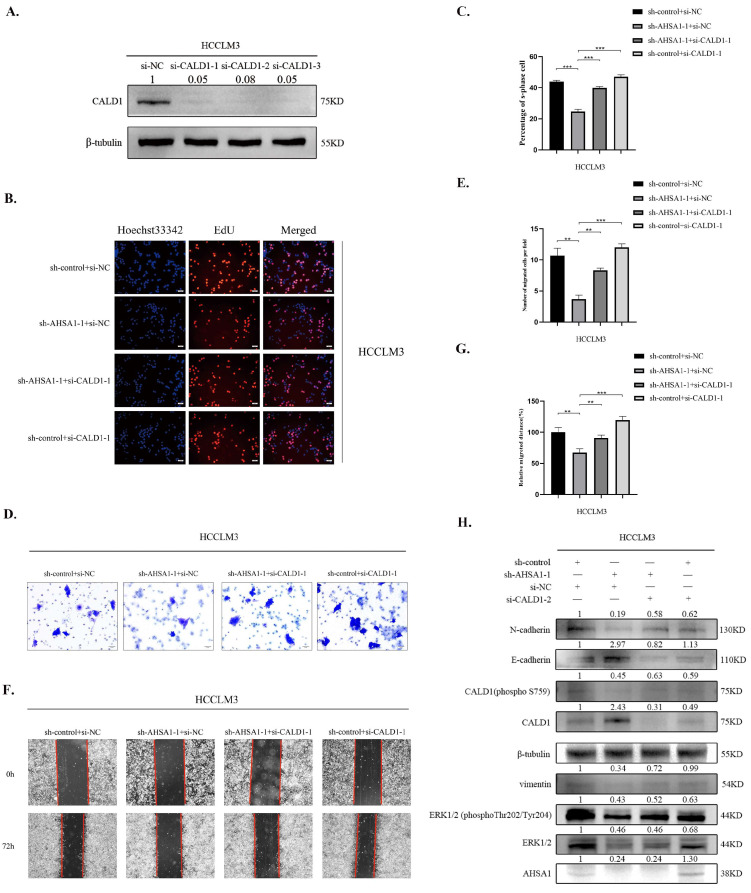
Inhibition of CALD1 expression reversed the inhibition of proliferation and EMT in HCC by knockdown of AHSA1 (**A**). The efficiency of CALD1 knockdown in HCCLM3 cells was detected by Western blot. (**B**,**C**). Representative images and quantitative analysis of EdU imaging showed that knockdown of AHSA1 significantly inhibited HCC proliferation, which could be reversed by CALD1 silencing. (**D**–**G**). Representative images and quantitative analysis of Transwell and wound healing assays showed knockdown of AHSA1 led to decreased HCC invasion and migration, which could be reversed by CALD1 silencing. (**H**). The changes in protein expression after inhibiting CALD1 expression in HCCLM3 cells with AHSA1 knockdown were detected by Western blot. **, *p* < 0.01; ***, *p* < 0.001. The uncropped blots are shown in Supplementary Materials.

To verify whether CALD1 may affect the oncogene role of AHSA1 in HCC, functional rescue experiments were performed. Inhibition of CALD1 expression by siRNA was then used, and evidence of the knockdown efficiency of CALD1in HCCLM3 cells was offered by Western blot analysis (Figure 6A). Using EdU assay, we found that knockdown of AHSA1 significantly inhibited HCC proliferation which could be reversed by CALD1 silencing(Figure 6B,C). Subsequent Transwell and wound healing tests assays revealed that knockdown of AHSA1 led to decreased of HCC migration, which could be reversed by CALD1 silencing (Figure 6D–G). Moreover, the EMT and CALD1 regulated by AHSA1 overexpression were also reversed after the knockdown of CALD1 (Figure 6H). Therefore, we conclude that CALD1 is required for AHSA1-induced HCC proliferation and EMT.

## 4. Discussion

Many HSP90 downstream proteins and molecular chaperones play a key role in cancer pathogenesis, suggesting that HSP90 is critical for an effective response to cancer treatment [19]. Actually, clinical trials by means of some HSP90 inhibitors have stopped as a result of serious side effects such as ototoxicity and hepatotoxicity or stalled due to therapy effects lower than expected [20,21]. HSP90, an important molecule for cellular processes, was suppressed with severe consequences, emphasizing the value of further expounding the molecular chaperone mechanism of HSP90. AHSA1, one of the most active chaperones of HSP90, stimulated HSP90 ATPase activity to stabilize and strengthen the function of downstream target proteins [22]. However, the mechanism of AHSA1 in HCC has remained largely unexplored. This study correlated AHSA1 overexpression with the mutation of TP53, CTNNB1, ARID1A, and poor clinical characteristics and prognosis in HCC patients and demonstrated that AHSA1 promoted the proliferation, invasion, metastasis, and EMT of HCC both in vitro and in vivo, and suggested that AHSA1 may be a carcinogenic gene in HCC.

EMT plays a critical role in proembryo formation, adult wound healing, and the development of many types of cancer. Indeed, tumor cells with EMT properties are important to cancer development [23]. This study showed that knocking down AHSA1 in two typical HCC cell lines, HepG2, and HCCLM3 resulted in downregulation of the mesenchymal markers, N-cadherin and vimentin, and the upregulation of the epithelial marker E-cadherin. When AHSA1 expression was increased after plasmid transfection in Hep3B and Huh-7 cells, the results of the loss of E-cadherin and upregulation of N-cadherin and vimentin were obtained. These findings indicated that AHSA1 could promote the epithelial to mesenchymal transformation in HCC.

Surprisingly, we demonstrated that AHSA1 recruited ERK1/2 and CALD1 and promoted ERK1/2 phosphorylation at Thr202/Tyr204, thereby increasing CALD1 phosphorylation at Ser759, causing CALD1 to lose its ability to inhibit ATPase activity and actin filament movement, ultimately increasing cell migration [24,25]. An initial rescue experiment showed that AHSA1-induced ERK1/2 phosphorylation, and subsequent EMT and HCC cell proliferation were prevented by the ERK1/2 phosphorylation inhibitor SCH772984. A second rescue experiment, using an siRNA-mediated gene knockdown technique to inhibit CALD1 expression, was designed to verify whether knocking down AHSA1 inhibited EMT and HCC cell proliferation by increasing the functional (non-phosphorylated) form of CALD1. In short, AHSA1 further targeted CALD1 and promoted its phosphorylation by increasing the phosphorylation of ERK1/2 and enhancing EMT and HCC cell proliferation. Actually, these results are supported by previous findings that CALD1 inhibits cancer cell metastasis [26,27]. Interestingly, the molecular mechanism of AHSA1 in HCC cells was independent of HSP90 and MEK1/2. Overall, this study showed that AHSA1, an important target for HCC treatment, is overexpressed in HCC patients and is associated with a poor prognosis. In fact, the oral ERK1/2 phosphorylation inhibitor MK-8353, which has a similar mechanism to the ERK1/2 phosphorylation inhibitor SCH772984, has shown some promising results in clinical studies of cancer patients [28], implies a new choice for patients with AHSA1 overexpression.

In summary, this study showed that AHSA1 increased phosphorylation of ERK1/2 and prevented CALD1 activity, ultimately enhancing EMT and HCC cell proliferation. AHSA1 may function as an effective diagnostic and prognostic biomarker for HCC patients. Additional clinical trials should be considered to assess patient sensitivity to MK-8353 when the AHSA1 of the patient is overexpressed.

## Figures and Tables

**Table 1:** Clinical characteristics in HCC patients with AHSA1-high expression or AHSA1-low expression in TCGA-LIHC database.

Clinical Characteristics	AHSA1-Low Group (*n* = 172)	AHSA1-High Group (*n* = 172)
Survival status		
Alive	124 (72.1%)	105 (61.0%)
Dead	48 (27.9%)	67 (40.0%)
Disease-Free Status		
Disease-Free	74 (43.0%)	63 (81.8%)
Recurred/Progressed	81 (47.1%)	81 (50.6%)
NA	17 (9.9%)	28 (16.3%)
Age		
≤65	70 (40.7%)	57 (33.1%)
>65	102 (59.3%)	115 (66.9%)
Gender		
MALE	110 (64.0%)	122 (70.9%)
FEMALE	62 (36.0%)	50 (29.1%)
Grade		
G1-G2	113 (65.7%)	100 (58.1%)
G3-G4	56 (32.6%)	70 (40.7%)
unknow	3 (1.7%)	2 (1.2%)
TNM Stage		
Stage I–II	126 (73.3%)	112 (65.1%)
Stage III–IV	35 (20.3%)	50 (29.1%)
unknow	11 (6.4%)	10 (5.8%)
T Stage		
T1-2	133 (77.3%)	121 (70.3%)
T3-4	37 (22.1%)	50 (29.1%)
TX	2 (1.2%)	1 (0.6%)
M stage		
M0	120 (69.8%)	127 (73.8%)
M1	2 (1.2%)	2 (1.2%)
MX	50 (34.3%)	43 (25.0%)
N stage		
N0	111 (64.5%)	123 (71.5%)
N1	2 (1.2%)	1 (0.6%)
NX	59 (34.3%)	48 (27.9%)
AFP level		
<400	113 (65.7%)	86 (50.5%)
≥400	26 (15.1%)	34 (19.8%)
NA	33 (19.2%)	52 (30.2%)
Vascular Invasion		
Yes	45 (26.2%)	52 (30.3%)
None	107 (62.2%)	85 (49.4%)
NA	20 (11.6%)	35 (20.3%)

NA, Not Available; AFP, Alpha fetoprotein.

**Table 2:** Univariate and multivariate Cox regression analysis of AHSA1 and clinical characteristics in HCC patients from the TCGA-LIHC database.

Clinical Characteristics	Univariate Cox Regression Analysis		Multivariate Cox Regression Analysis	
	OR (95%CI)	*p* Value	OR (95%CI)	*p*-Value
Age	1.010 (0.995–1.025)	0.181		
Gender	0.824 (0.560–1.214)	0.328		
pathological grade	1.124 (0.871–1.450)	0.368		
TNM stage	1.674 (1.361–2.059)	<0.001	1.606 (1.300–1.984)	<0.001
AHSA1 expression	1.035 (1.021–1.049)	<0.001	1.030 (1.016–1.045)	<0.001

OR, Odds Ratio; CI, Confidence Interval.

**Table 3:** Correlation between AHSA1 expression in tumor tissues and clinicopathological characteristics of patients in HCC microarray.

Clinicopathological Characteristics		Total (*n* = 90)	AHSA1 Expression (*n*)		*p*-Value
			Low Expression	High Expression	
Gender					
	Male	80	31	49	0.494
	Female	10	5	5	
Age					
	≥60	22	9	13	0.920
	<60	68	27	41	
HBsAg					
	Positive	70	25	45	0.081
	Negative	19	11	8	
HCV-Ab					
	Positive	80	33	47	0.934
	Negative	7	3	4	
AFP (ug/L)					
	≥400	32	13	19	0.604
	<400	57	20	37	
ALT (U/L)					
	≥100	7	3	4	0.892
	<100	82	33	49	
TBiL (umol/L)					
	≥17.1	24	8	16	0.406
	<17.1	65	28	37	
Primary tumor (T)					
	T1	63	27	36	0.320
	T2	24	9	15	
	T3	3	0	3	
Tumor size (cm)					
	≥4	48	17	31	0.343
	<4	42	19	23	
recrudescence					
	Yes	48	11	37	<0.001
	No	41	25	16	

A χ^2^ test or Fisher’s exact test was applied to access the associations between the expression of AHSA1 and the clinicopathologic characteristics of HCC patients in the microassay. AFP, Alpha fetoprotein; ALT, Alanine Aminotransferase; TBiL, Total Bilirubin.

## Data Availability

Data required for bioinformatics analysis were downloaded from the GEO (GSE14520 and GSE50579, https://www.ncbi.nlm.nih.gov/geo) and TCGA-LIHC (https://www.cancer.gov/about-nci/organization/ccg/research/structural-genomics/tcga) databases.

## Supplementary Materials

The following supporting information can be downloaded at: https://www.mdpi.com/article/10.3390/cancers14194600/s1, Figure S1. A. The AHSA1 level was positively correlated with TNM stage of HCC patients in TCGA-LIHC. database. B. The AHSA1 level was positively correlated with pathological grade of HCC patients in TCGA-LIHC database. C–G. The relationship between AHSA1 level and the top five genes with the most frequent mutations in HCC including TERT (*p* > 0.05), TP53 (*p* < 0.05), CTNNB1 (*p* < 0.001), AXIN1 (*p* > 0.05), and ARID1A (*p* < 0.05). Figure S2. A. The mRNA level of AHSA1 in HCC cells and normal immortalized liver epithelial cells (LO2). B. Representative images of tumor tissue fluorescence and bright field in nude mice. C and D. Representative images and corresponding quantitative analysis of Transwell invasion assays in indicated HCC cell lines. E. KEGG and GO enrichment analysis of AHSA1-related genes in TCGALIHC database. **, *p* < 0.01; ***, *p* < 0.001. Figure S3. A. The AHSA1-ERK1/2-CALD1 interaction was analyzed by Co-IP of Huh-7 cell lysates, and shown by Western blot. B. IHC score of CALD1 in cancer and adjacent tissues of HCC patients. C. The effect of knocking-down or overexpressing AHSA1 on MEK1/2 expression in HCC cells. D. Effect of knock-down HSP90 on the corresponding protein molecules in Hep3B cells. E. The effects of different concentrations of SCH772984 on HCC cells were measured by CCK-8 assay. ***, *p* < 0.001. Original western blots are in Supplementary Materials.
