# Supplementary material for: AHSA1 Promotes Proliferation and EMT by Regulating ERK/CALD1 Axis in Hepatocellular Carcinoma
Source: Cancers (Basel). 2022 Sep 22;14(19):4600. doi: 10.3390/cancers14194600 (PMC9562867; doi:10.3390/cancers14194600)
Supplement: Supplementary file 1 [file cancers-14-04600-s001.zip › cancers-1858286-supplementary.pdf]

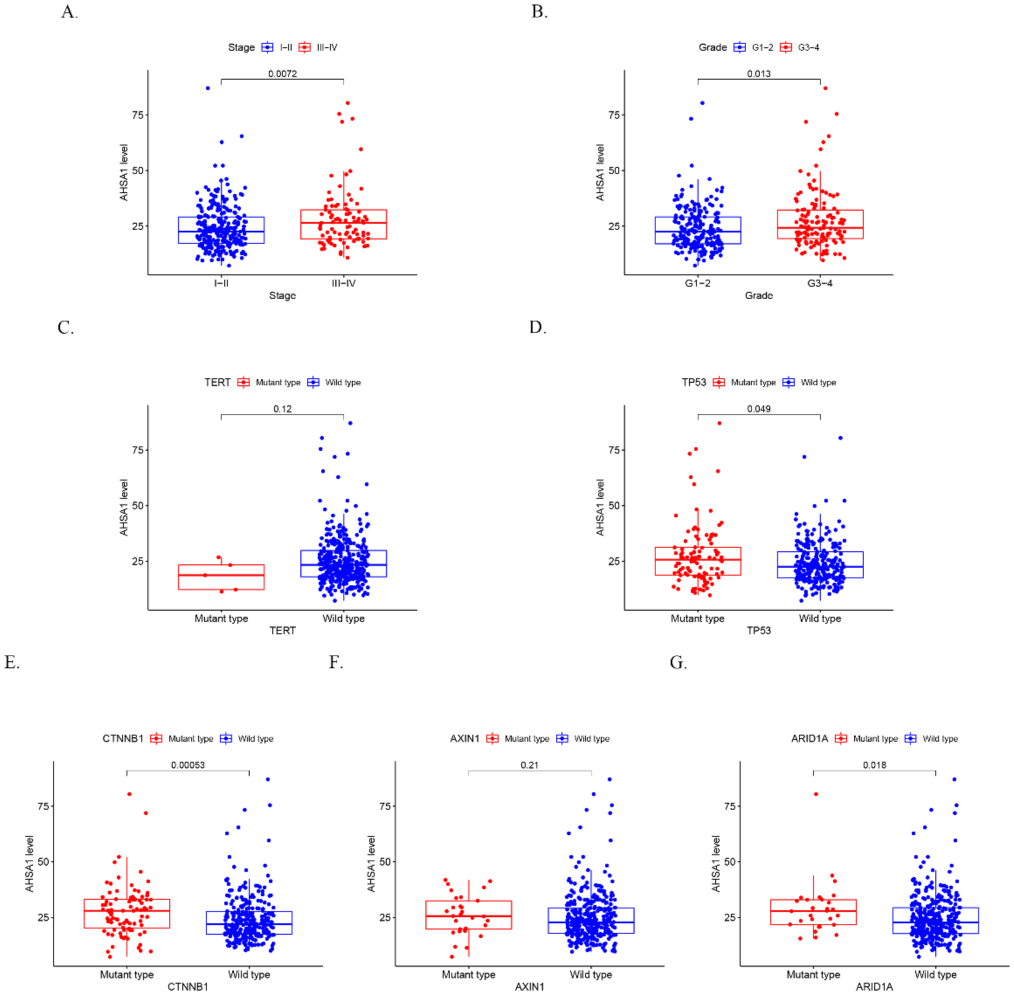

Figure S1.

(**A**). The AHSA1 level was positively correlated with TNM stage of HCC patients in TCGA-LIHC database. (**B**). The AHSA1 level was positively correlated with pathological grade of HCC patients in TCGA-LIHC database. (**C–G**). The relationship between AHSA1 level and the top five genes with the most frequent mutations in HCC including TERT (P>0.05), TP53(P<0.05), CTNNB1(P<0.001), AXIN1(P>0.05), and ARID1A(P<0.05).

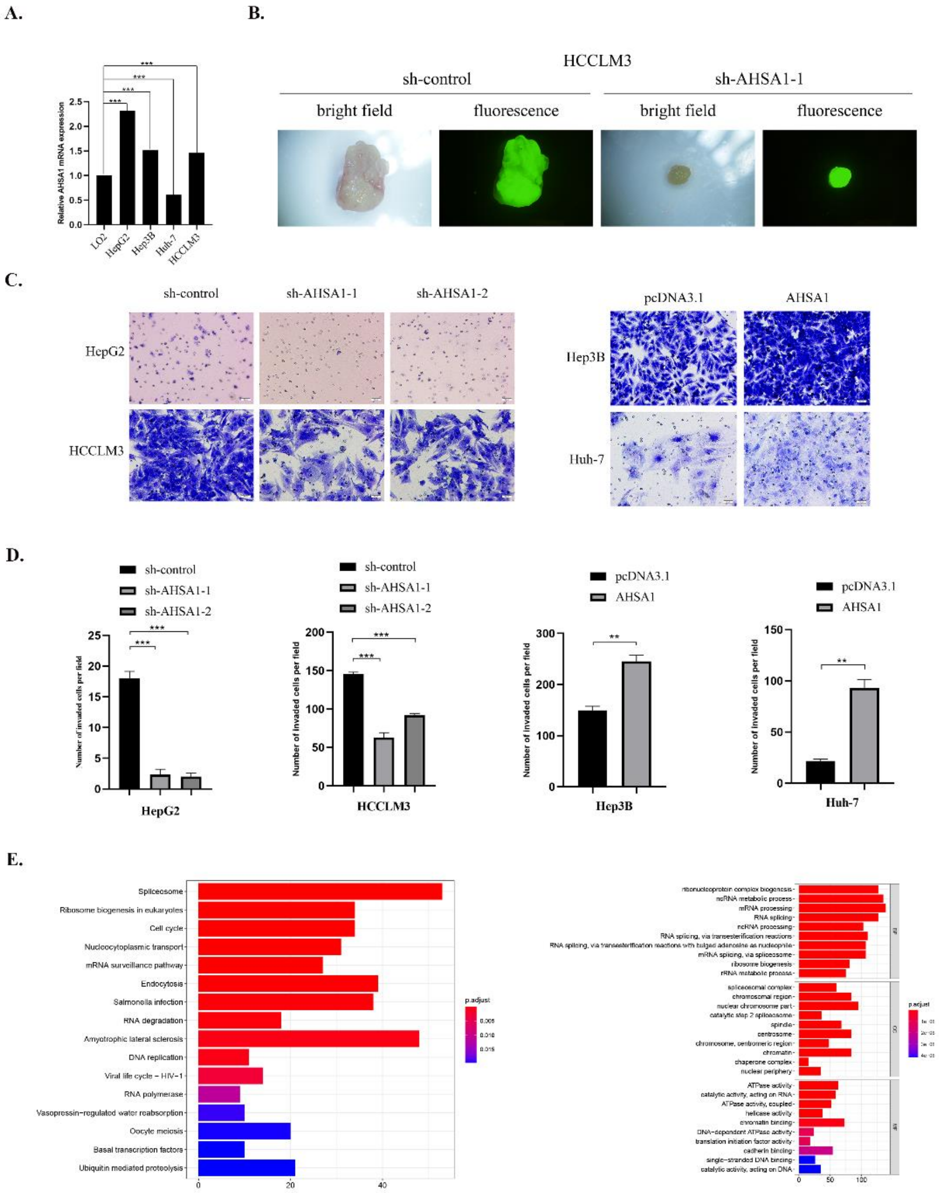

Figure S2.

(**A**). The mRNA level of AHSA1 in HCC cells and normal immortalized liver epithelial cells (LO2). (**B**). Representative images of tumor tissue fluorescence and bright field in nude mice. (**C**,**D**). Representative images and corresponding quantitative analysis of Transwell invasion assays in indicated HCC cell lines. (**E**). KEGG and GO enrichment analysis of AHSA1-related genes in TCGA-LIHC database. **, $P < 0.01$; ***, $P < 0.001$.

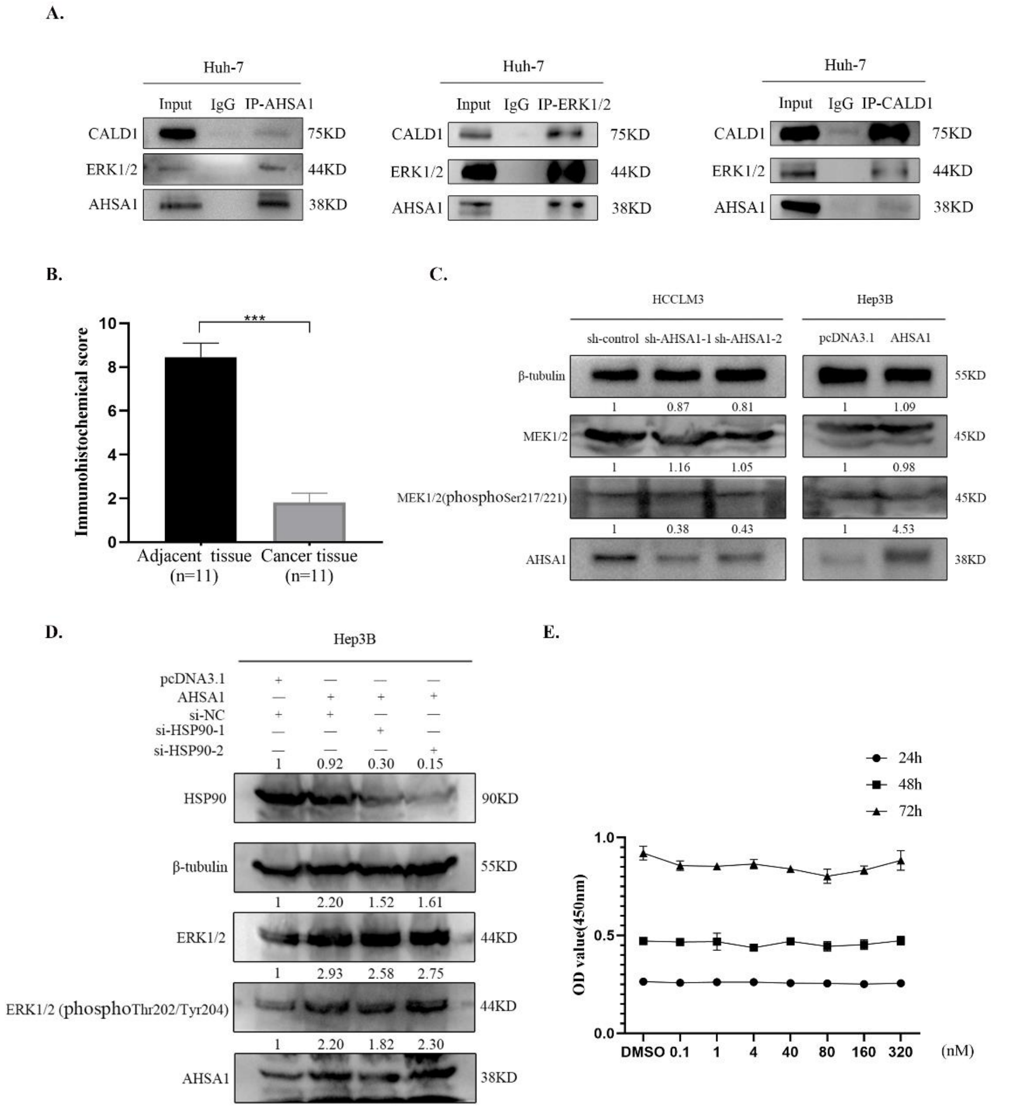

Figure S3.

(**A**). The AHSA1-ERK1/2-CALD1 interaction was analyzed by Co-IP of Huh-7 cell lysates, and shown by Western blot. (**B**). IHC score of CALD1 in cancer and adjacent tissues of HCC patients. (**C**). The effect of knocking-down or overexpressing AHSA1 on MEK1/2 expression in HCC cells. (**D**). Effect of knock-down HSP90 on the corresponding protein molecules in Hep3B cells. (**E**). The effects of different concentrations of SCH772984 on HCC cells were measured by CCK-8 assay. ***, $P < 0.001$.

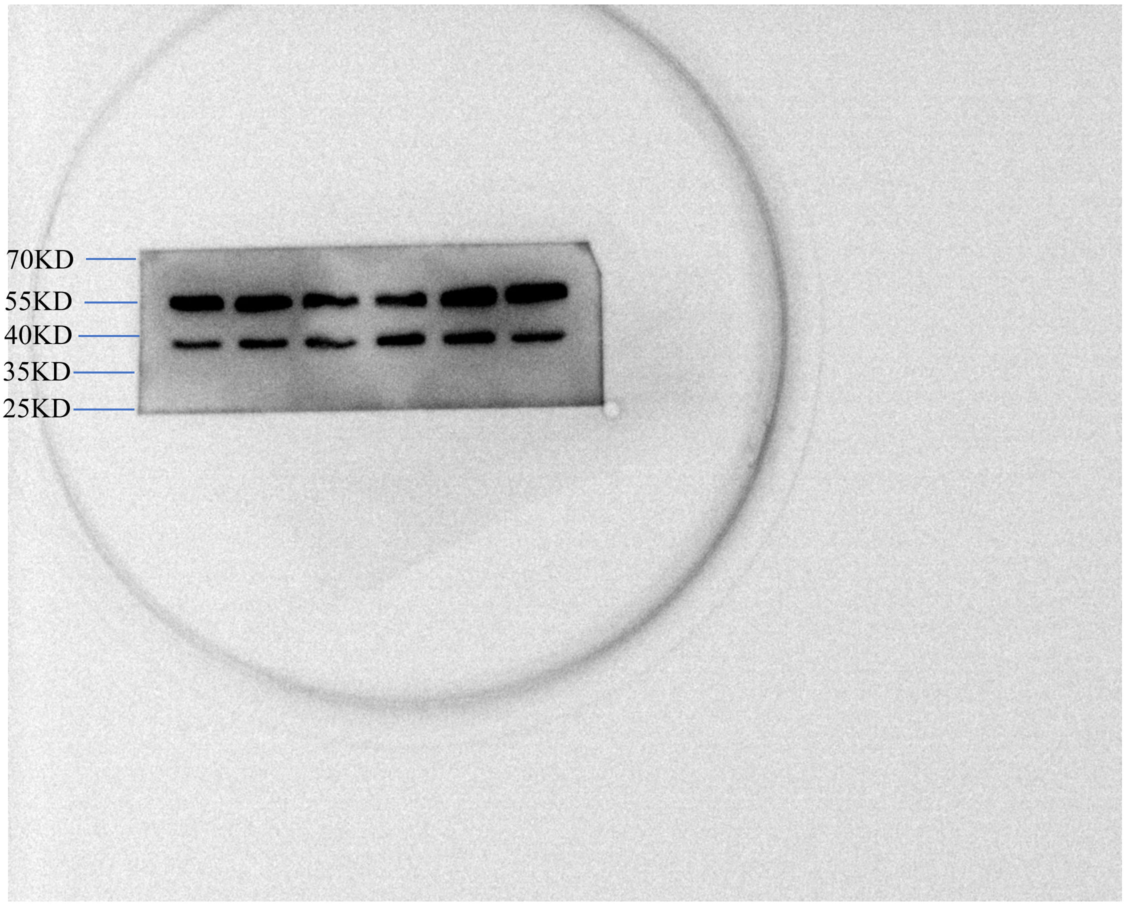

Figure 1C

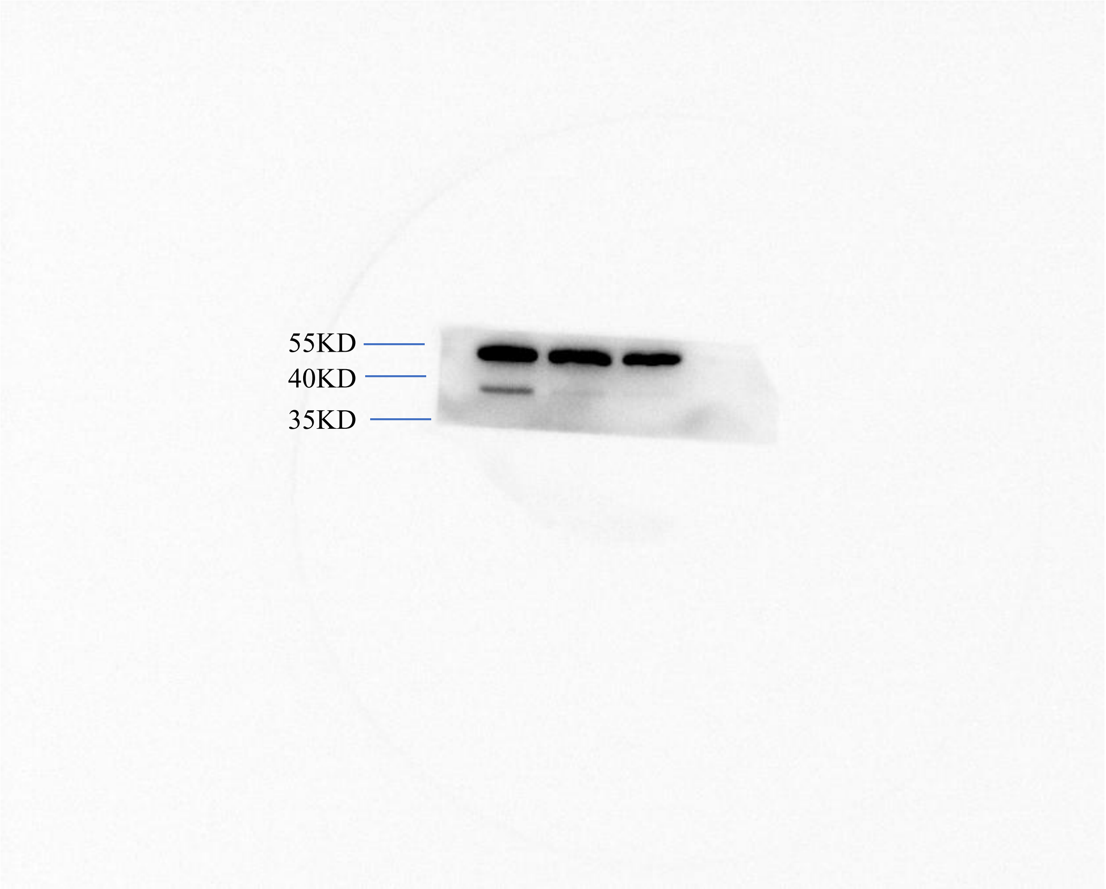

Figure2A HepG2 β-tubulin
Figure2A HepG2 AHSA1

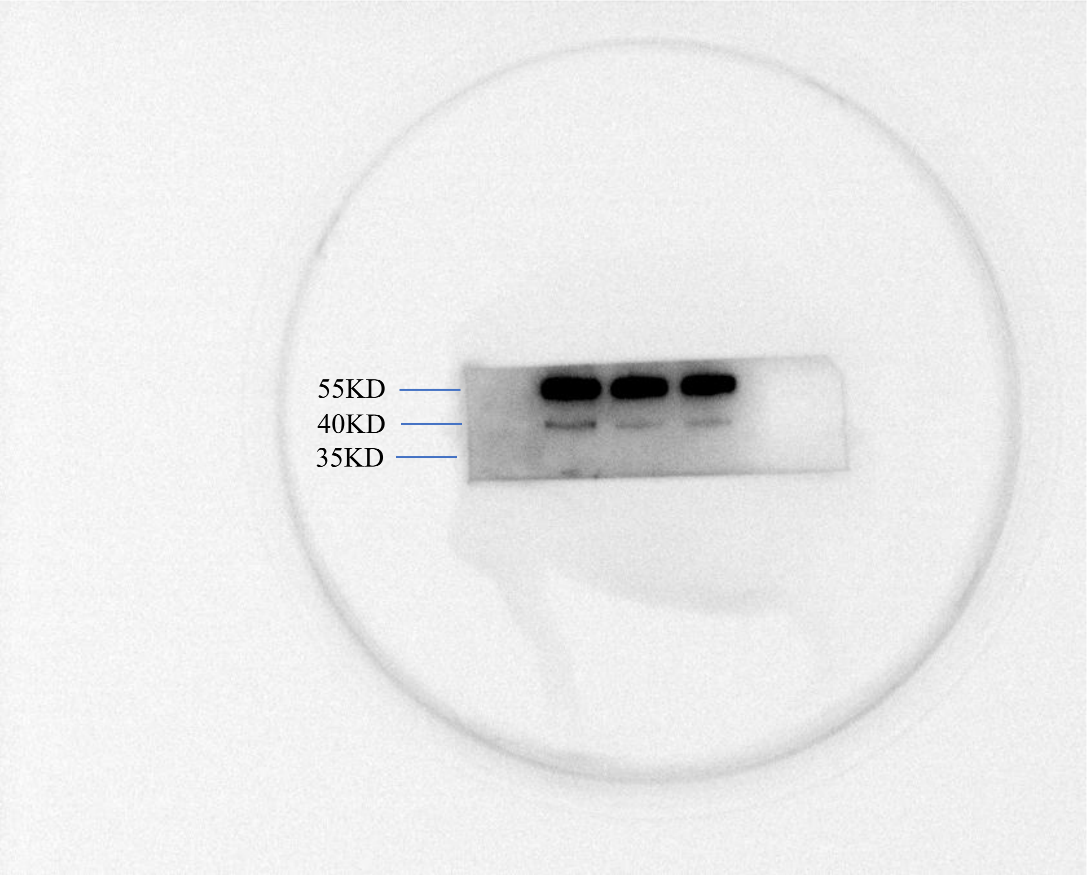

Figure2A HCCLM3 β-tubulin
Figure2A HCCLM3 AHSA1

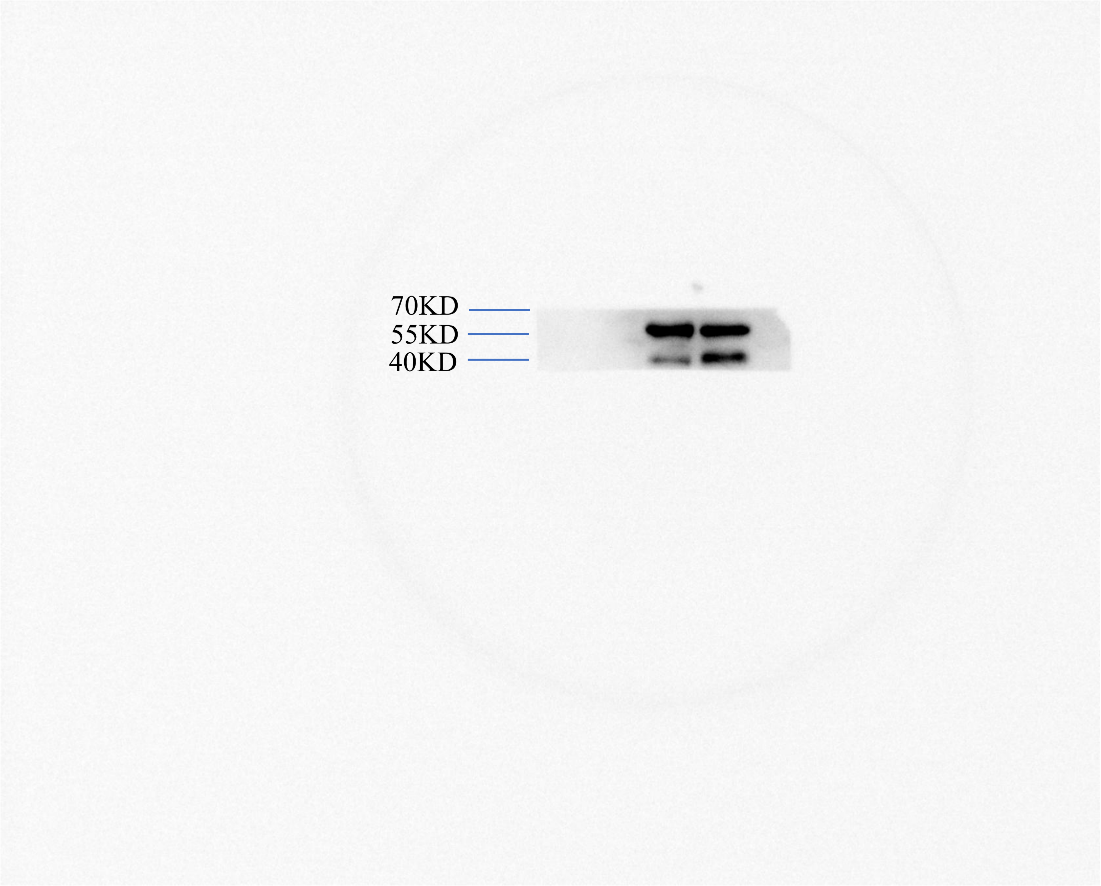

Figure2A Huh-7 β-tubulin
Figure2A Huh-7 AHSA1

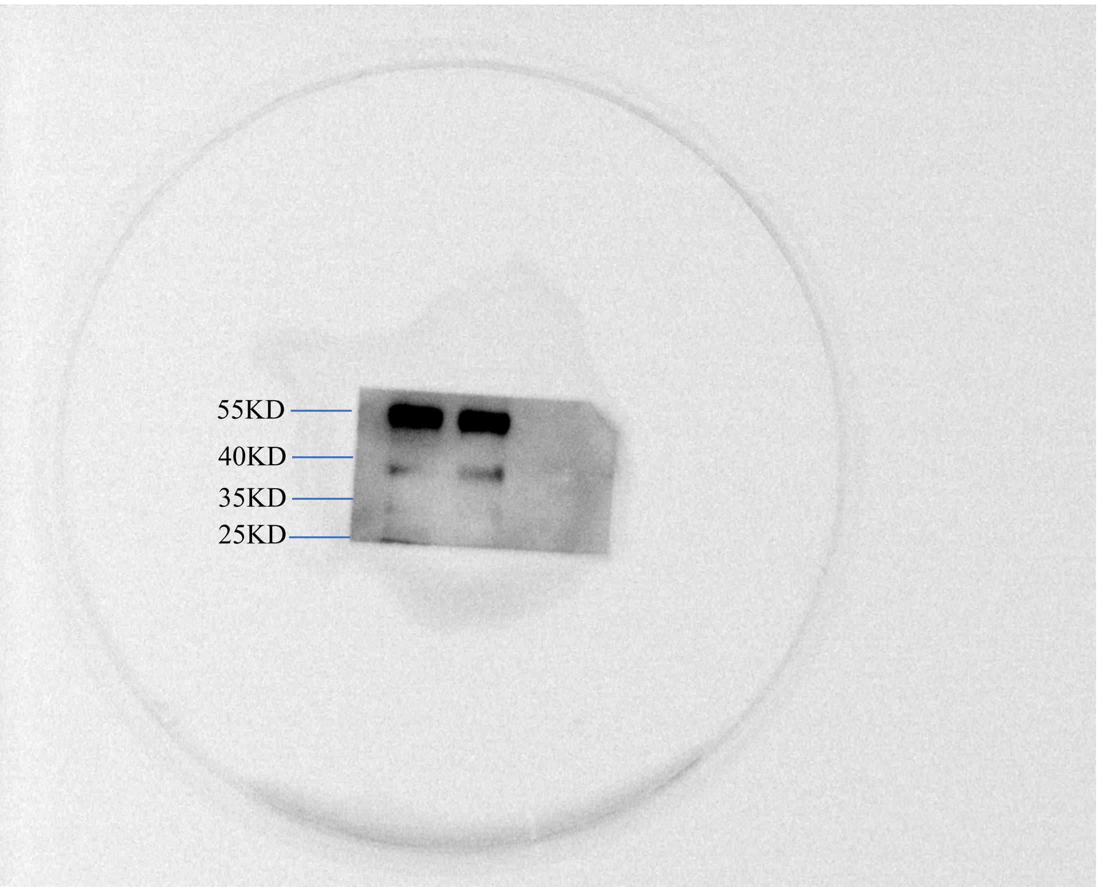

Figure2A Hep3B β-tubulin

Figure2A Hep3B AHSA1

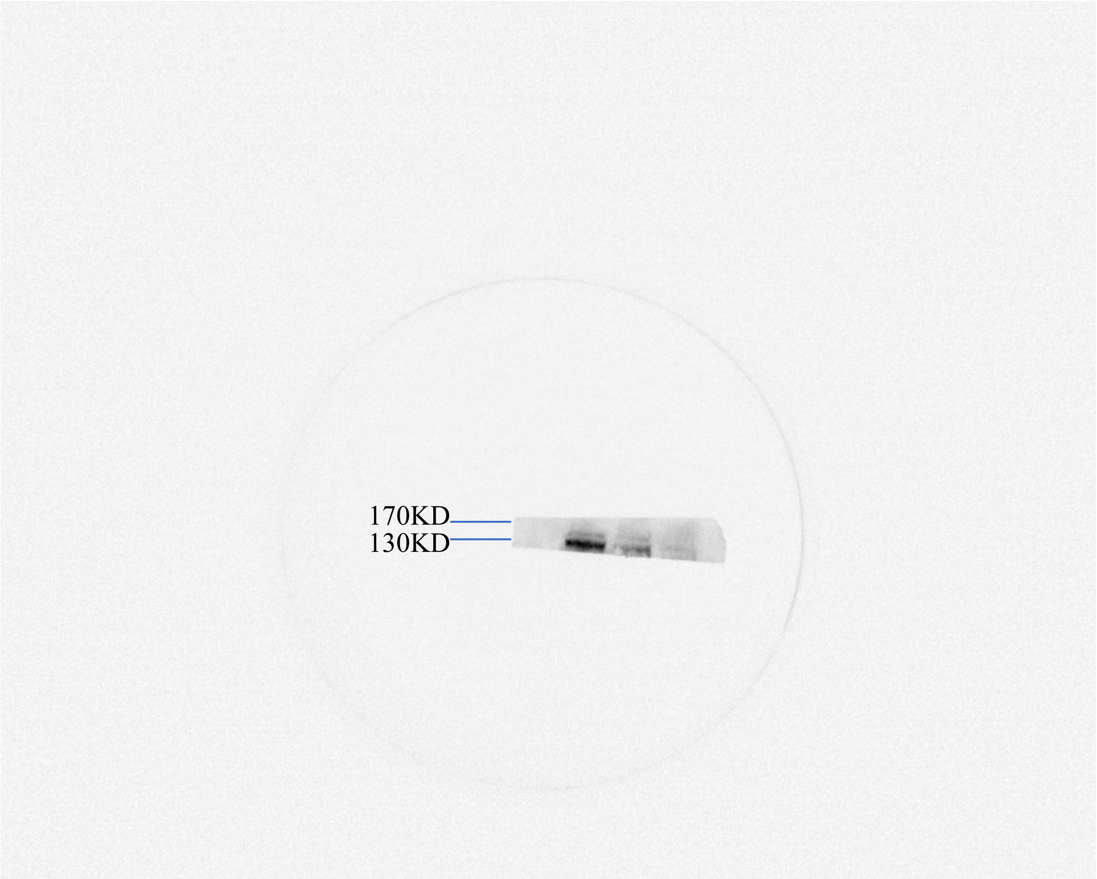

Figure4A HepG2 N-cadherin

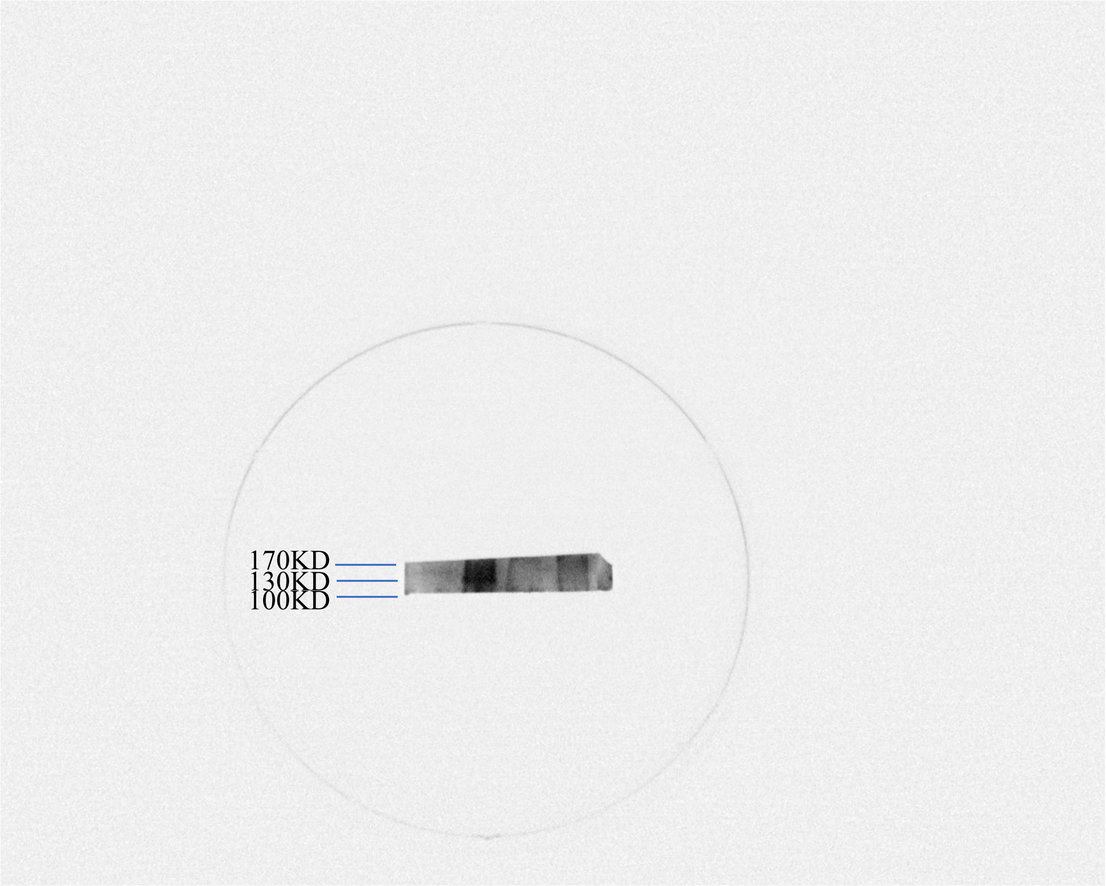

Figure4A HCCLM3 N-cadherin

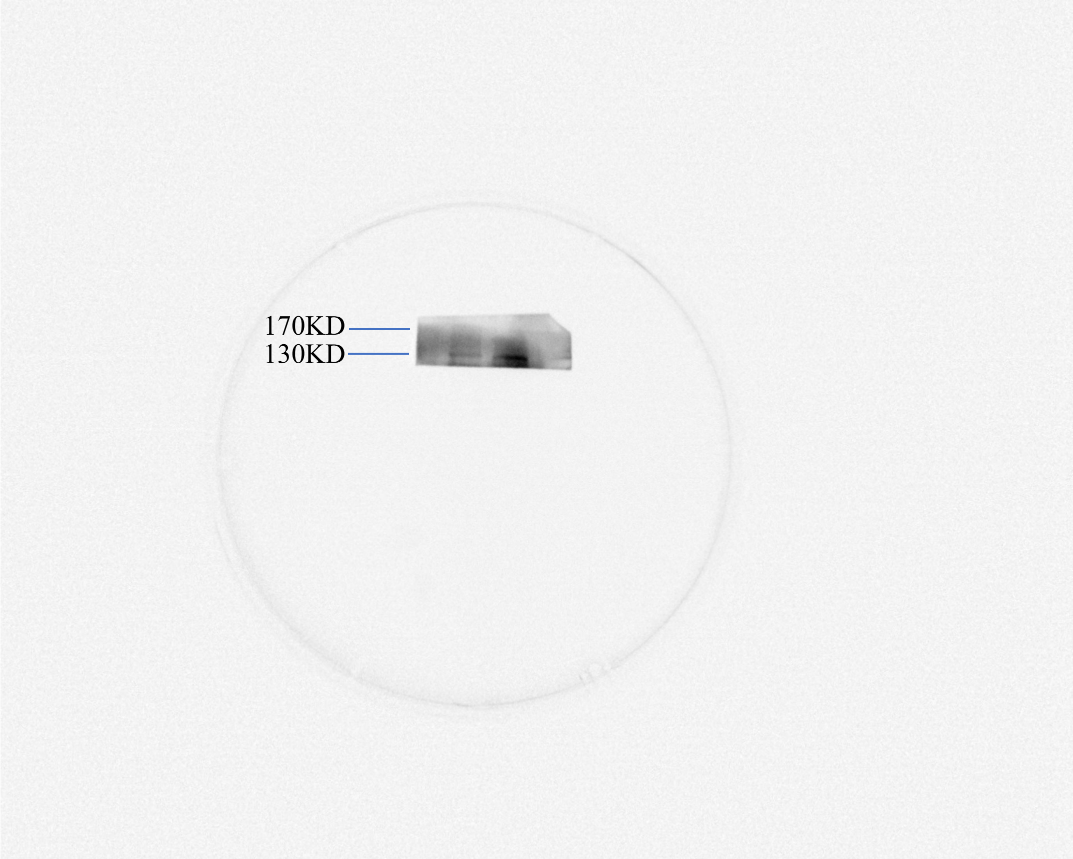

Figure4A Hep3B N-cadherin

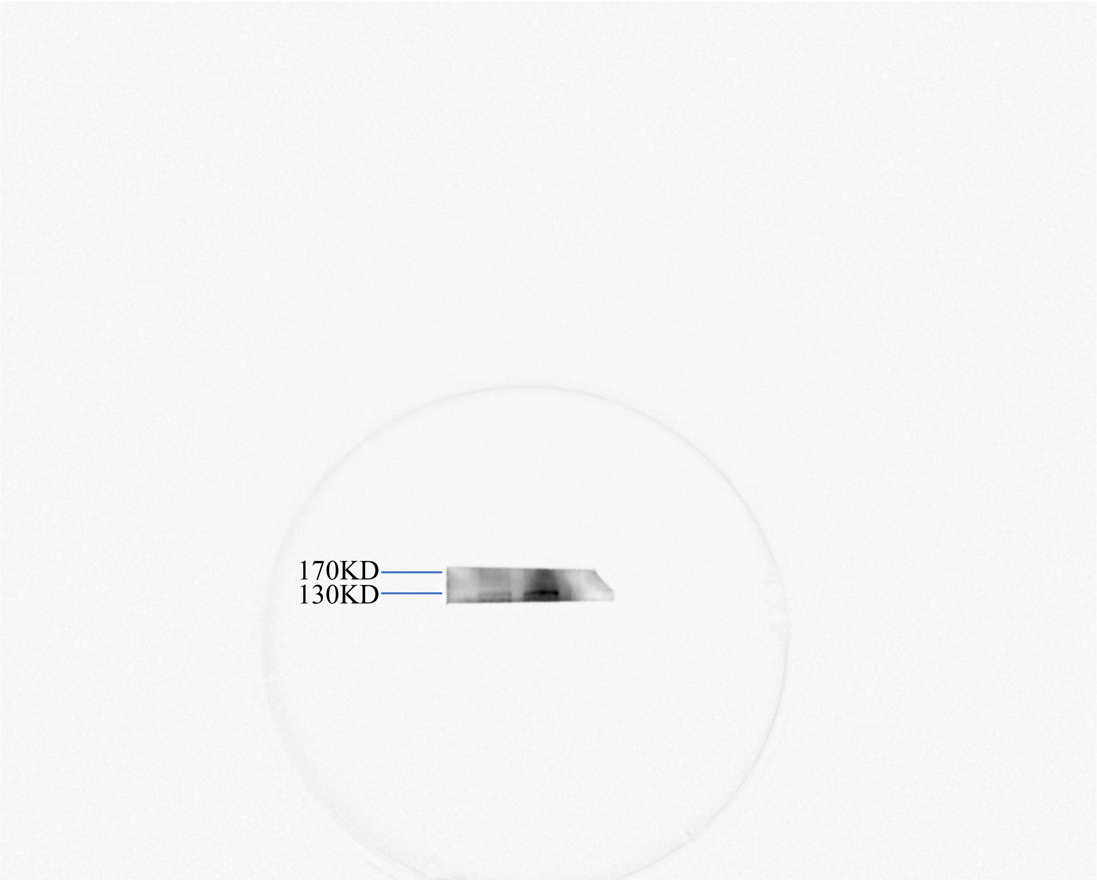

Figure4A Huh-7 N-cadherin

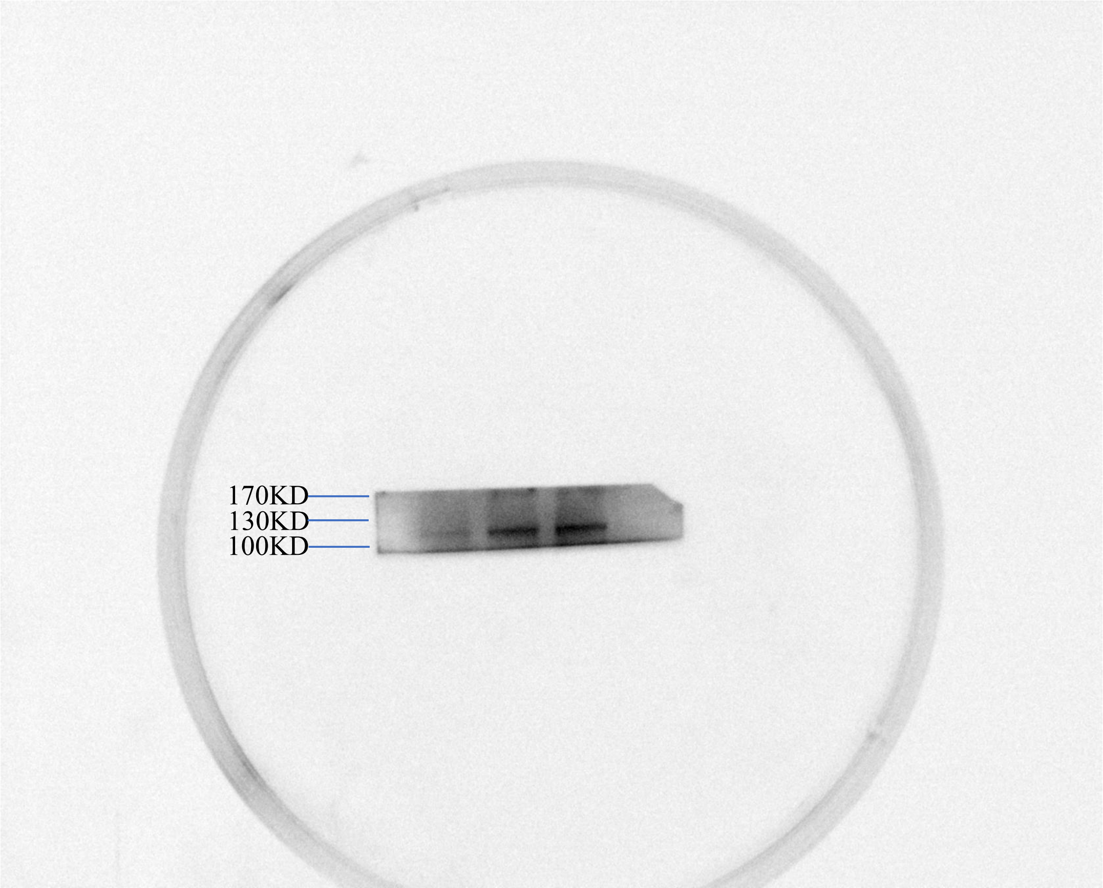

Figure4A　　HepG2　E-cadherin

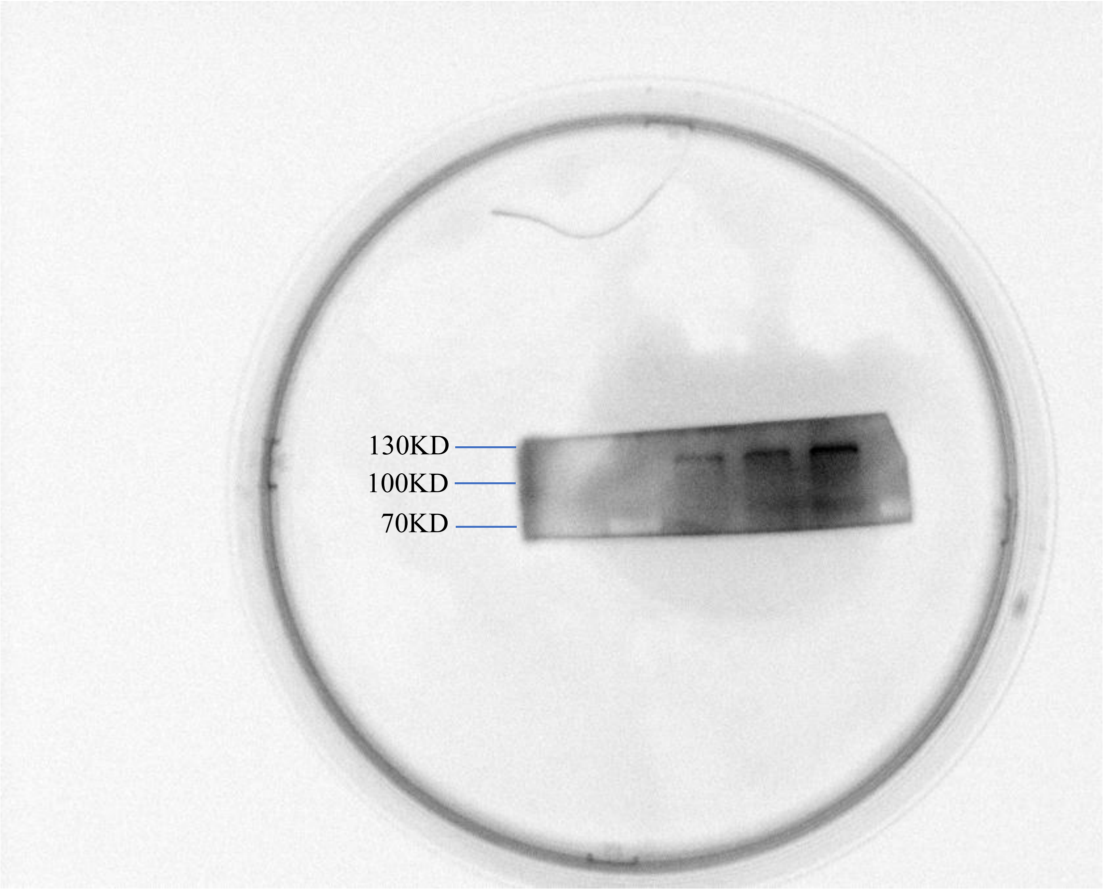

Figure4A HCCLM3 E-cadherin

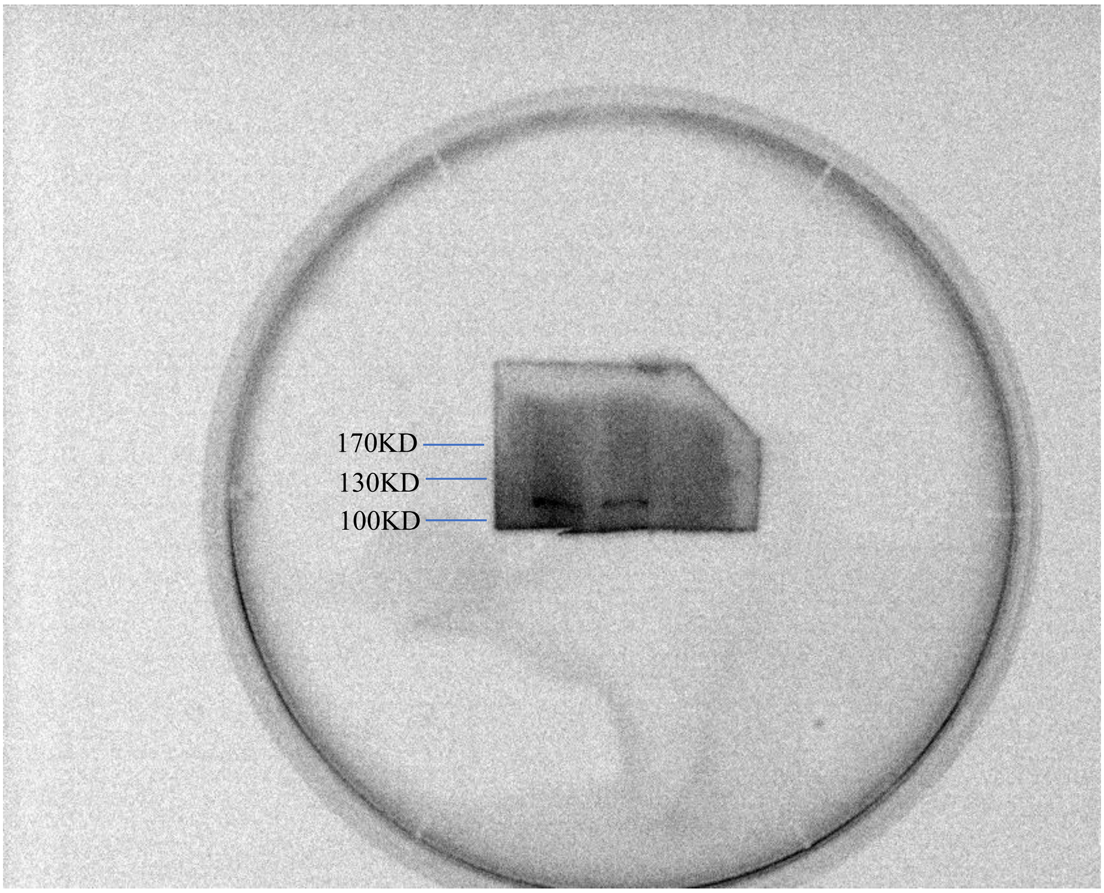

Figure4A Hep3B E-cadherin

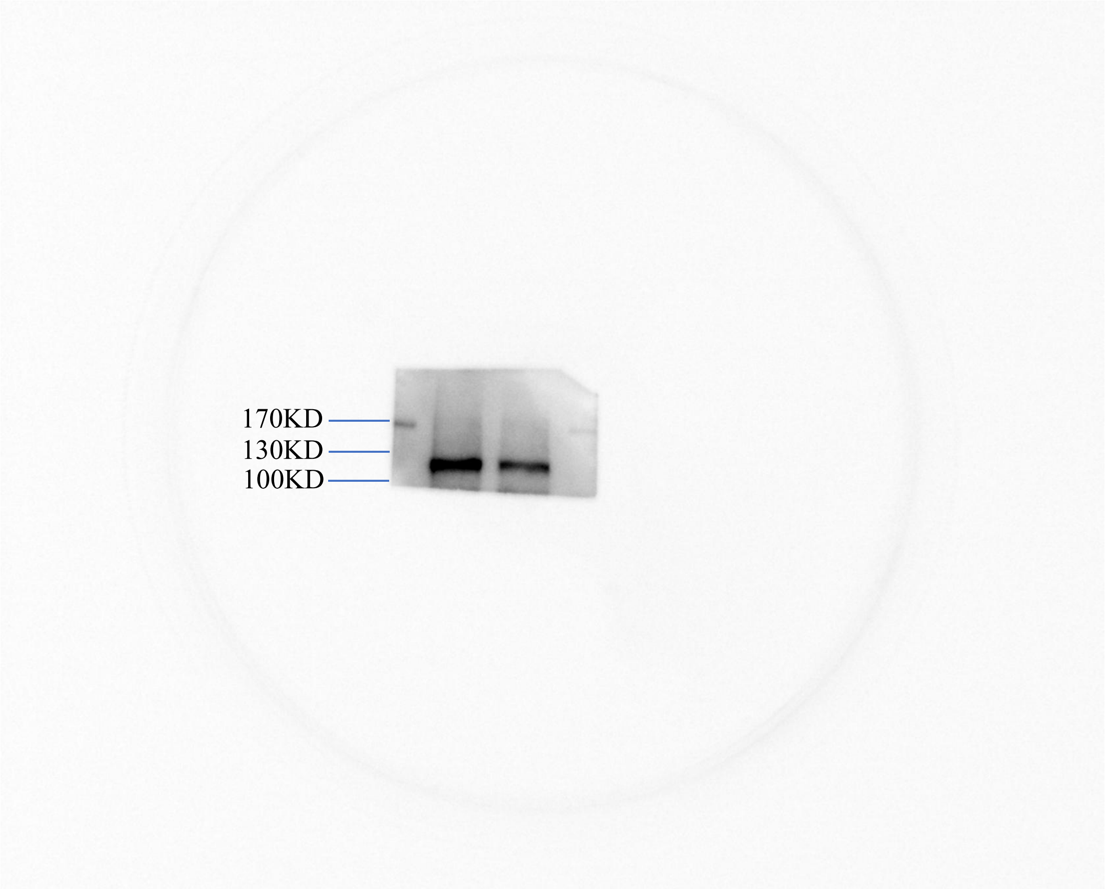

Figure4A Huh-7 E-cadherin

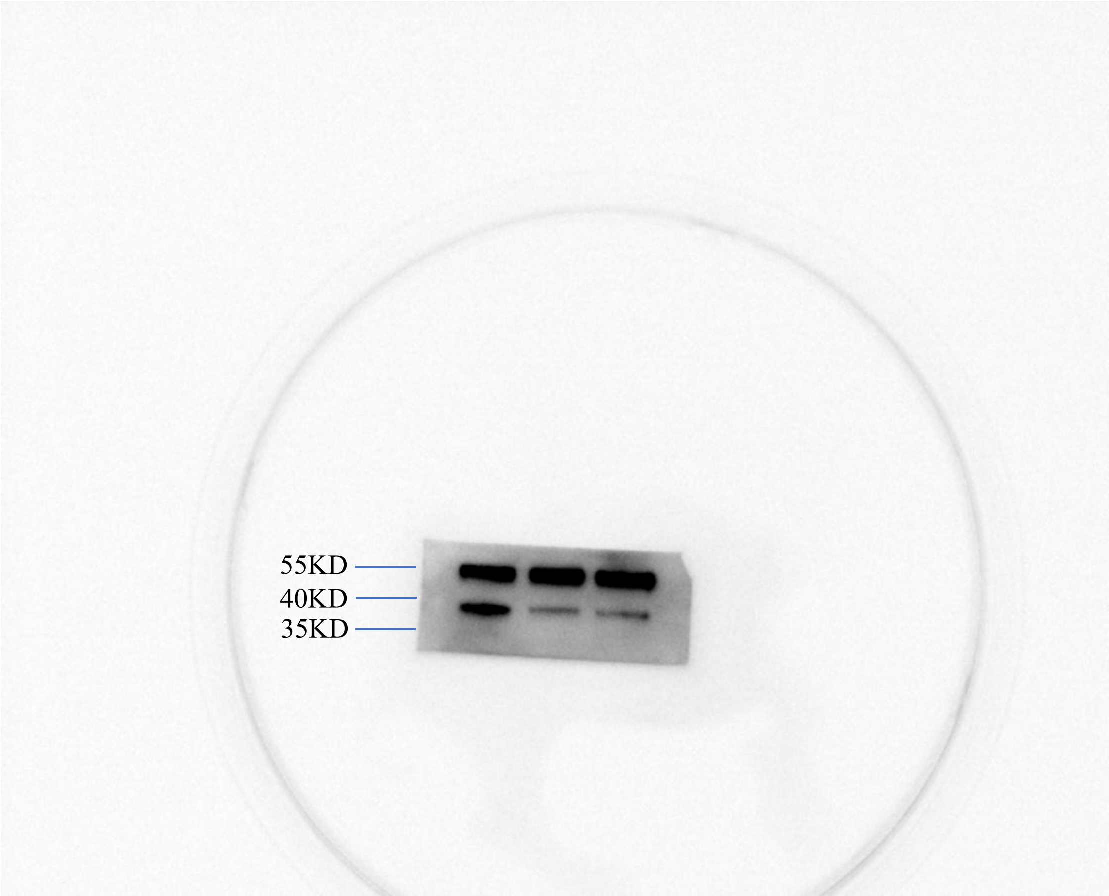

Figure4A HepG2 β-tubulin

Figure4A HepG2 AHSA1

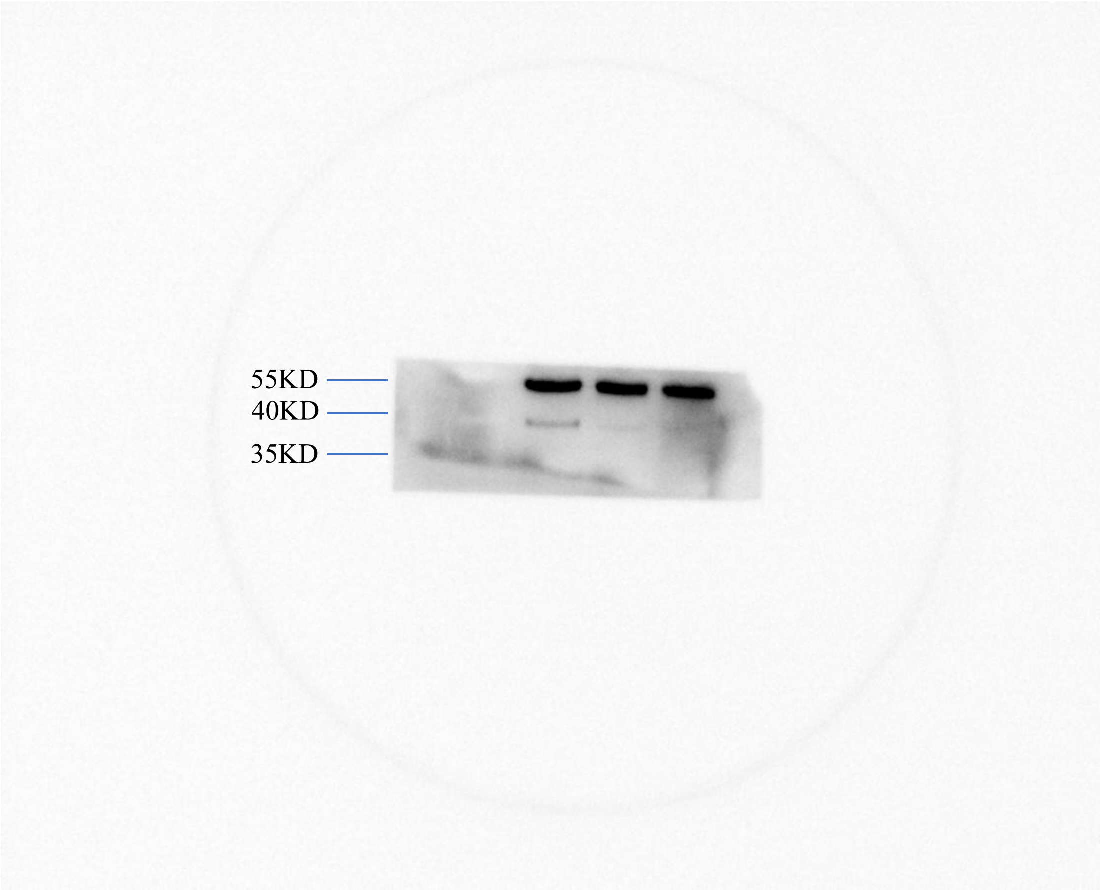

Figure4A HCCLM3 β-tubulin

Figure4A HCCLM3 AHSA1

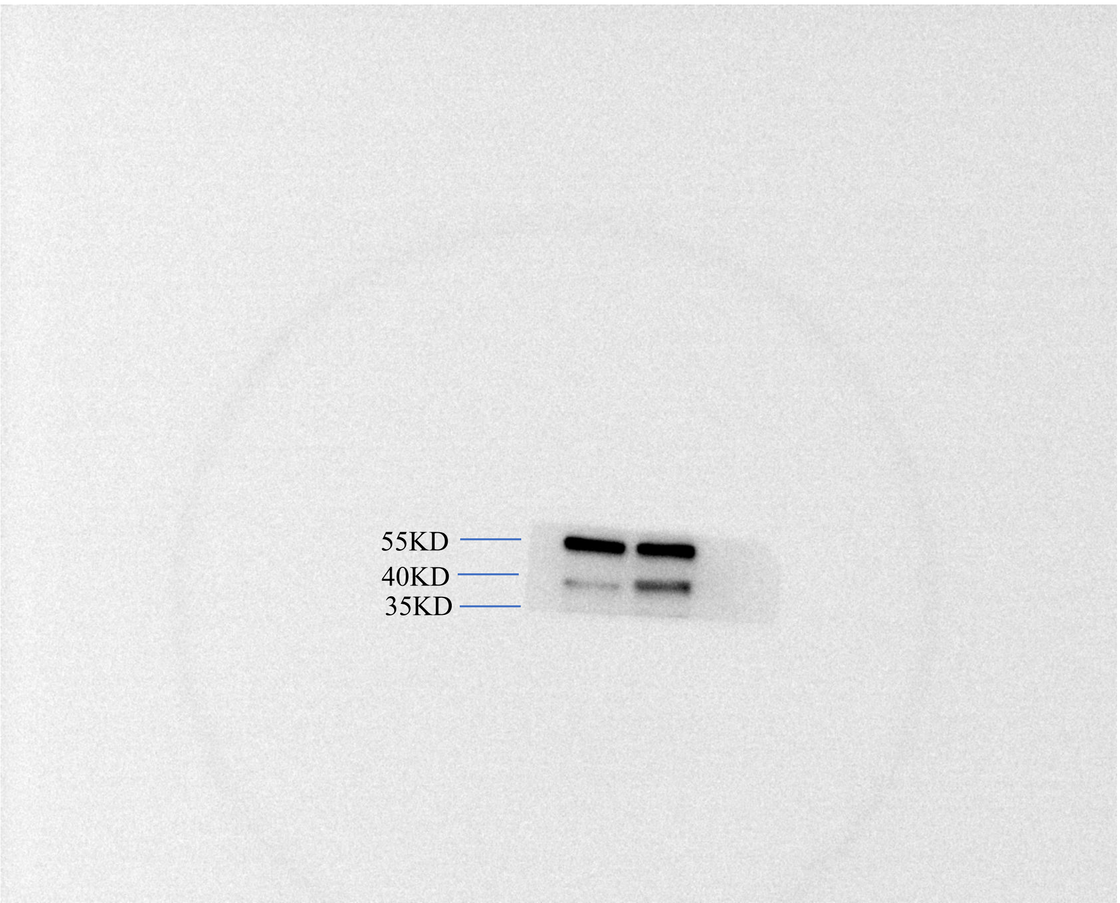

Figure4A Hep3B β-tubulin

Figure4A Hep3B AHSA1

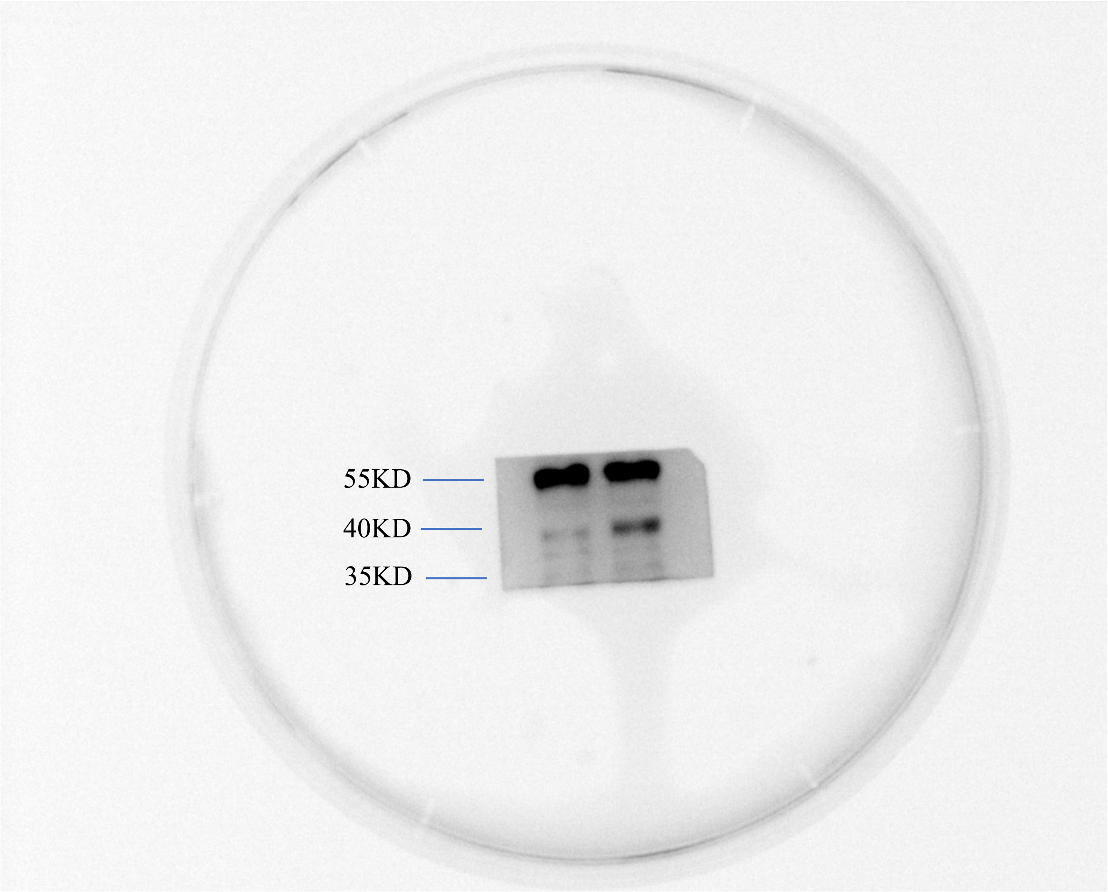

Figure4A　　Huh-7　β-tubulin

Figure4A　　Huh-7　AHSA1

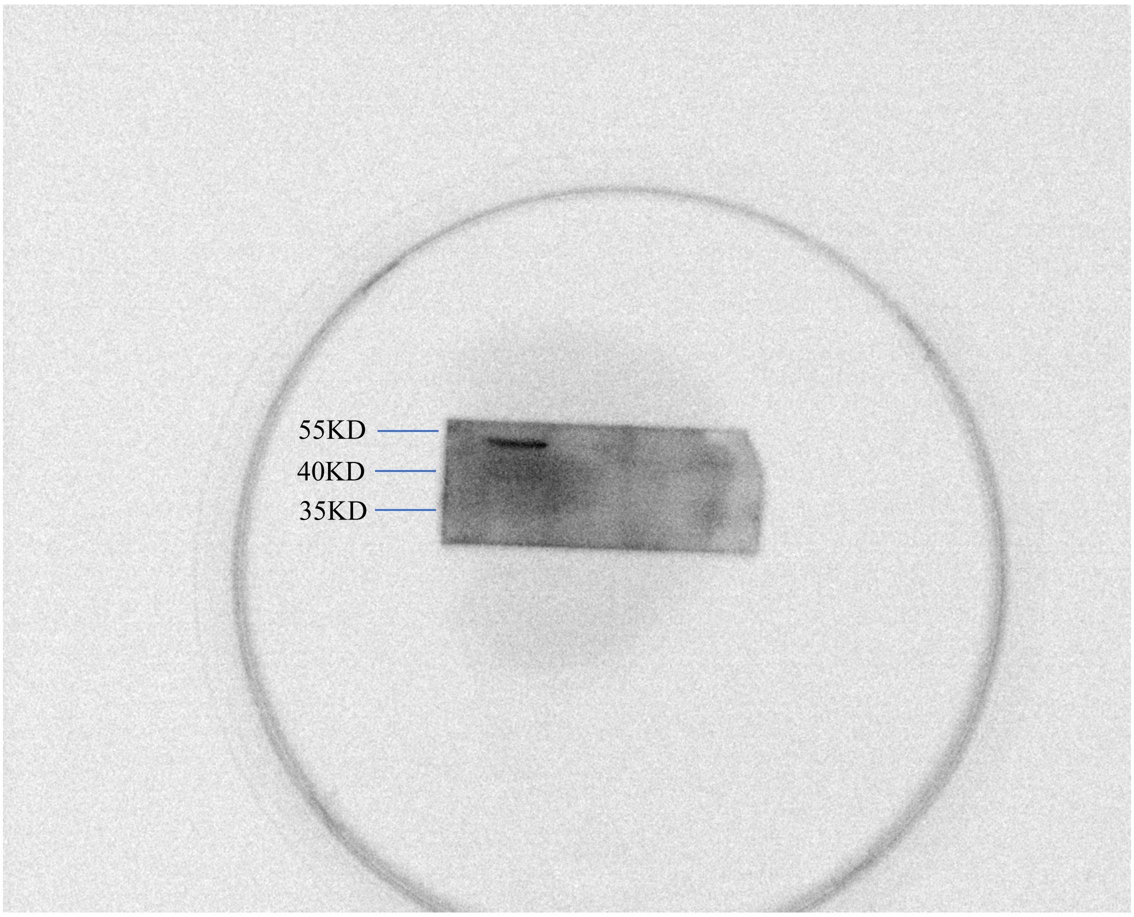

Figure4A HepG2 vimentin

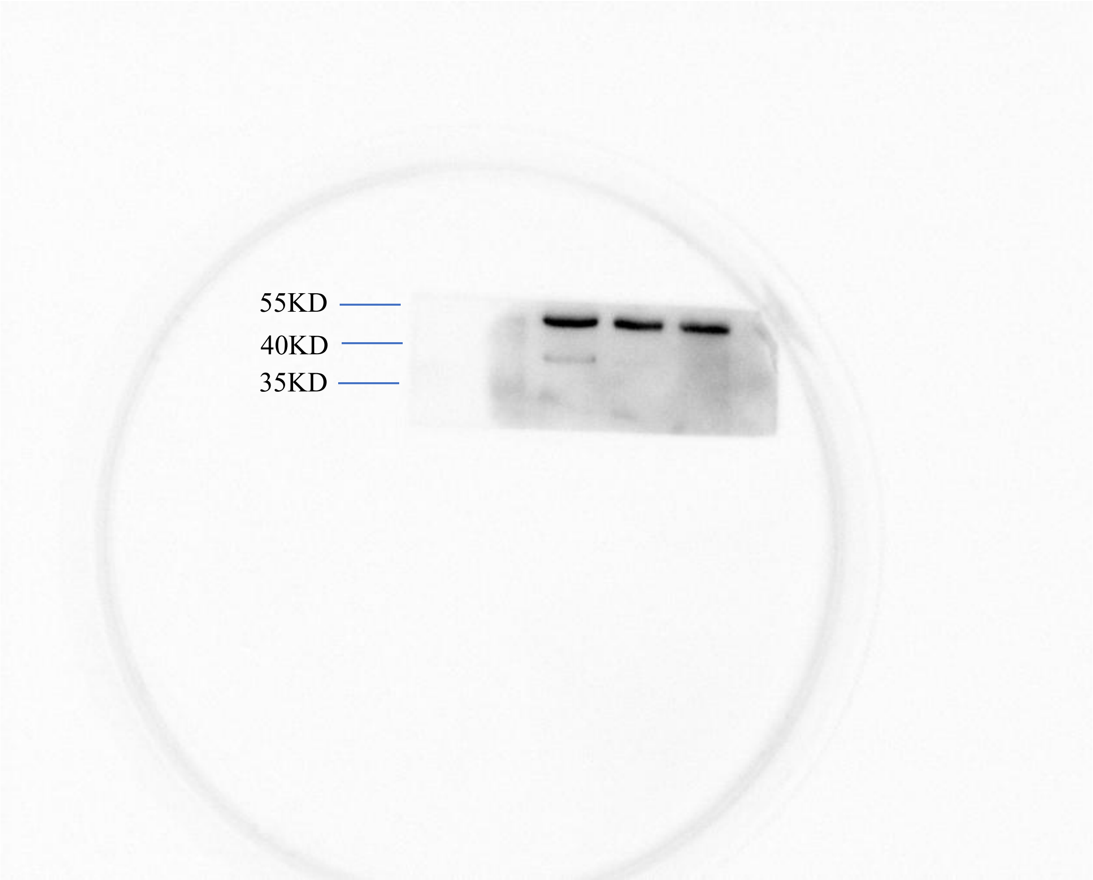

Figure4A HCCLM3 vimentin

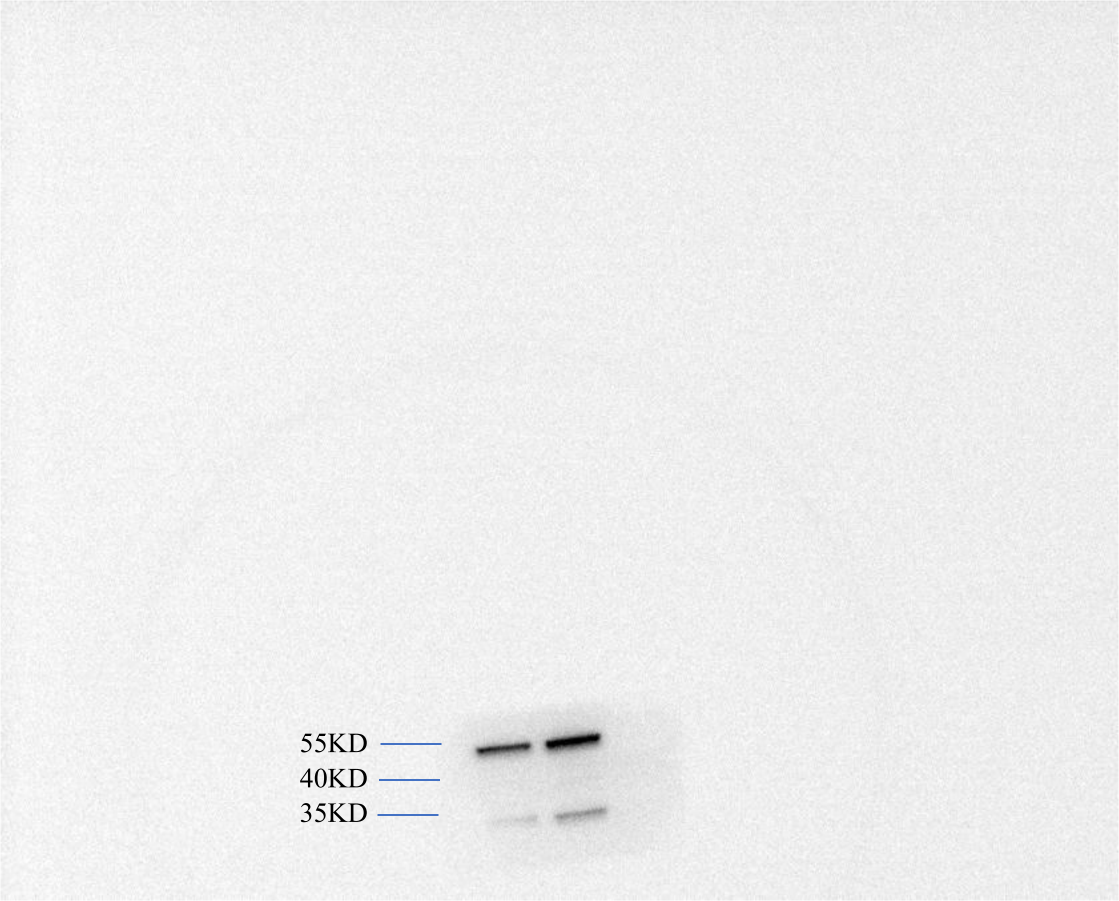

Figure4A Hep3B vimentin

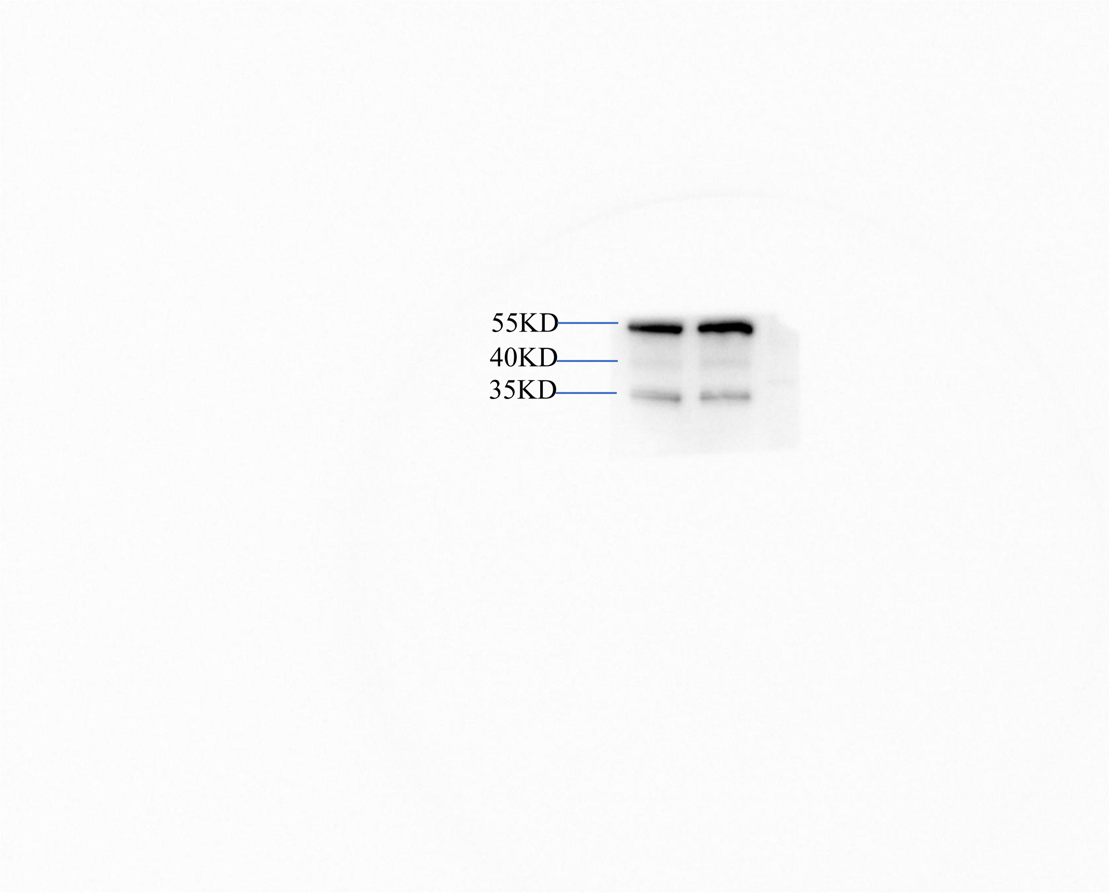

Figure4A　　Huh-7　vimentin

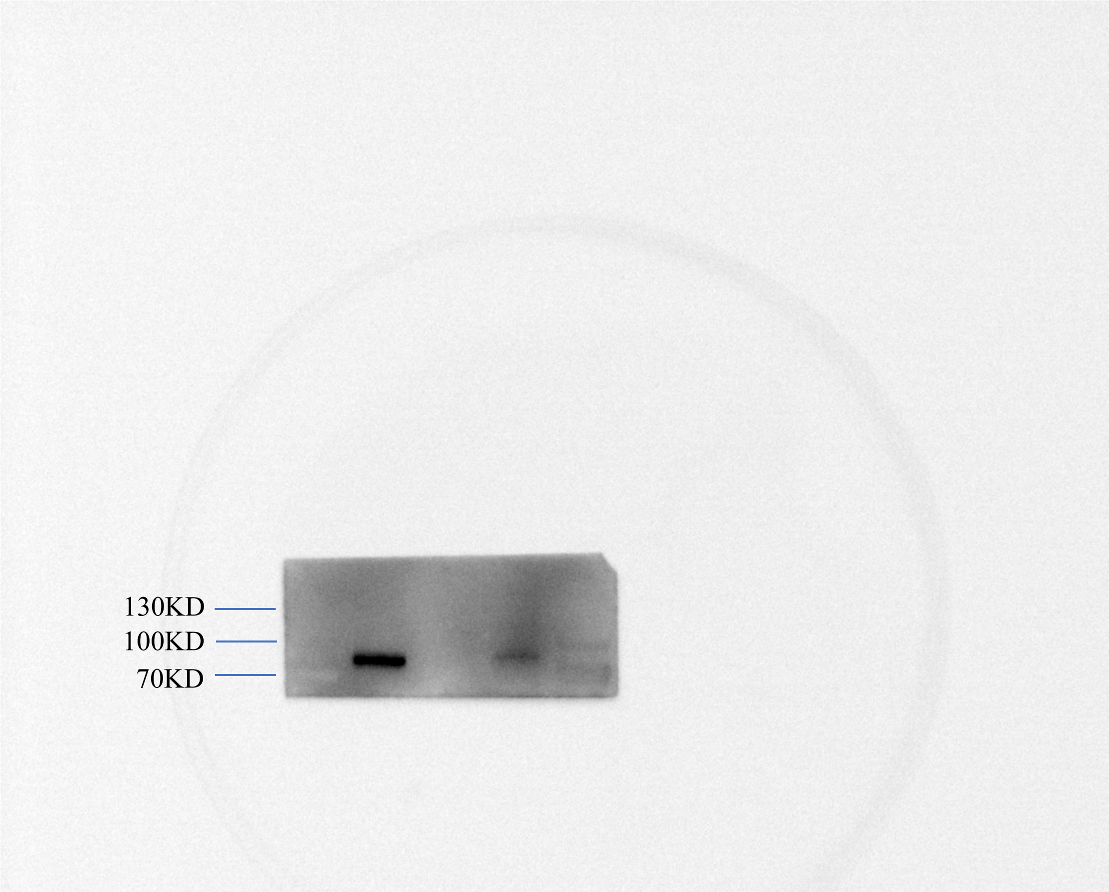

Figure4C Hep3B IP-AHSA1 CALD1

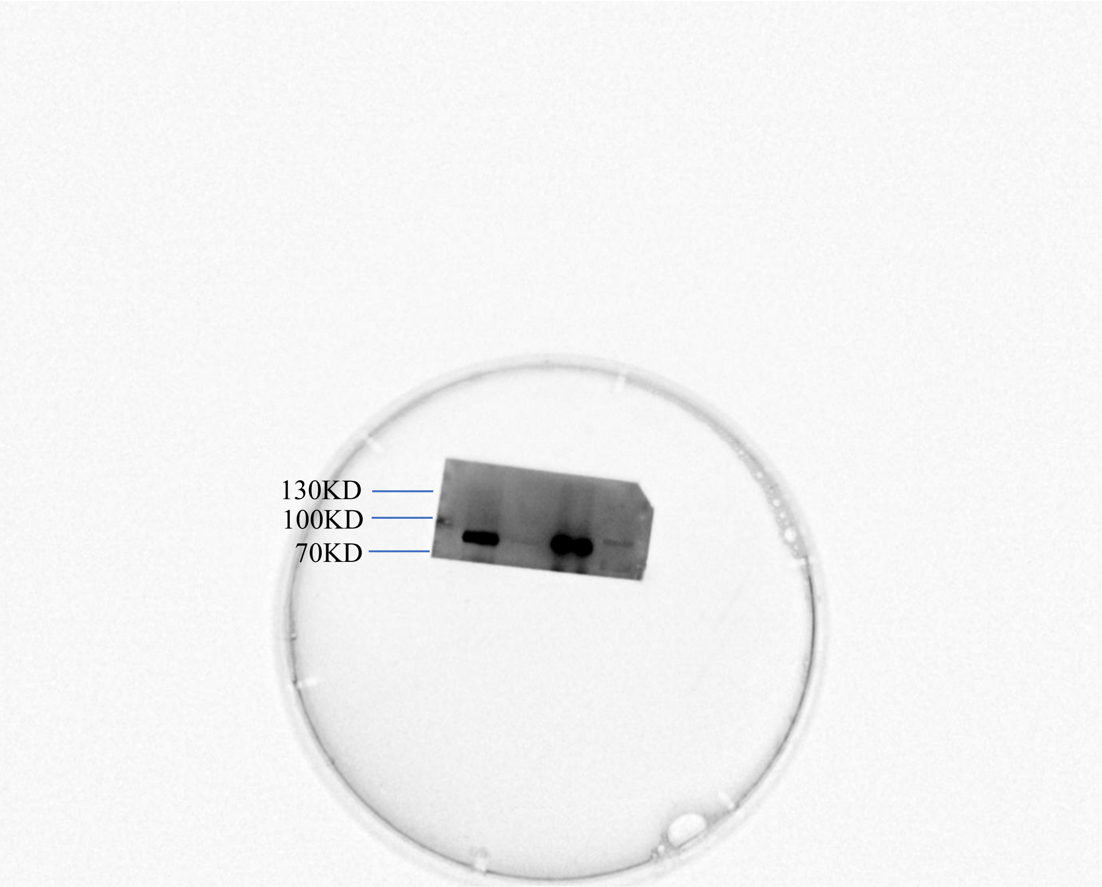

Figure4C Hep3B IP-CALD1 CALD1

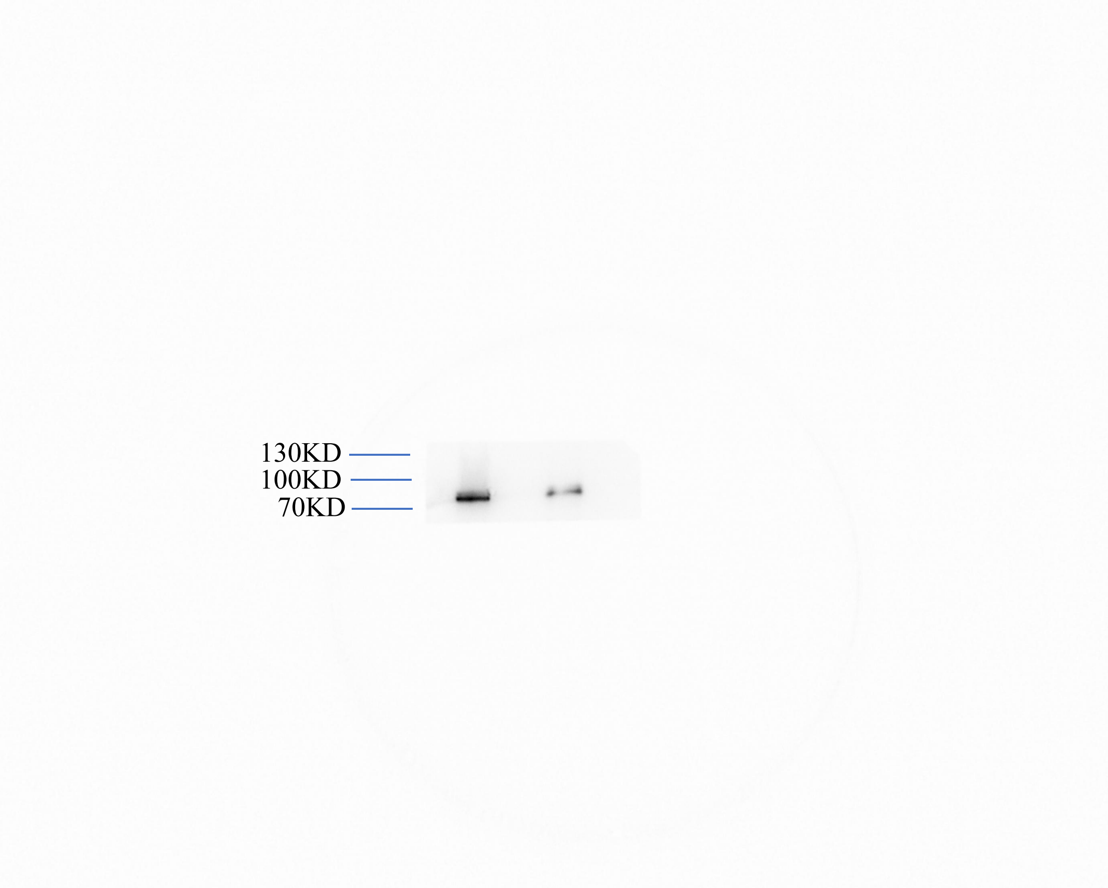

Figure4C Hep3B IP-ERK1/2 CALD1

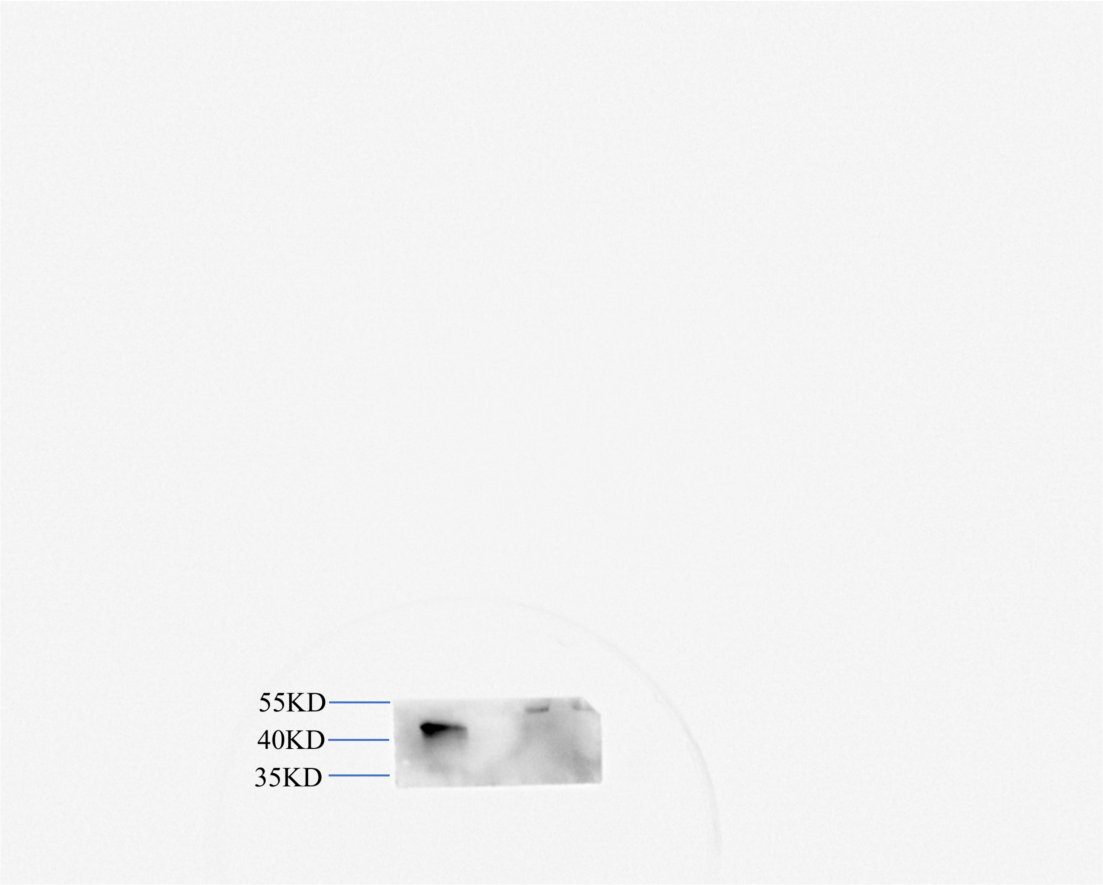

Figure4C Hep3B IP-AHSA1 ERK1/2

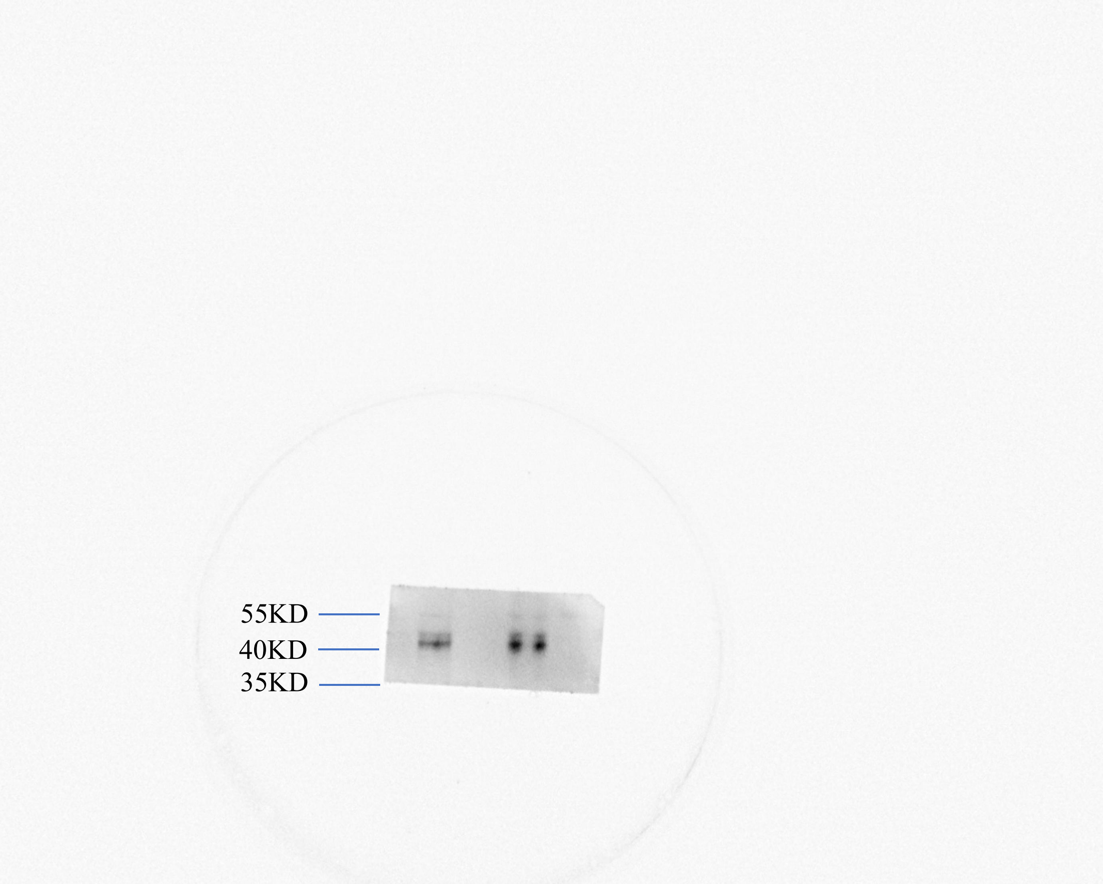

Figure4C Hep3B IP-CALD1 ERK1/2

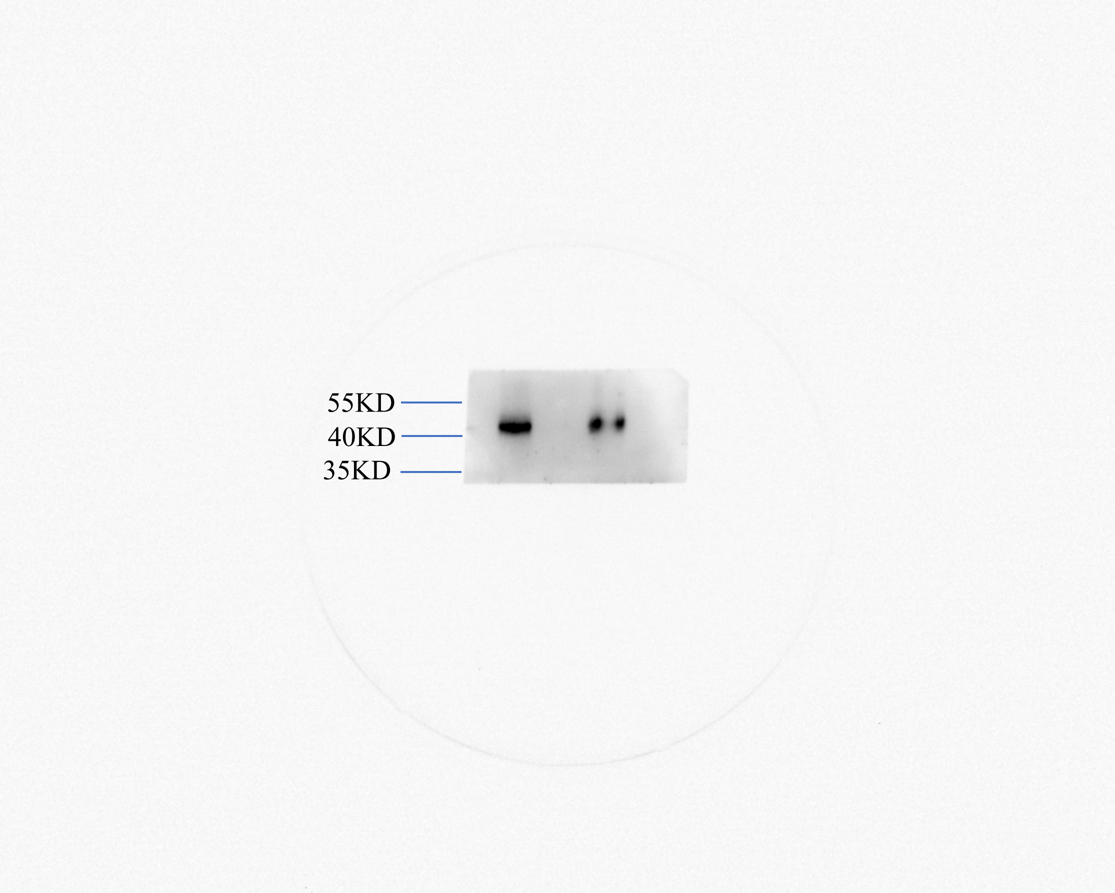

Figure4C Hep3B IP-ERK1/2 ERK1/2

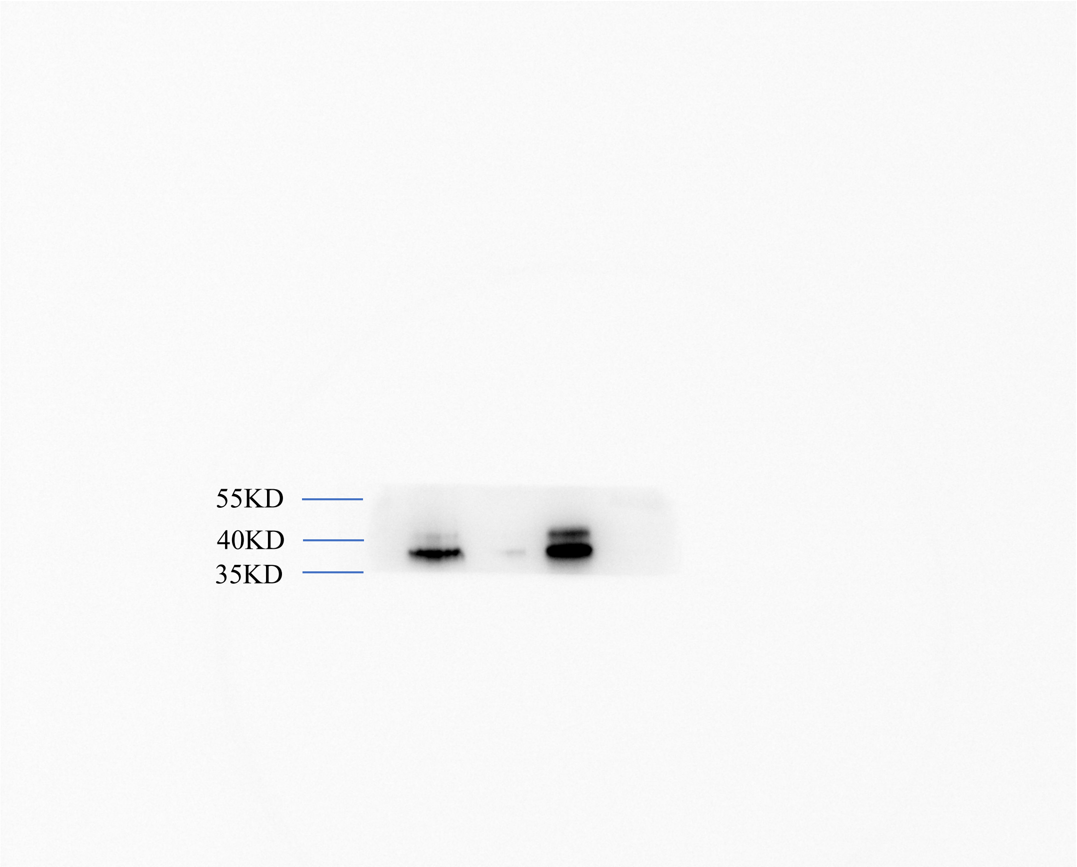

Figure4C Hep3B IP-AHSA1 AHSA1

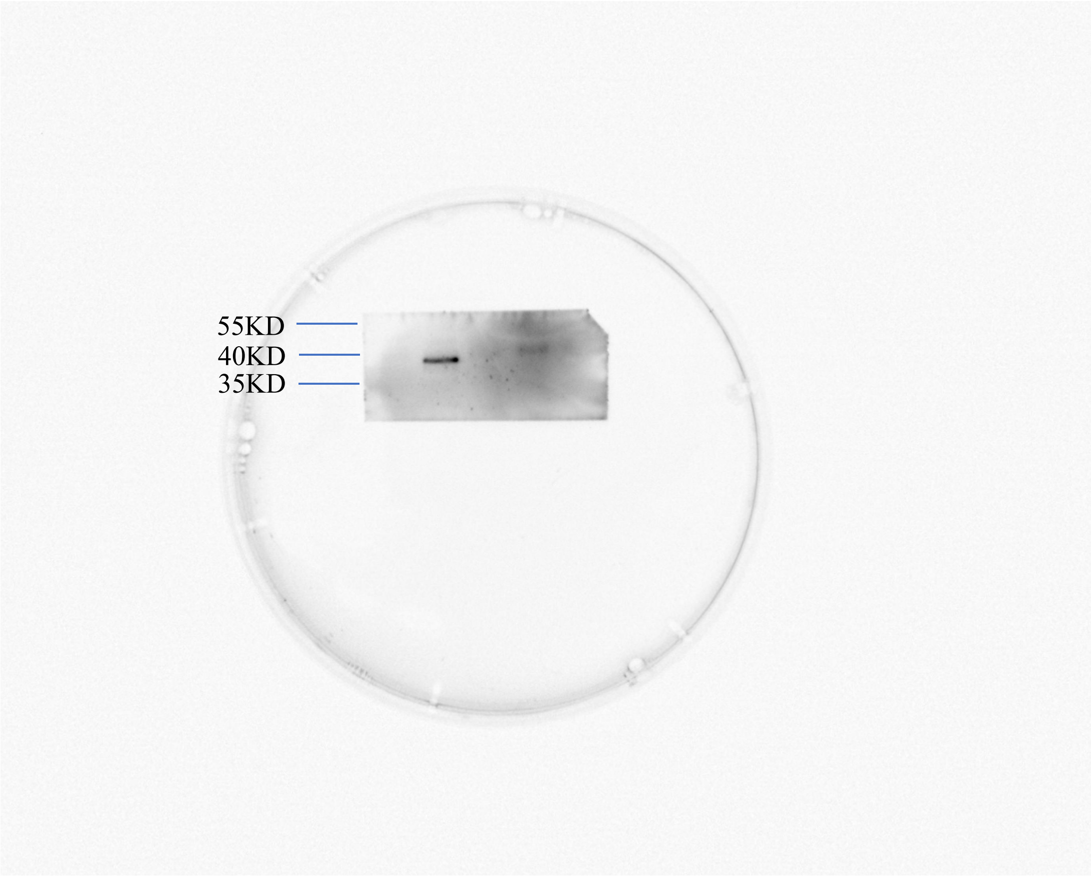

Figure4C Hep3B IP-CALD1 AHSA1

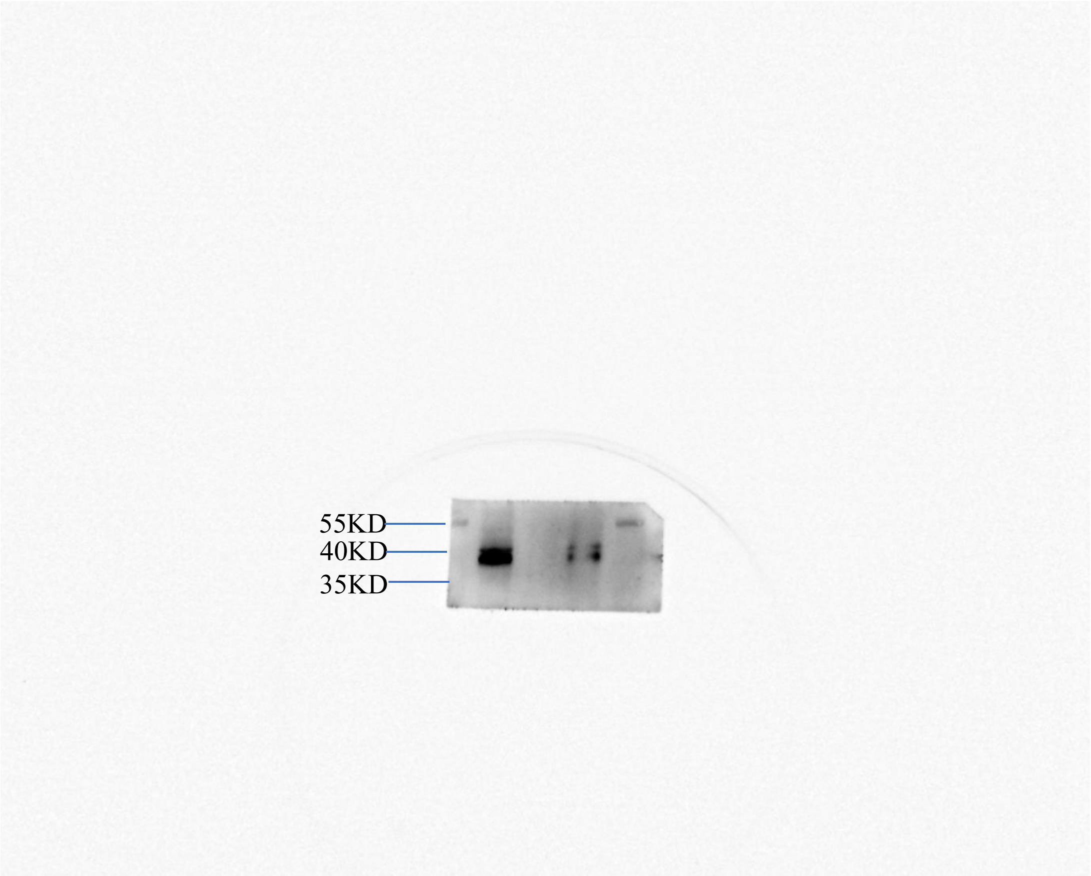

Figure4C Hep3B IP-ERK1/2 AHSA1

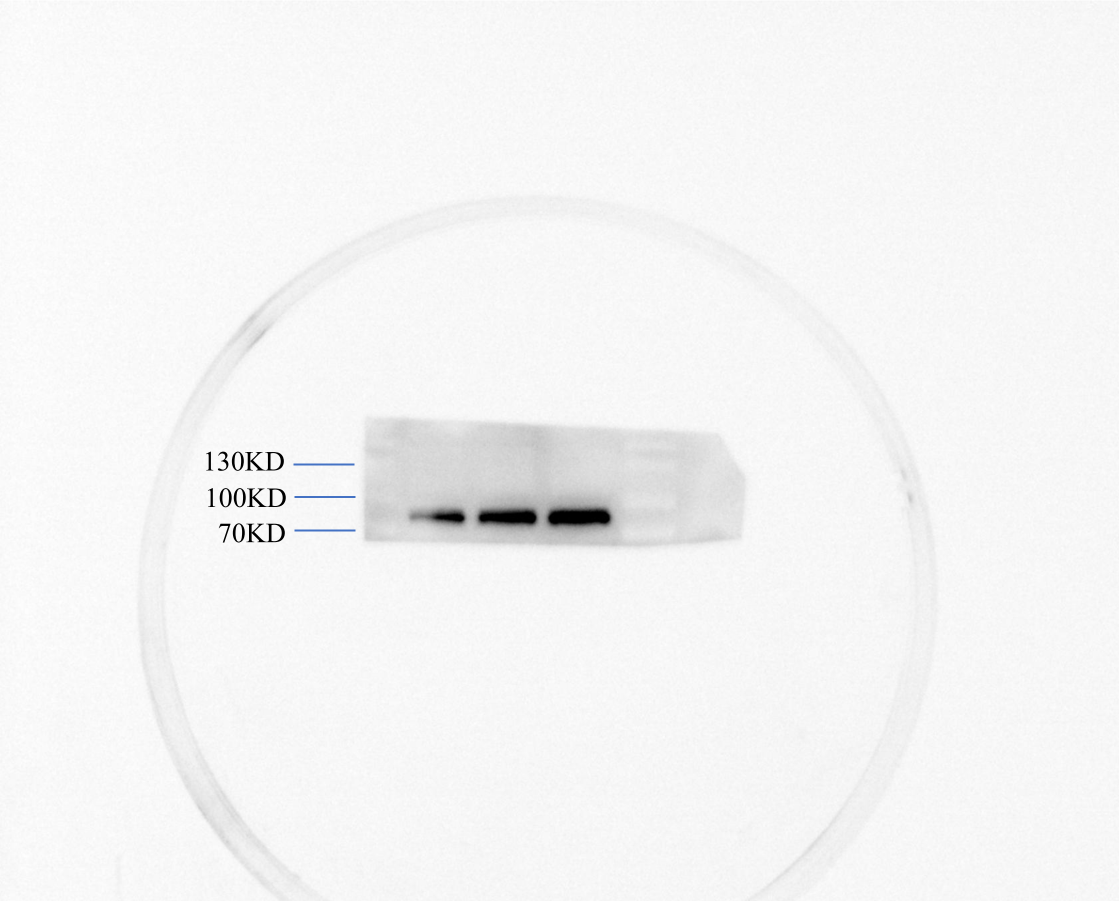

Figure4D HCCLM3 CALD1

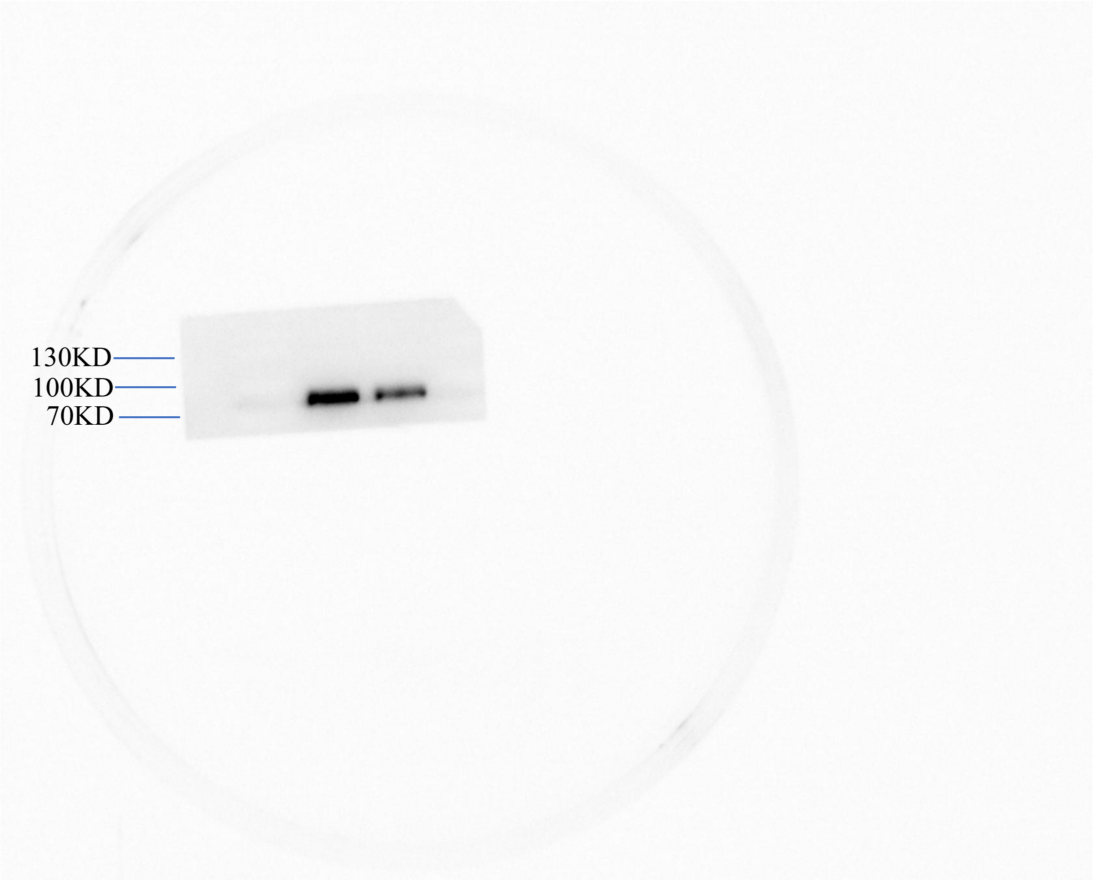

Figure4D Hep3B CALD1

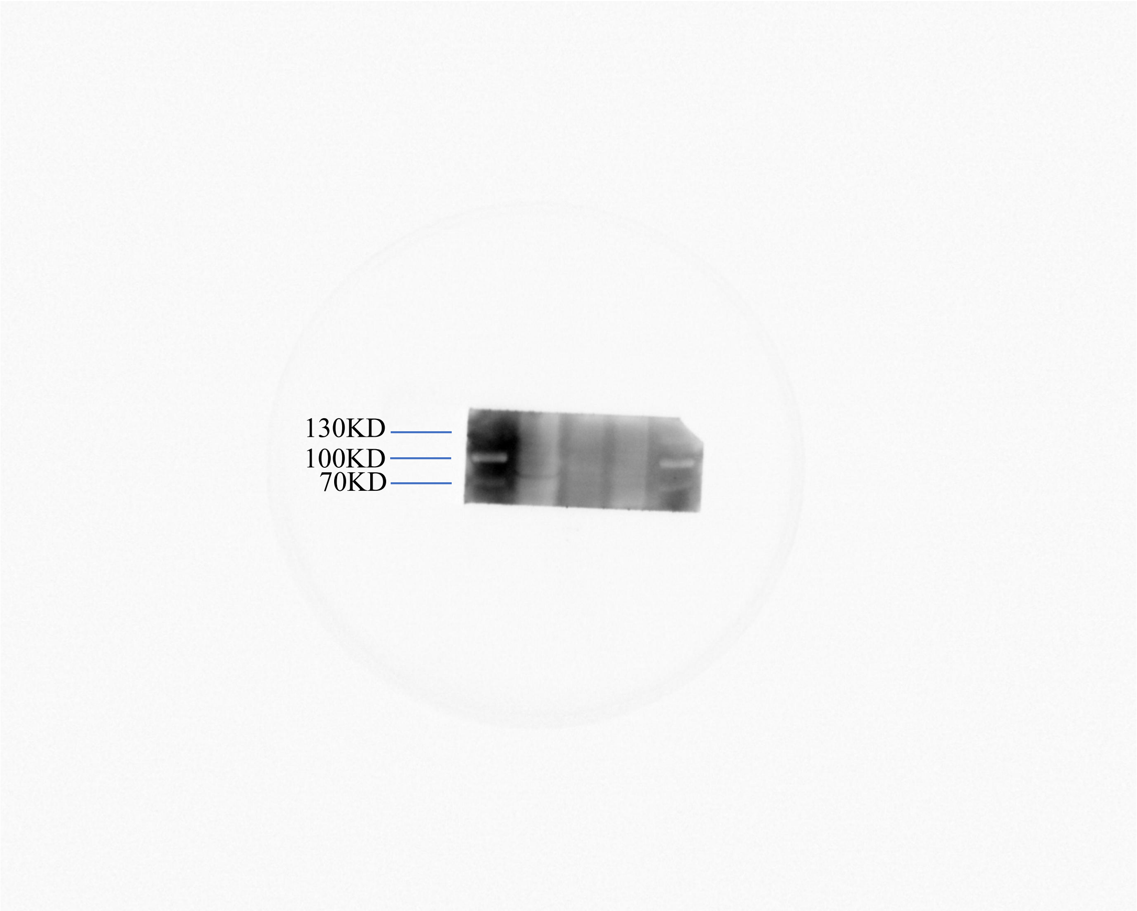

Figure4D HCCLM3 CALD1(phosphoSer759)

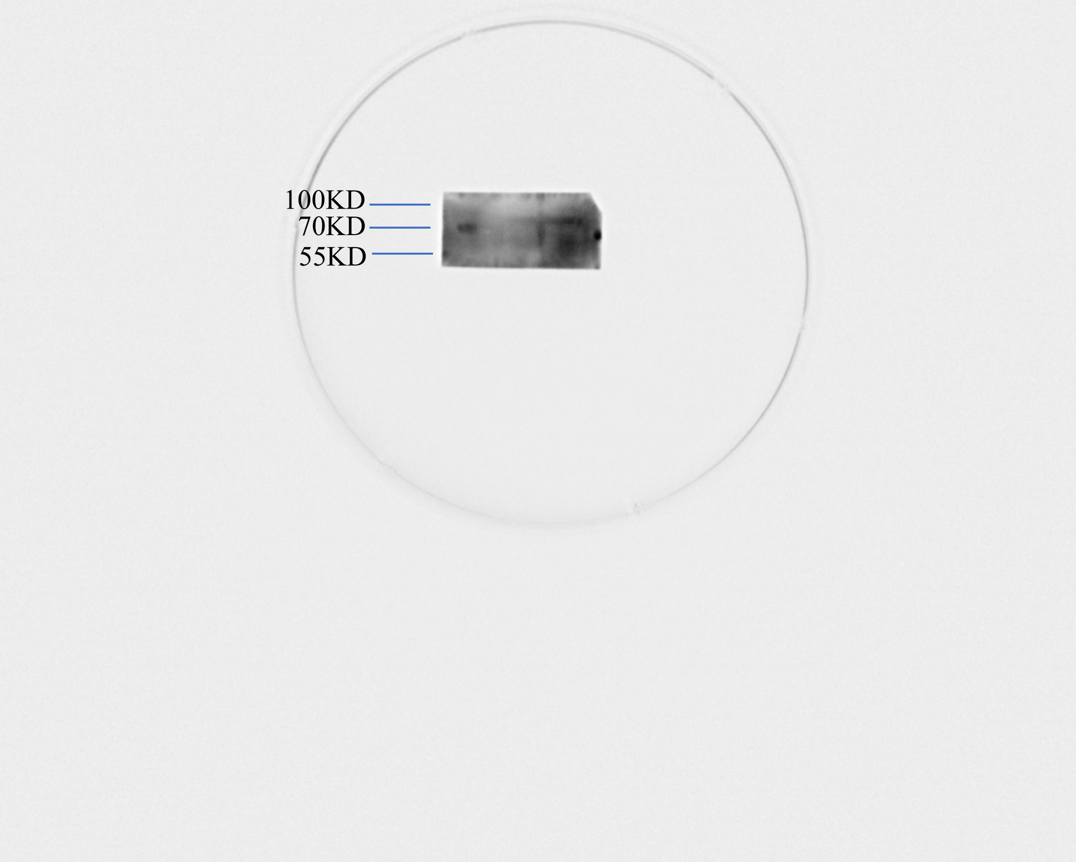

Figure4D Hep3B CALD1(phosphoSer759)

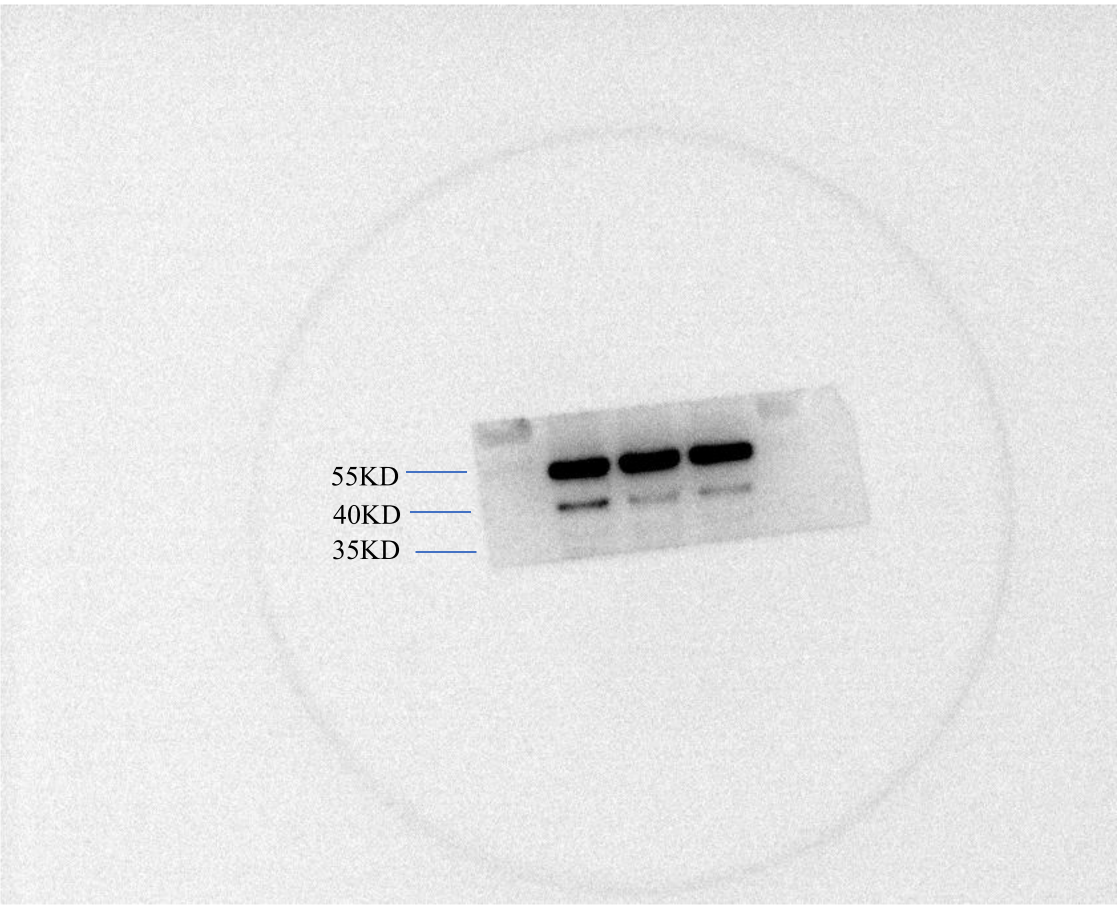

Figure4D HCCLM3 β-tubulin

Figure4D HCCLM3 AHSA1

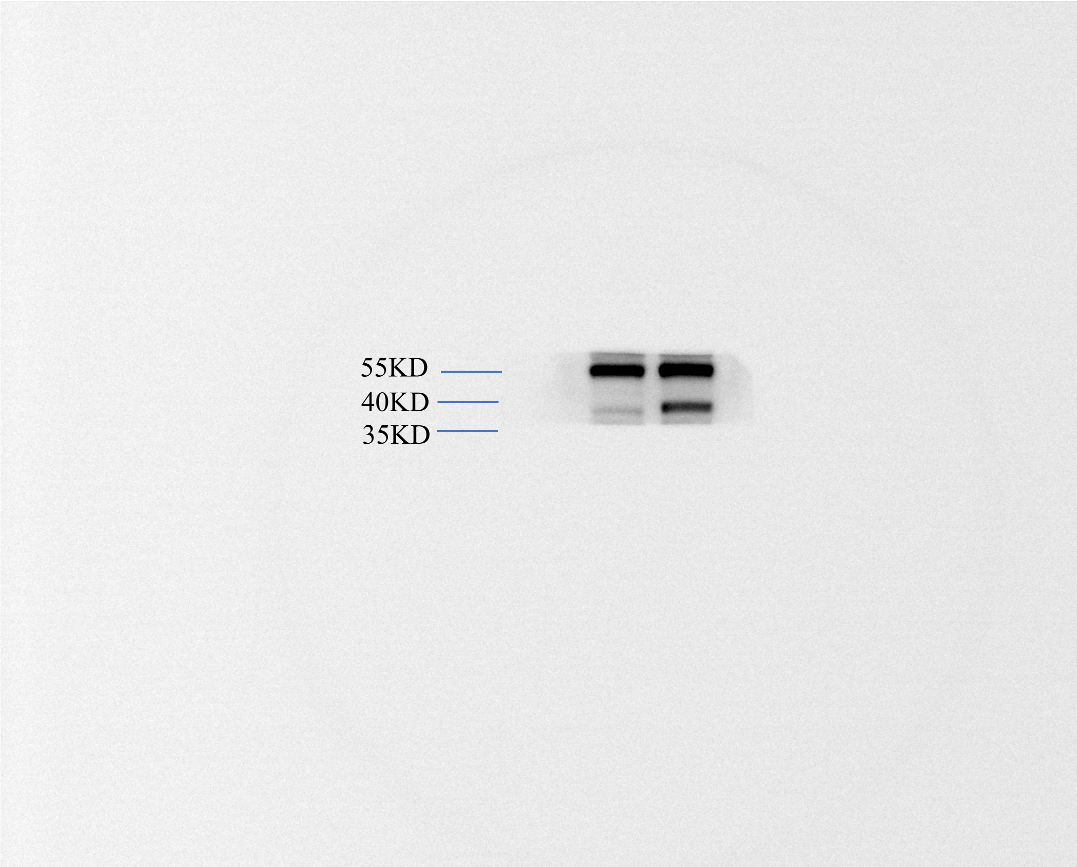

Figure4D Hep3B β-tubulin

Figure4D Hep3B AHSA1

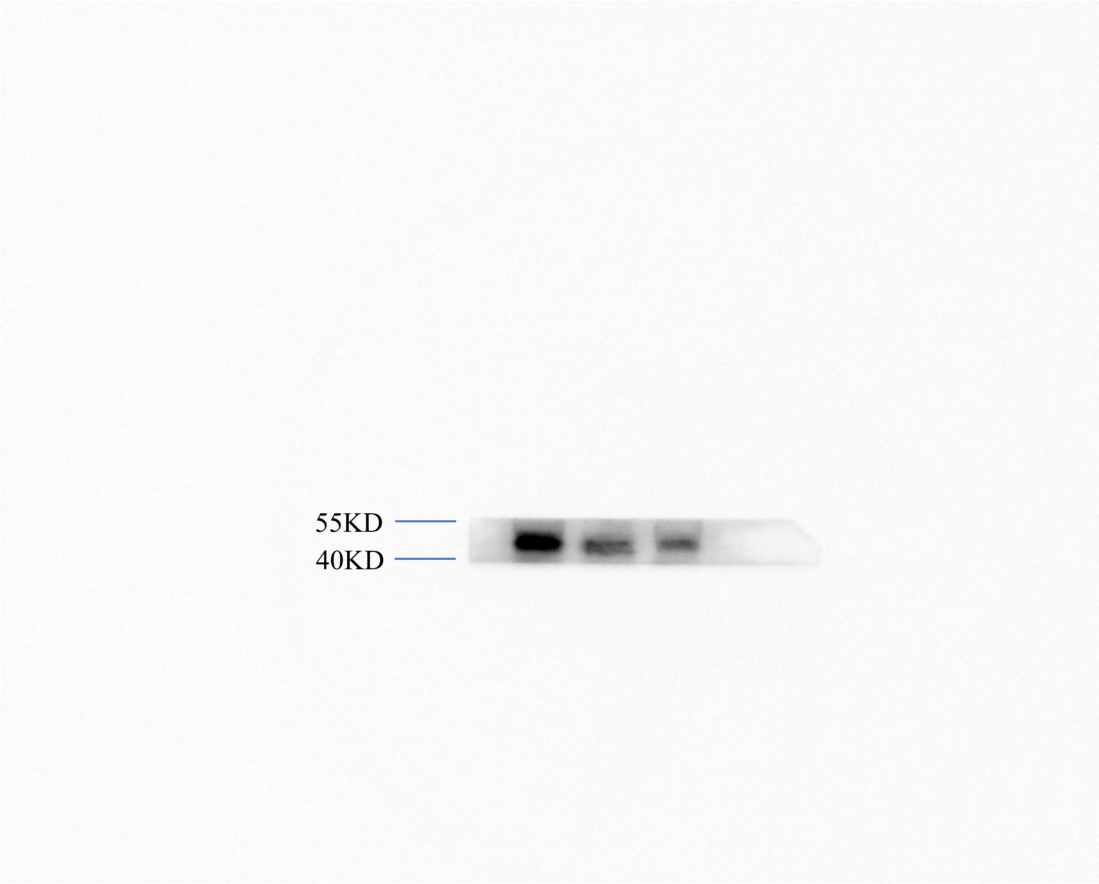

Figure4D　HCCLM3　ERK1/2

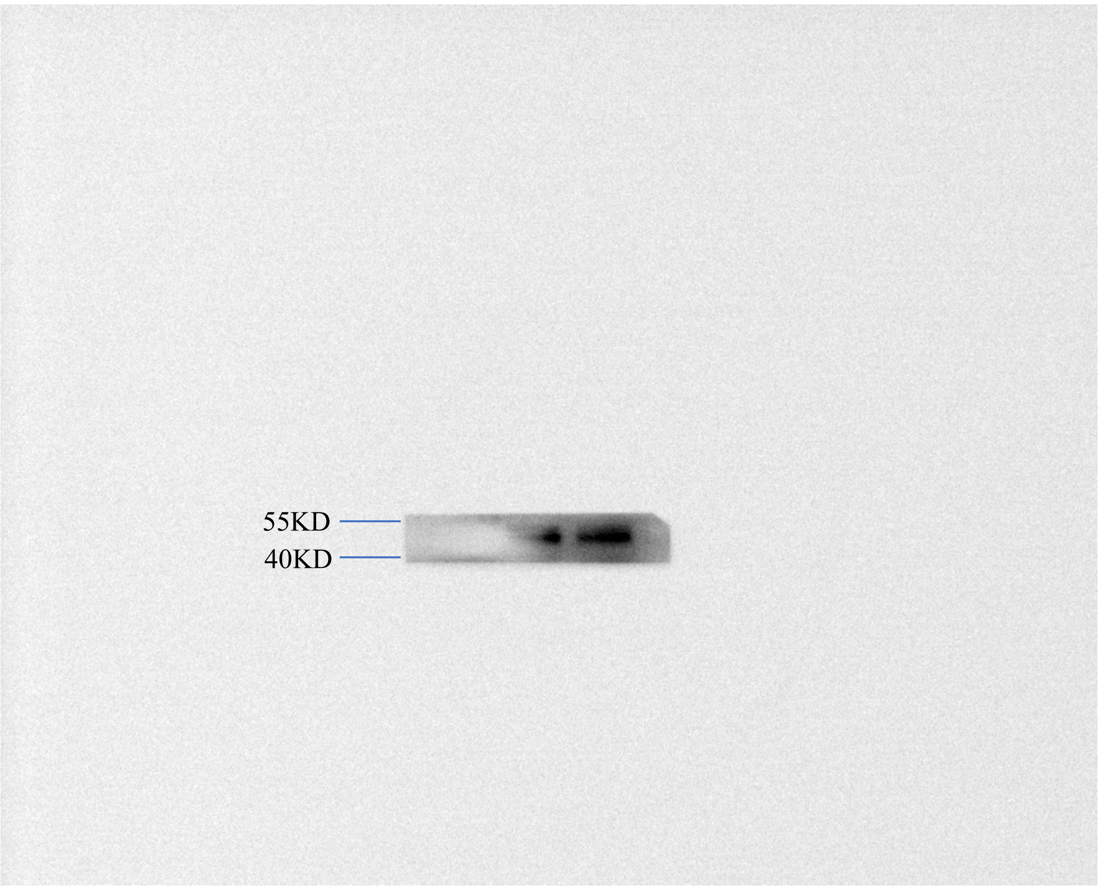

Figure4D Hep3B ERK1/2

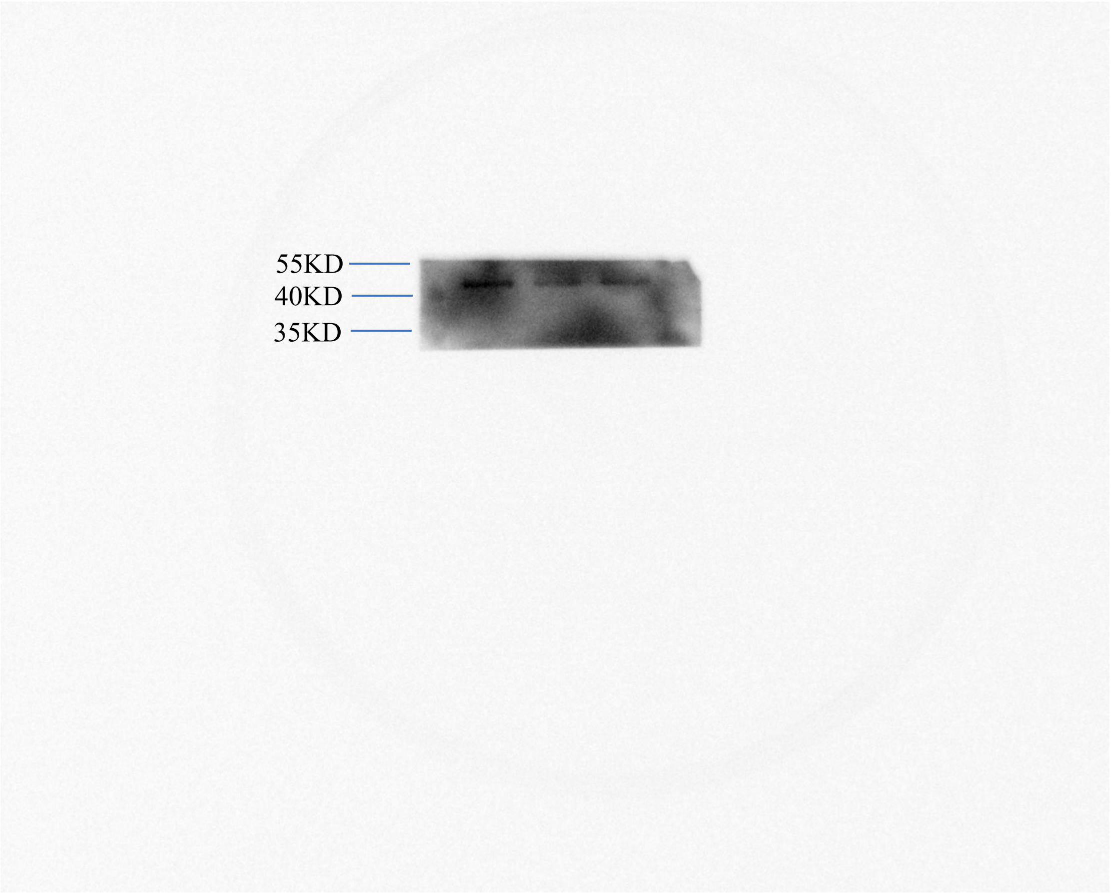

Figure4D HCCLM3 ERK1/2(phosphoThr202/Tyr204)

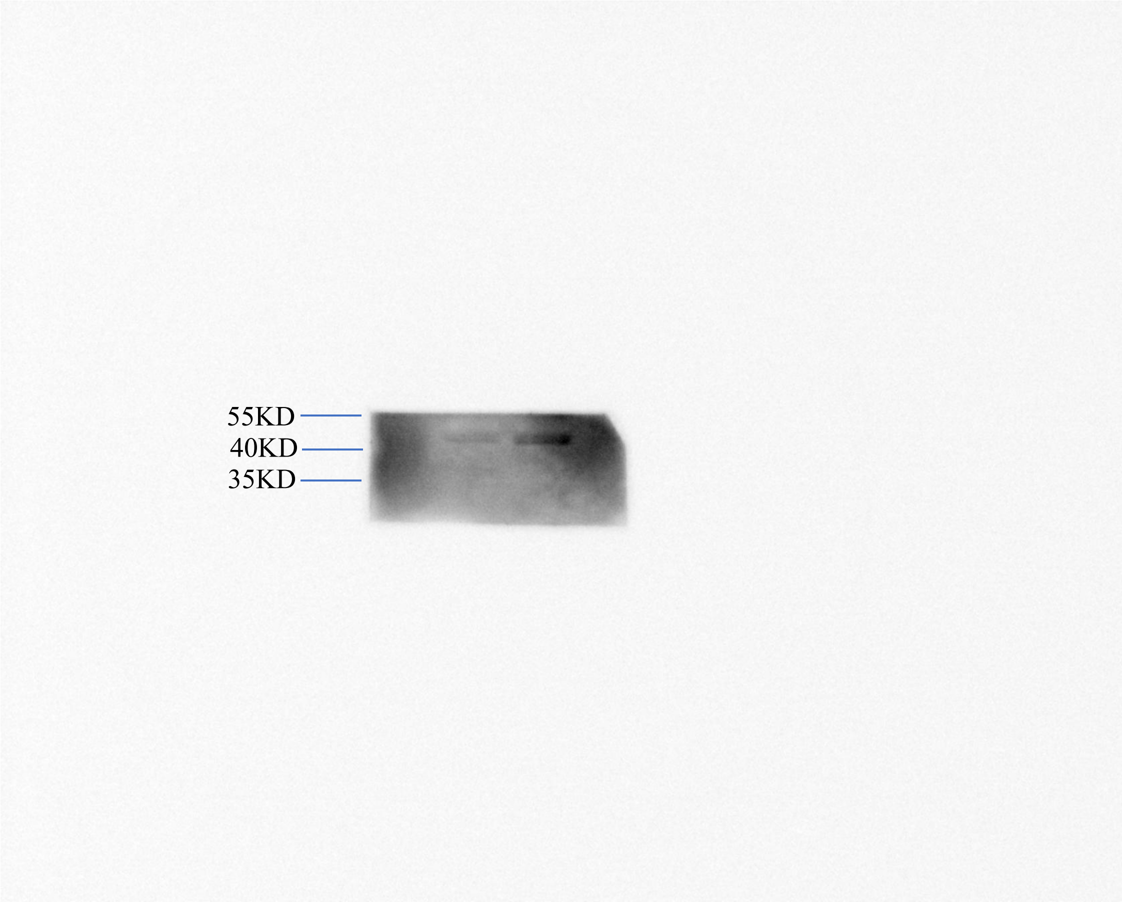

Figure4D Hep3B ERK1/2(phosphoThr202/Tyr204)

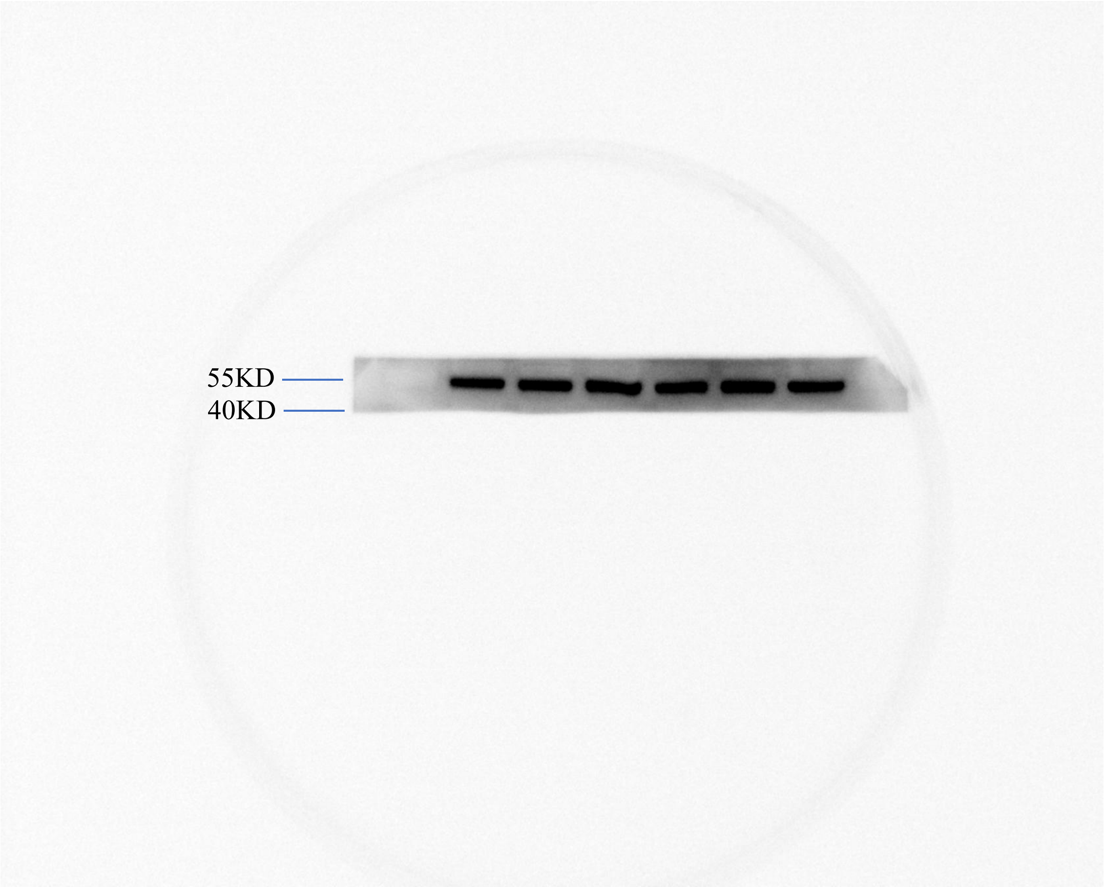

Figure4E β-tubulin

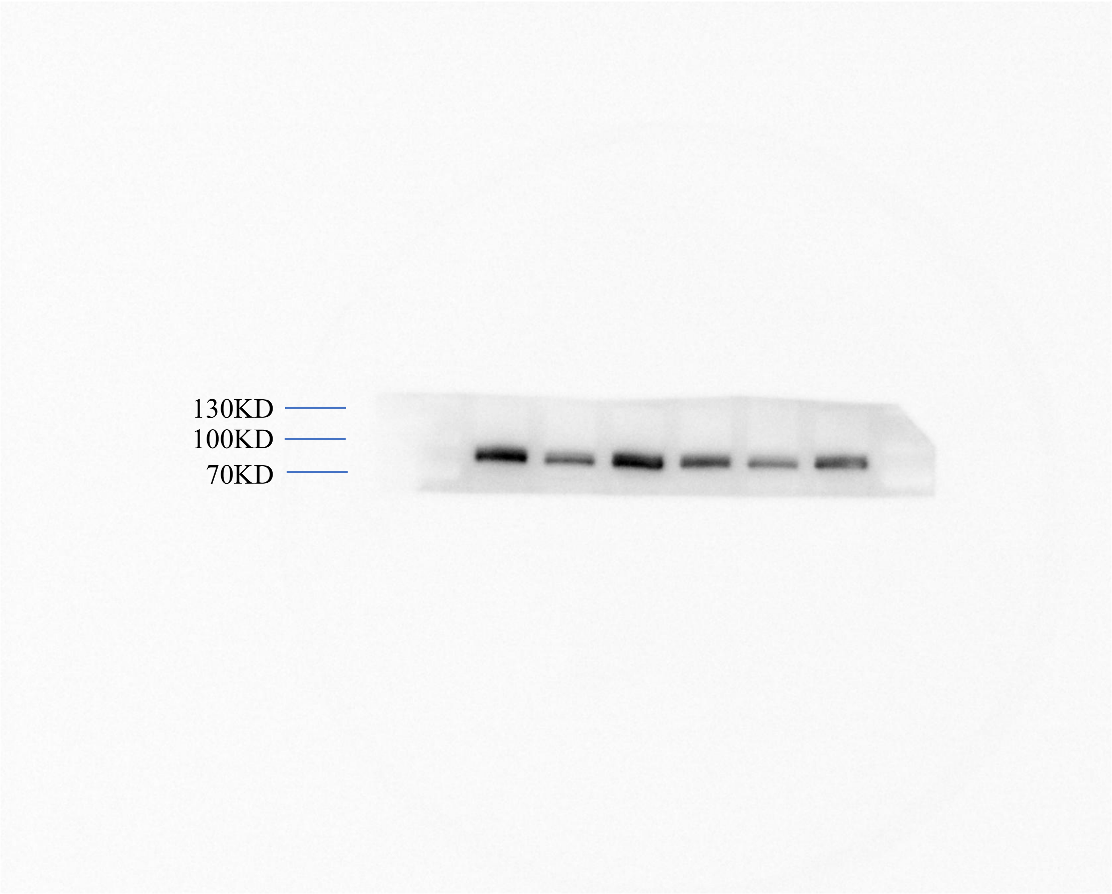

Figure4E CALD1

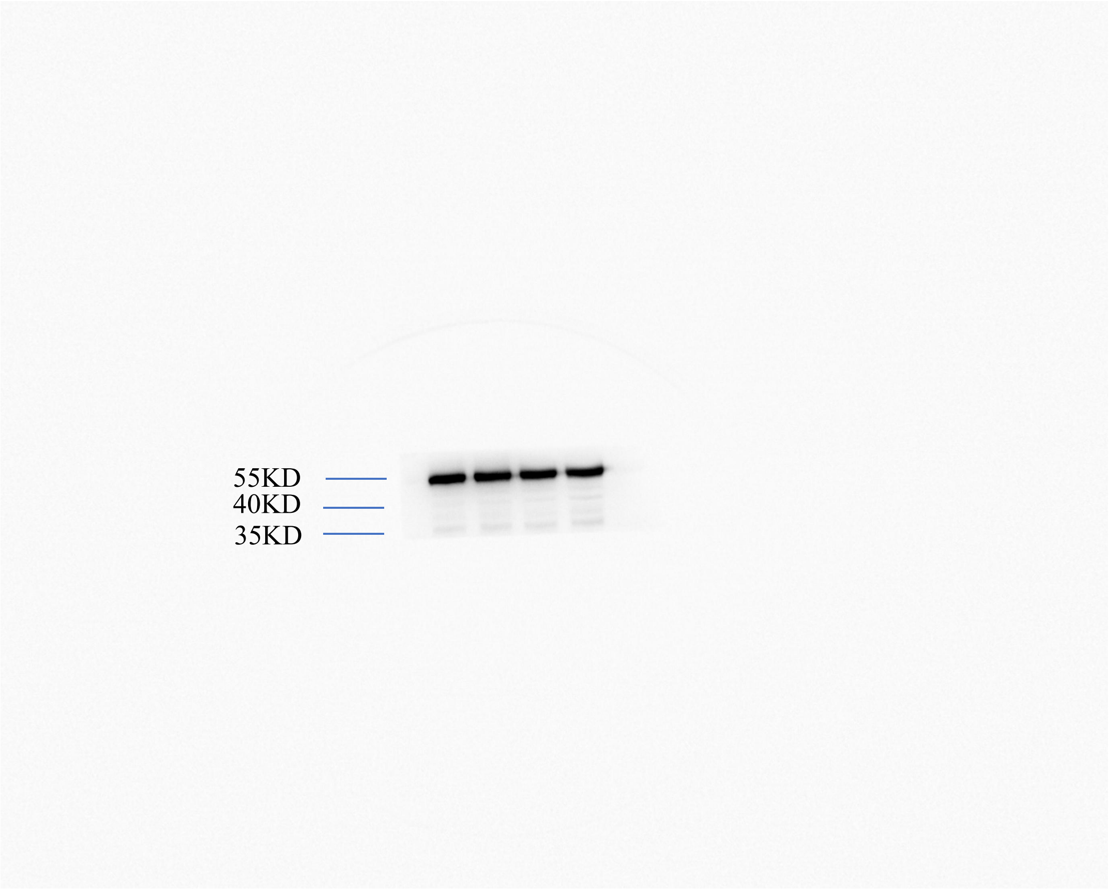

Figure5A   β-tubulin

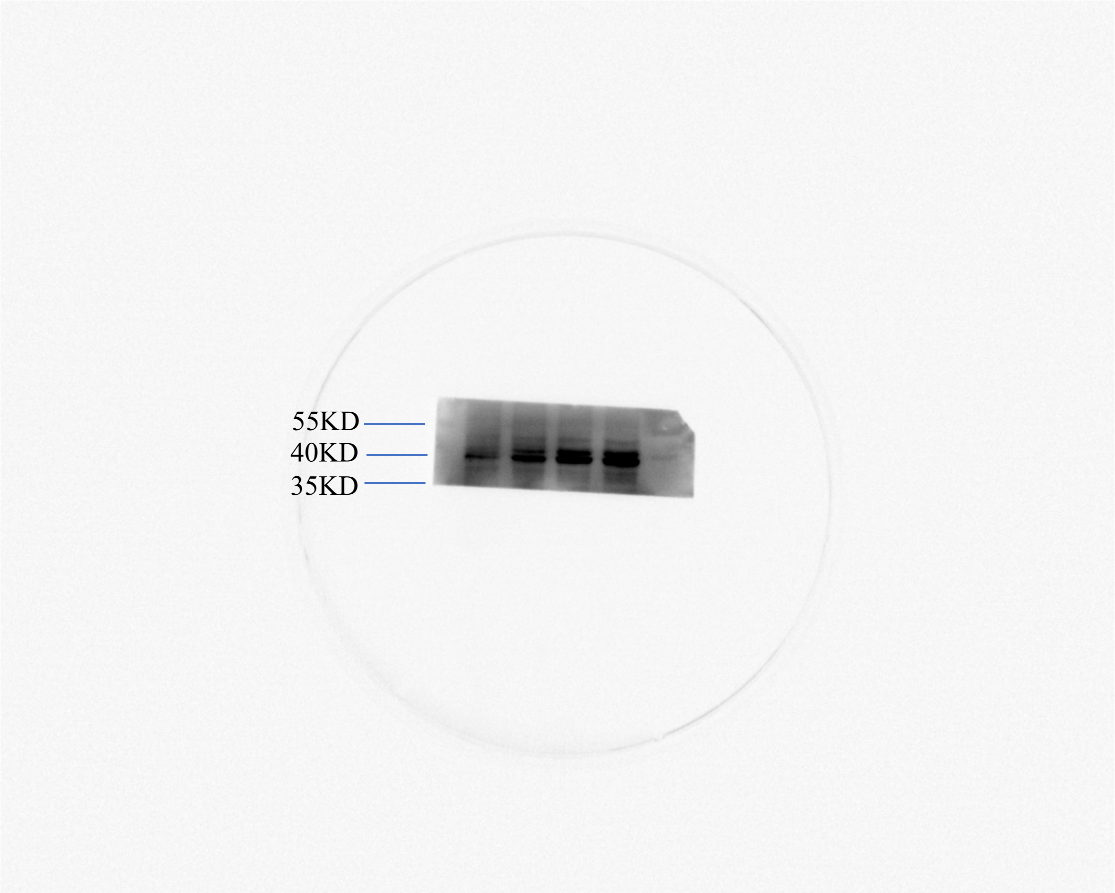

Figure5A ERK1/2

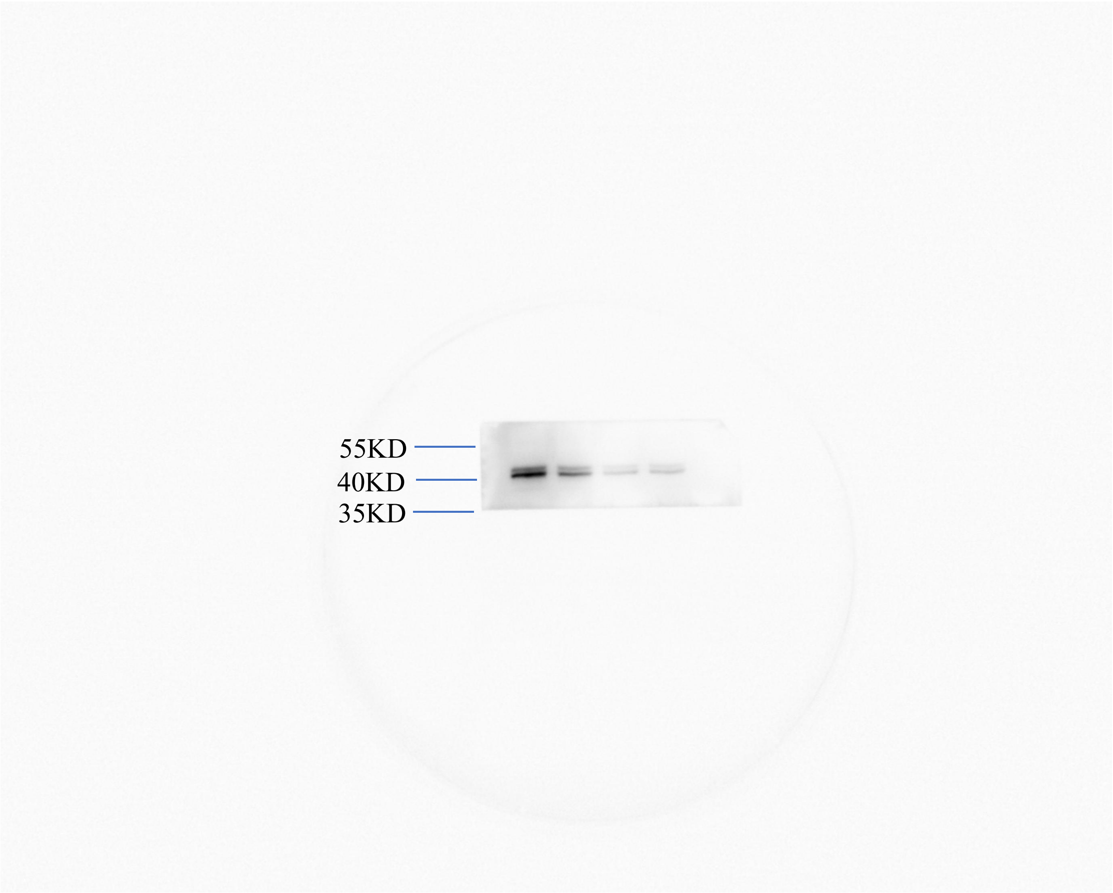

Figure5A Hep3B ERK1/2(phosphoThr202/Tyr204)

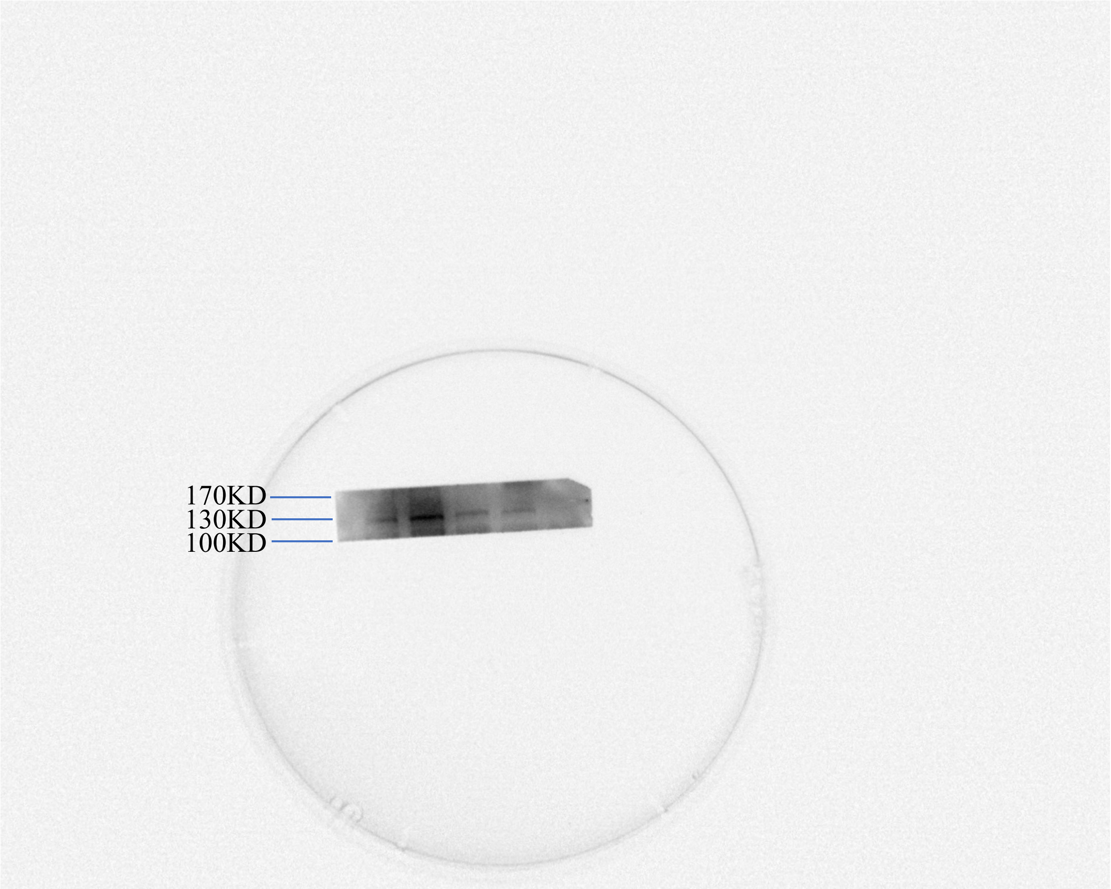

Figure5H Hep3B N-cadherin

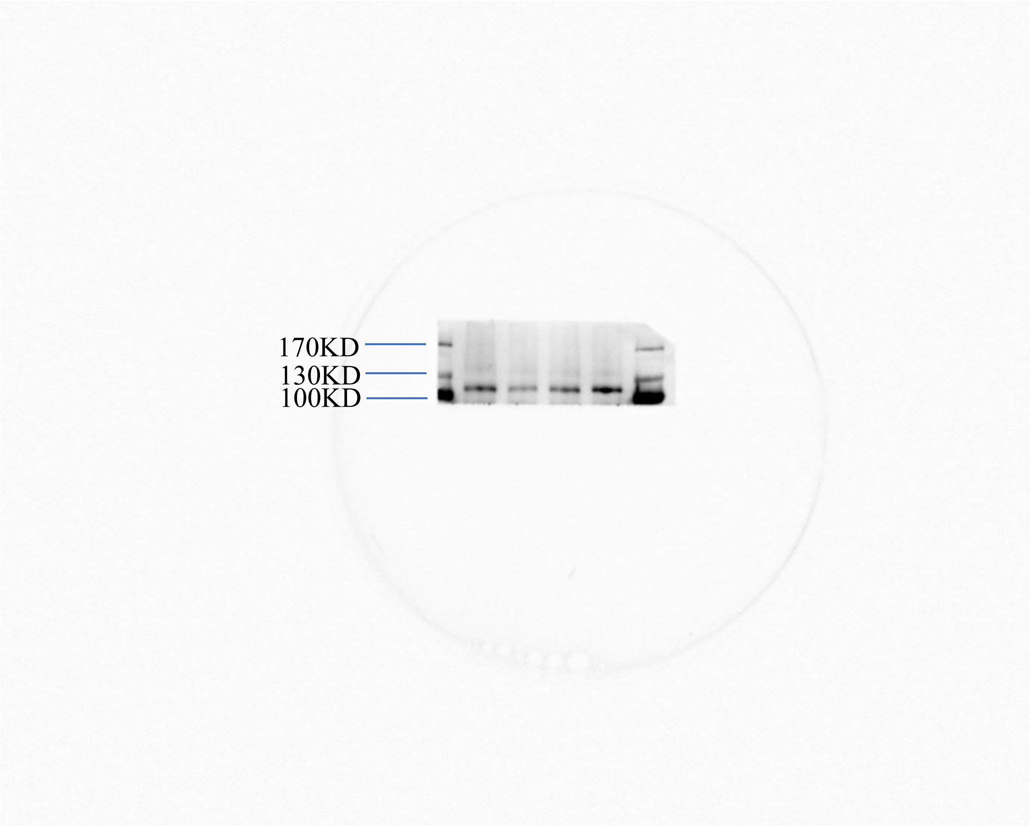

Figure5H Hep3B E-cadherin

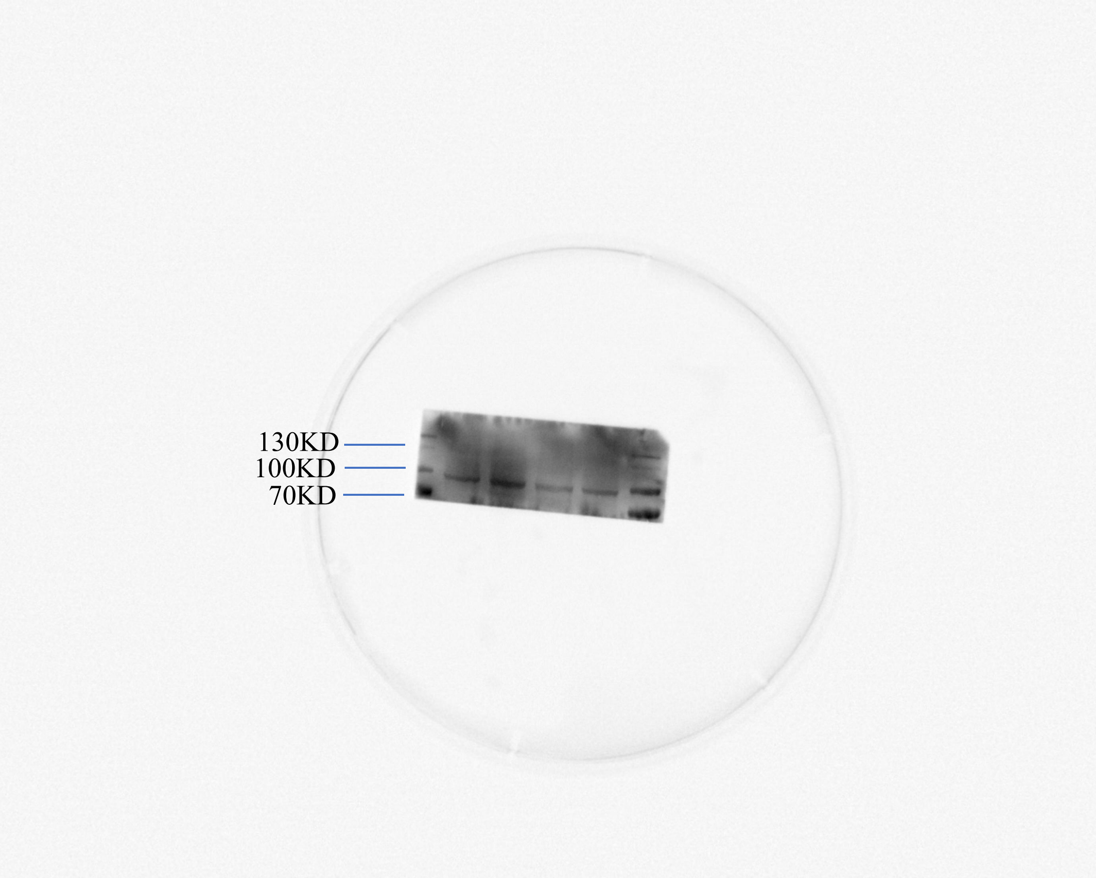

Figure5H Hep3B CALD1(phosphoSer759)

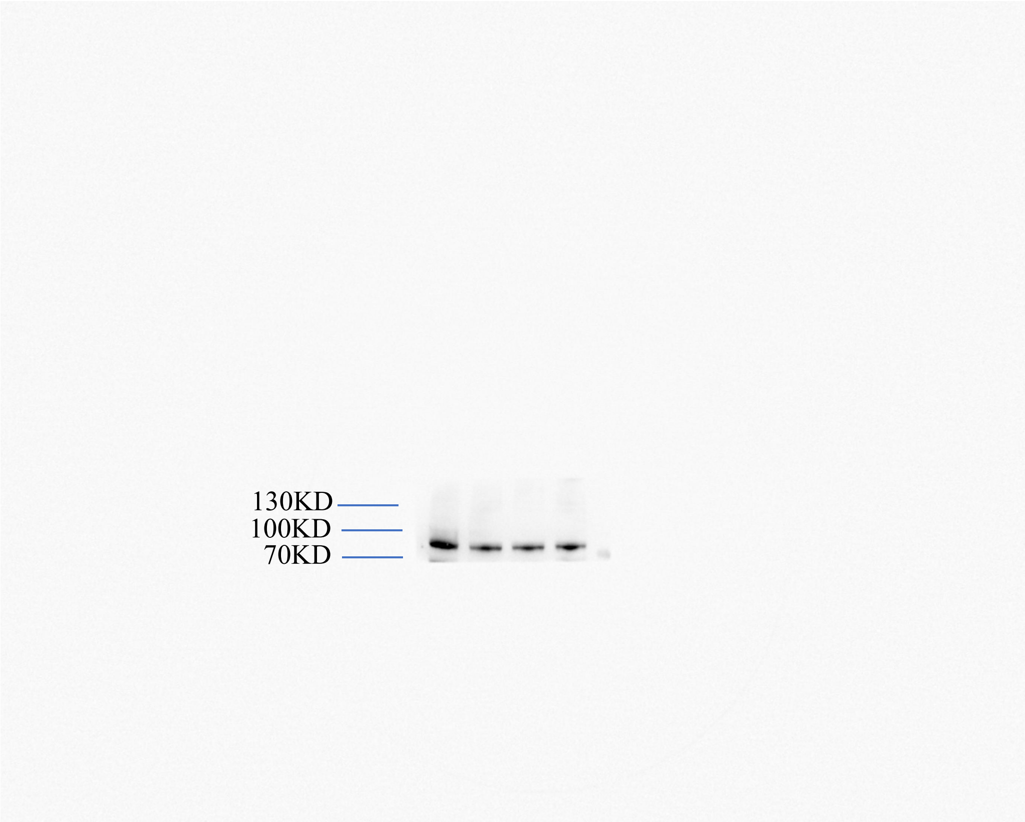

Figure5H Hep3B CALD1

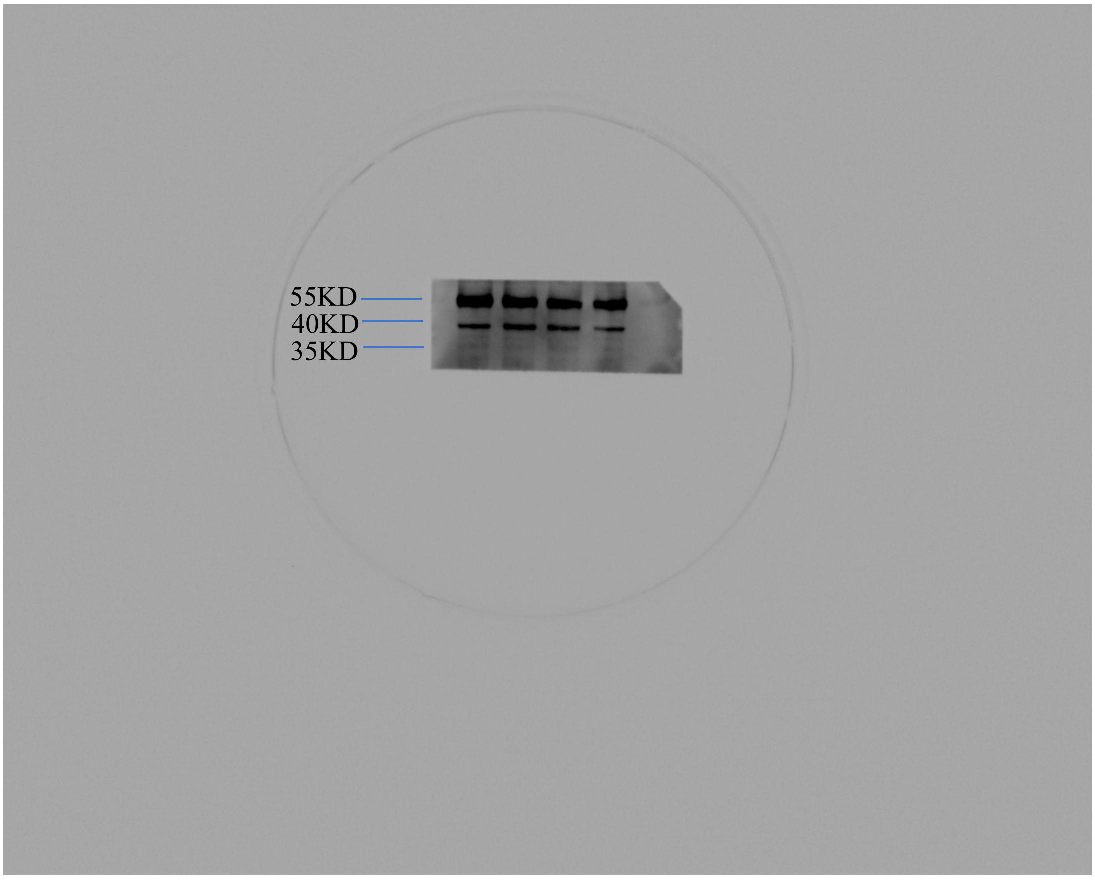

Figure5H Hep3B β-tubulin

Figure5H Hep3B AHSA1

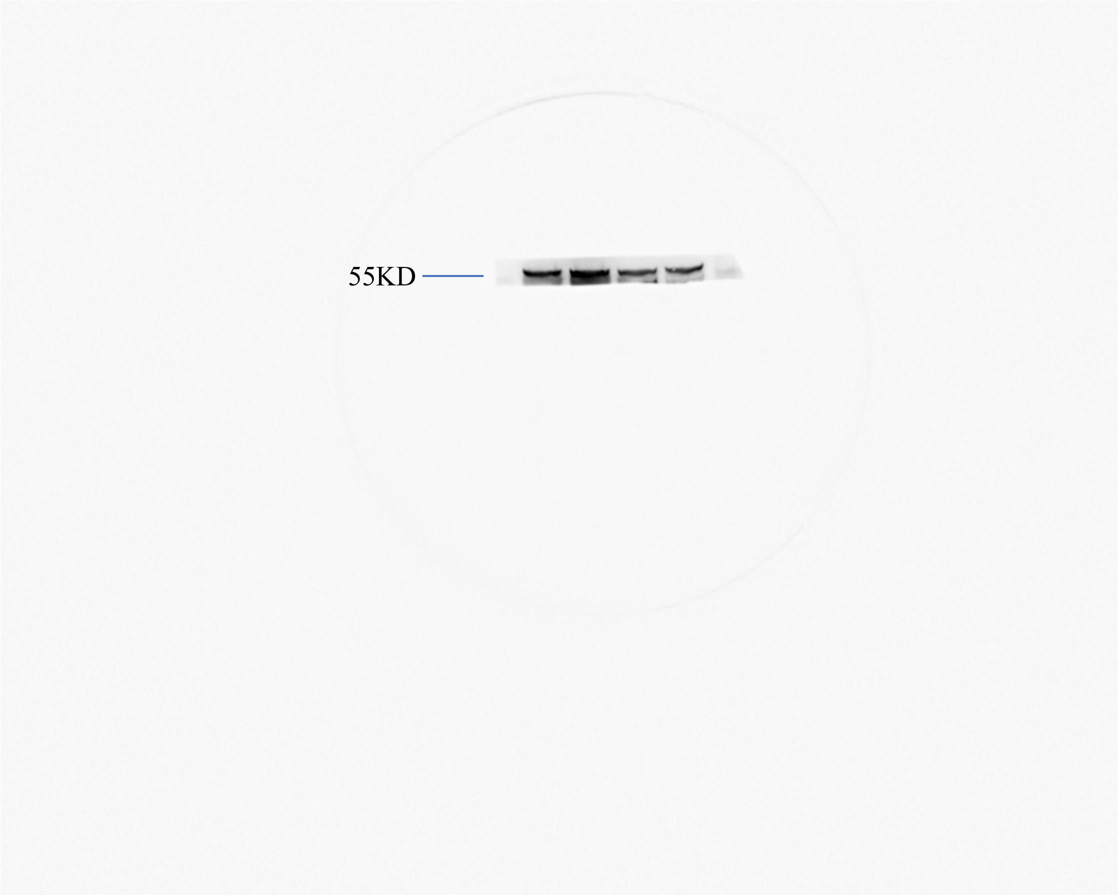

Figure5H Hep3B vimentin

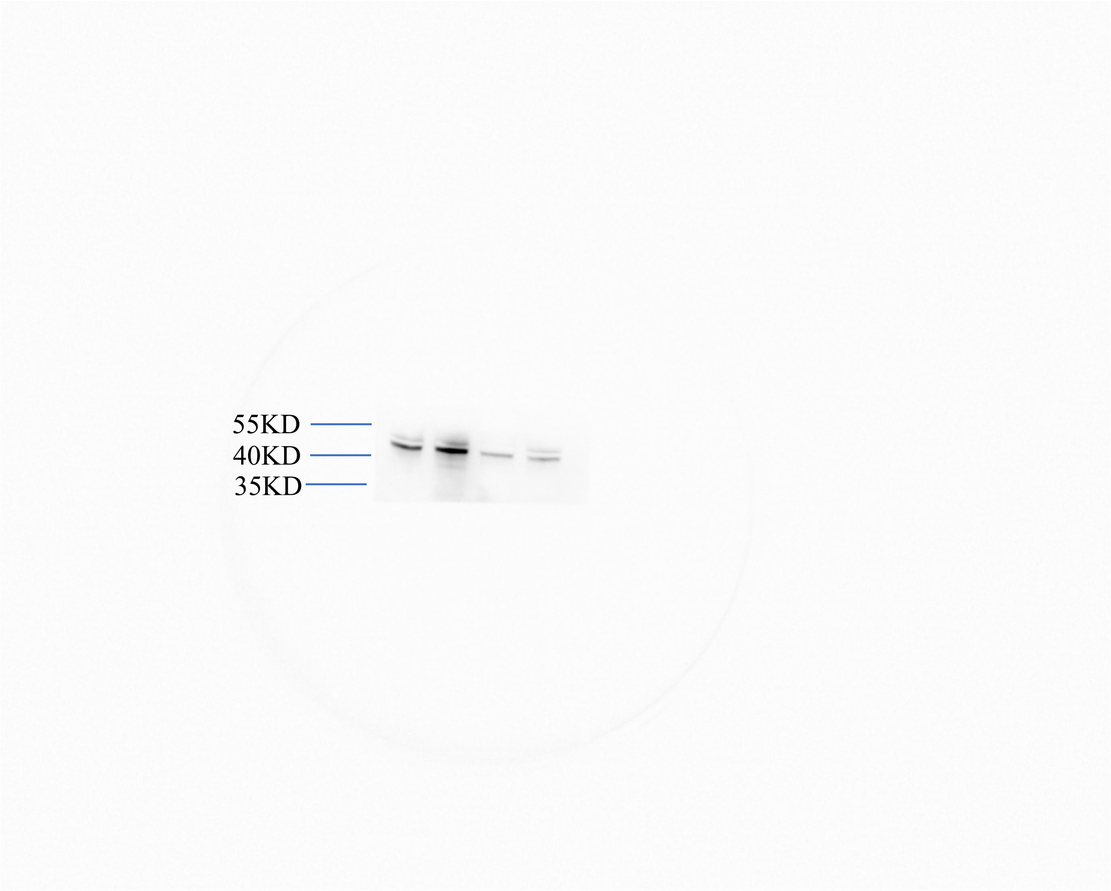

Figure5H Hep3B ERK1/2(phosphoThr202/Tyr204)

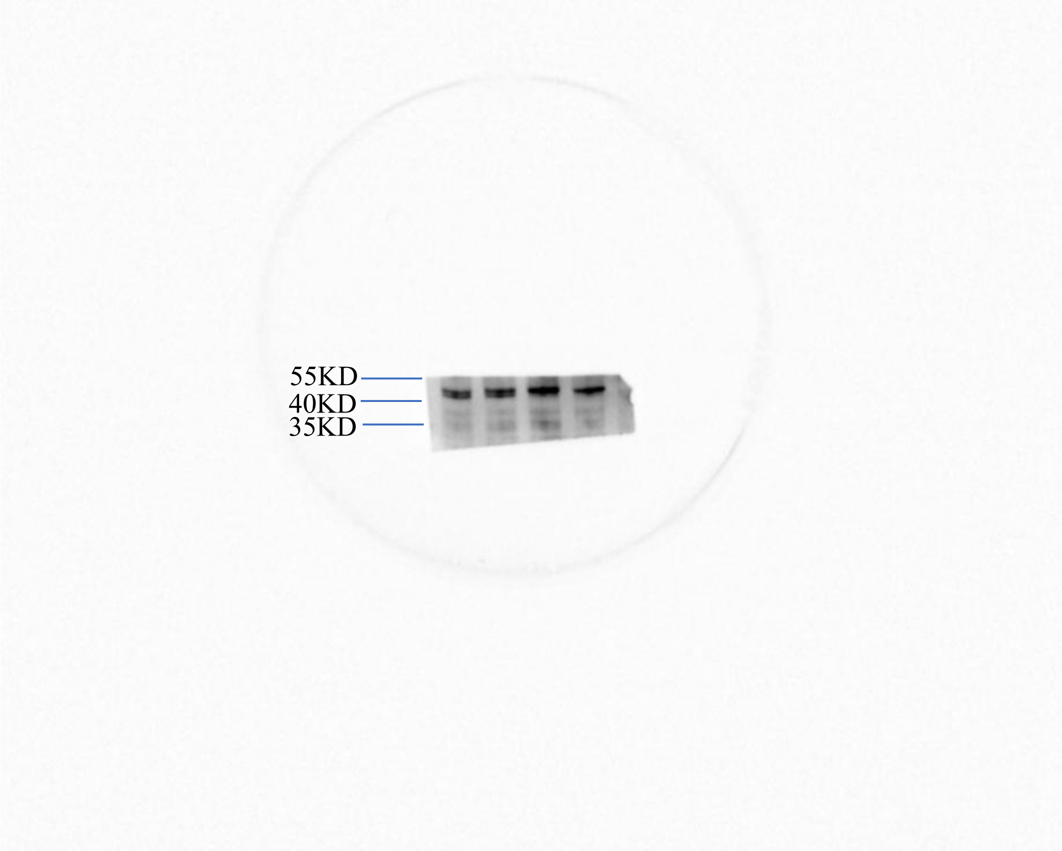

Figure5H Hep3B ERK1/2

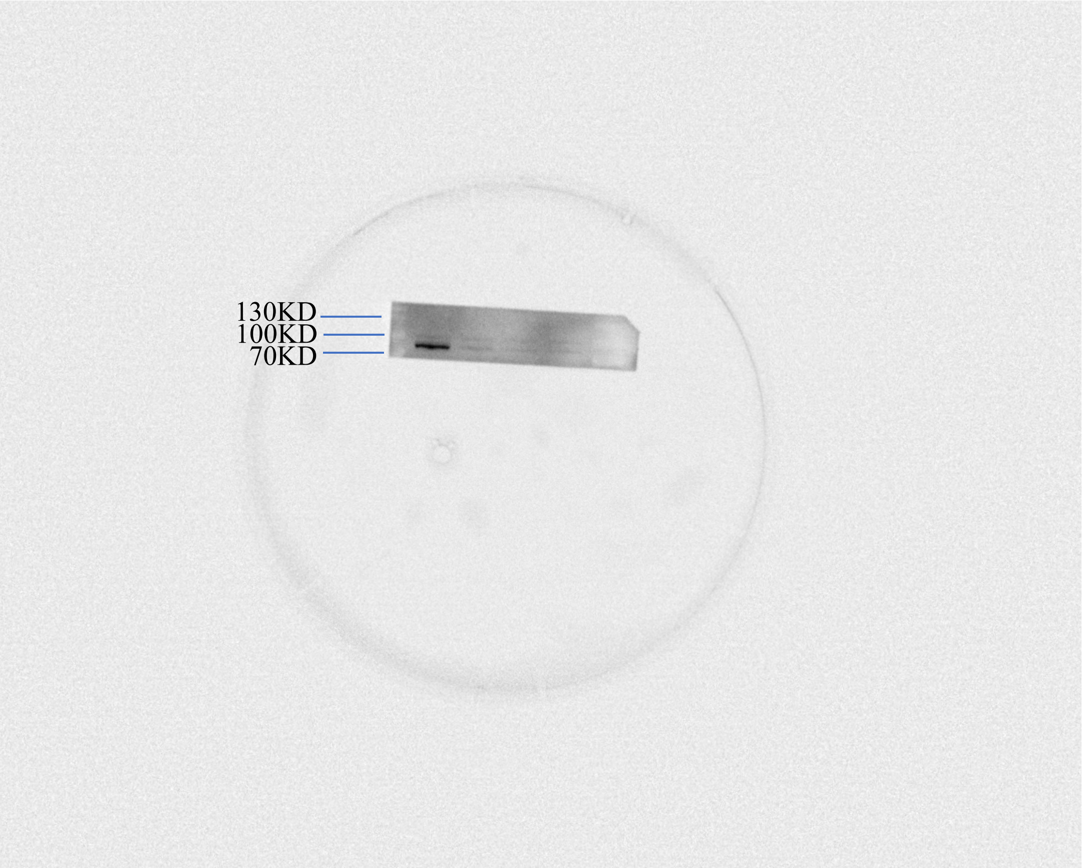

Figure6A HCCLM3 CALD1

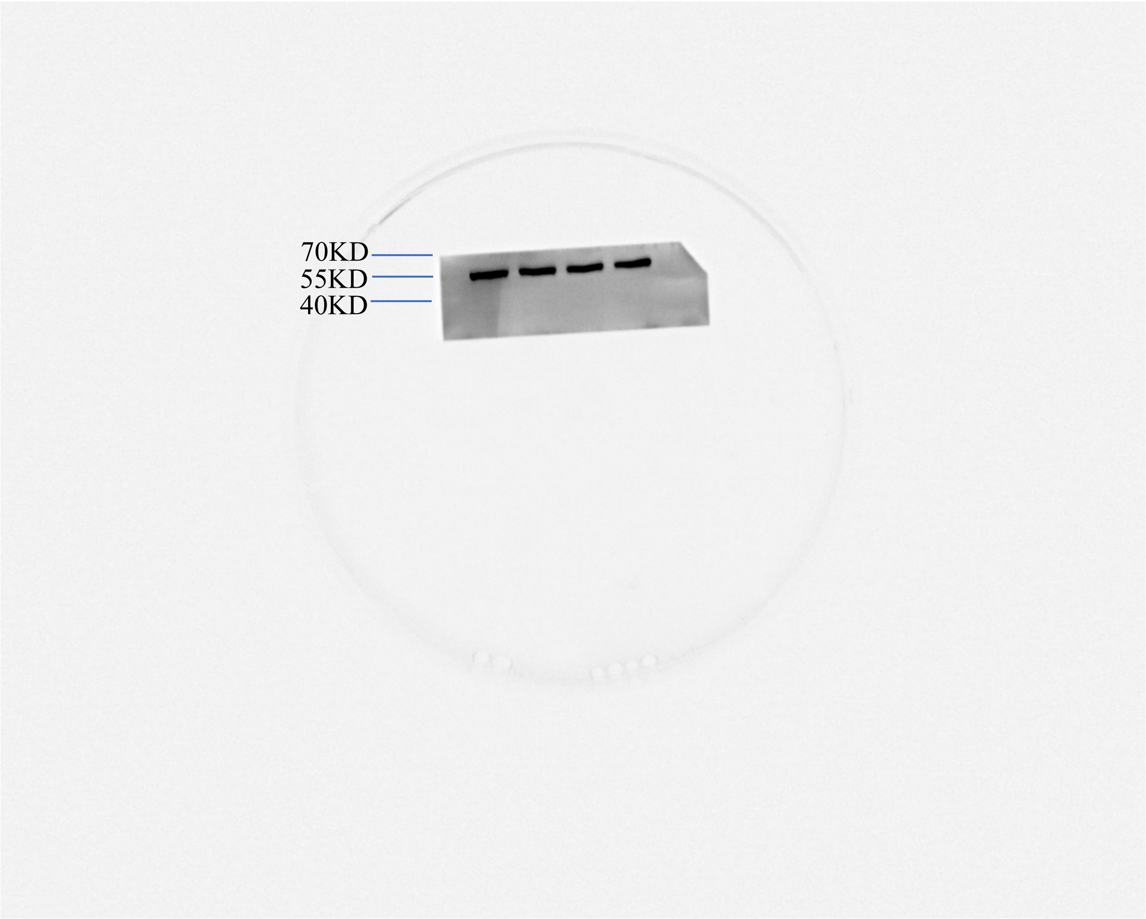

Figure6A　HCCLM3　β-tubulin

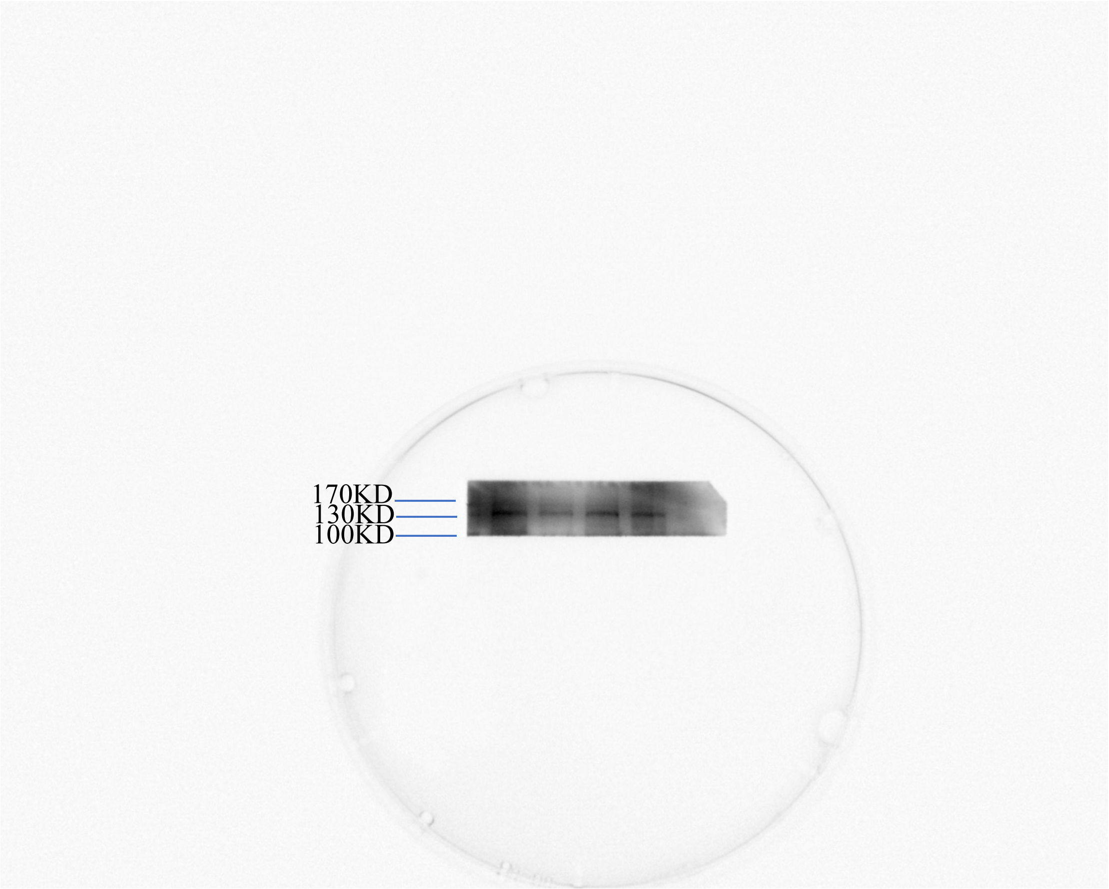

Figure6H   HCCLM3 N-cadherin

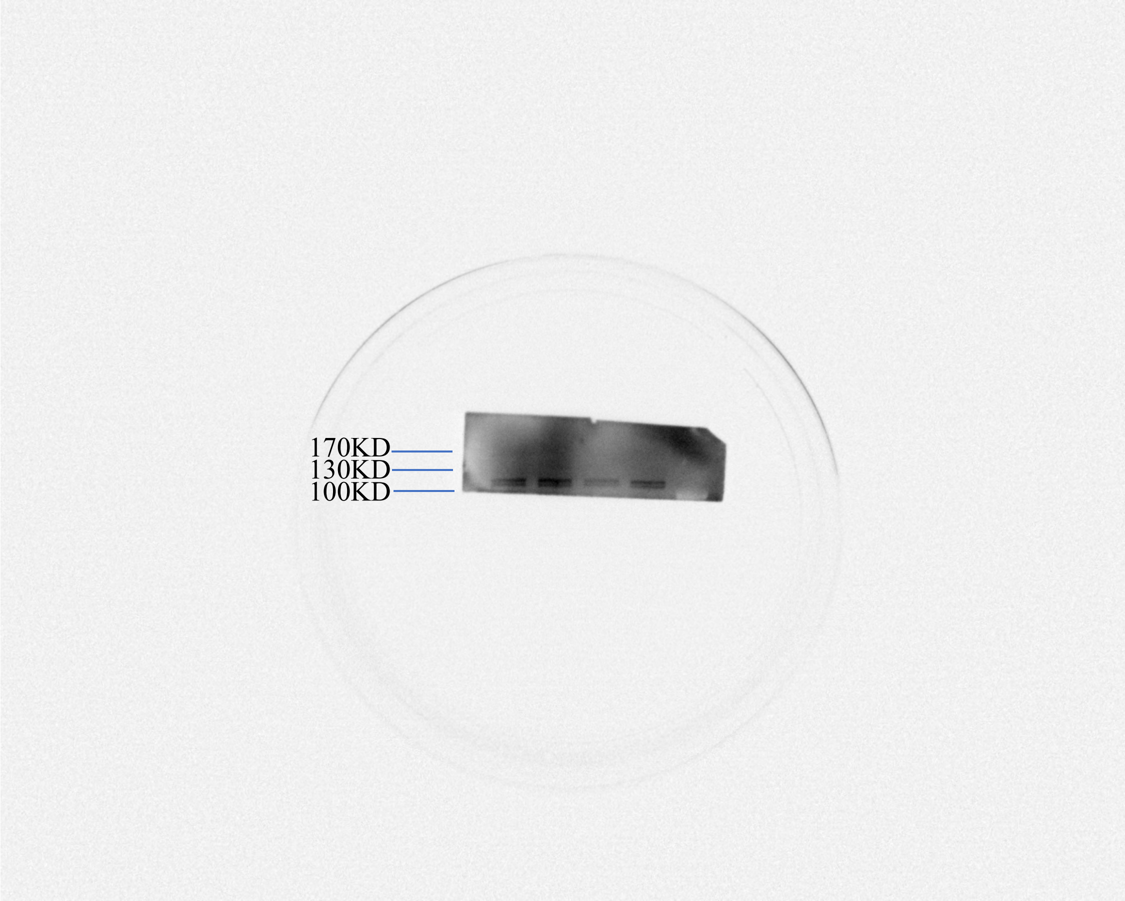

Figure6H HCCLM3 E-cadherin

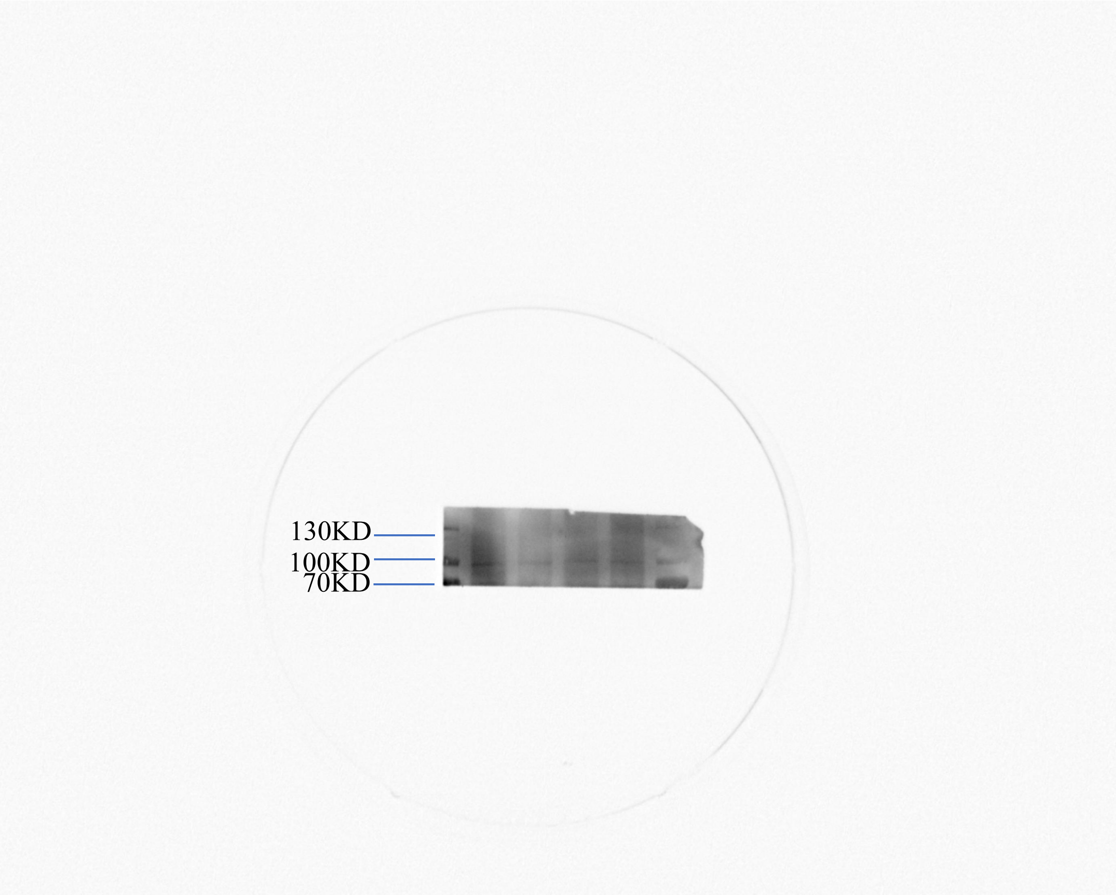

Figure6H HCCLM3 CALD1(phosphoSer759)

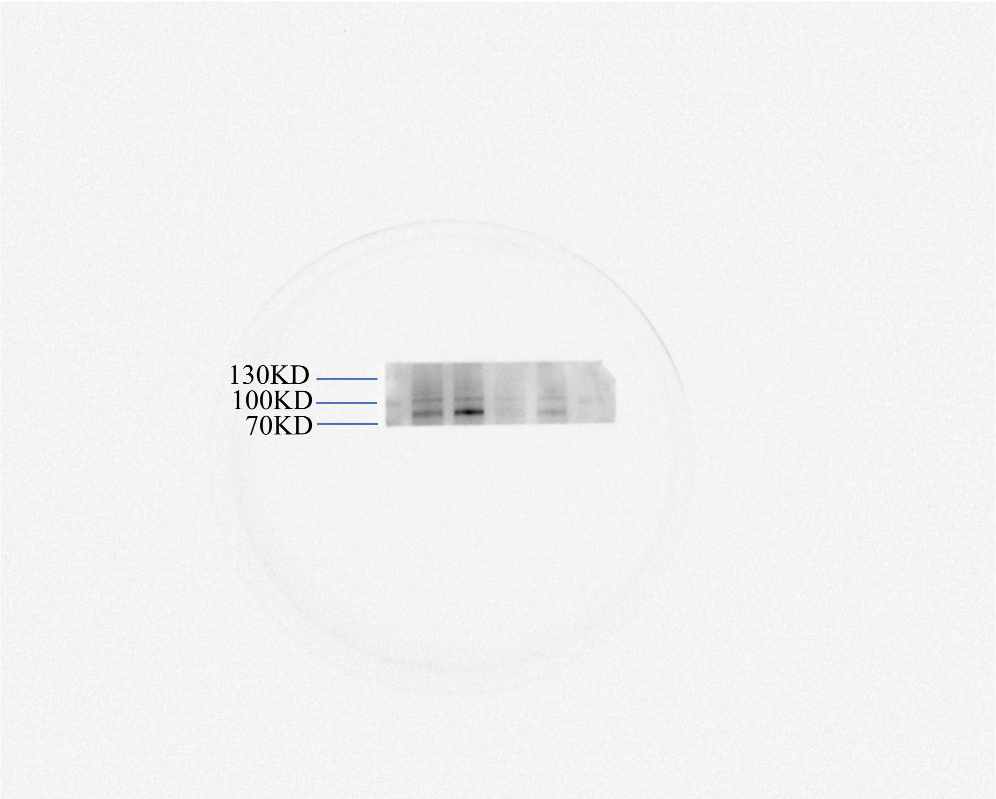

Figure6H HCCLM3 CALD1

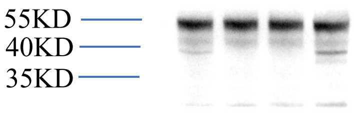

Figure6H HCCLM3 β-tubulin
Figure6H HCCLM3 AHSA1

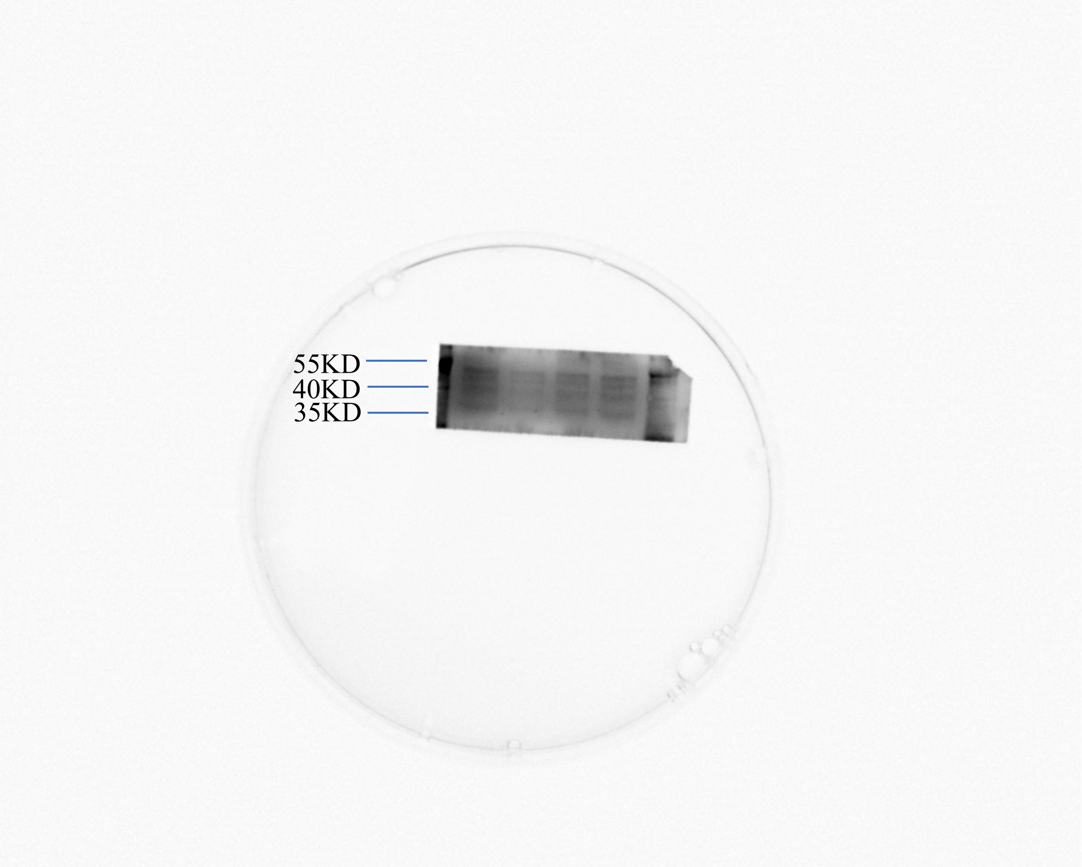

Figure6H HCCLM3 vimentin

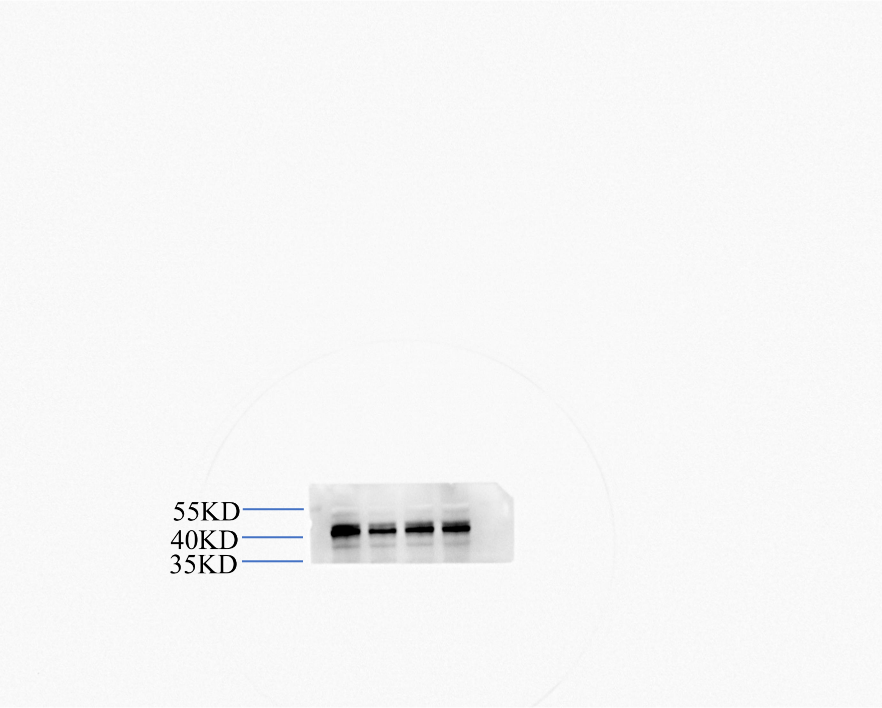

Figure6H HCCLM3 ERK1/2(phosphoThr202/Tyr204)

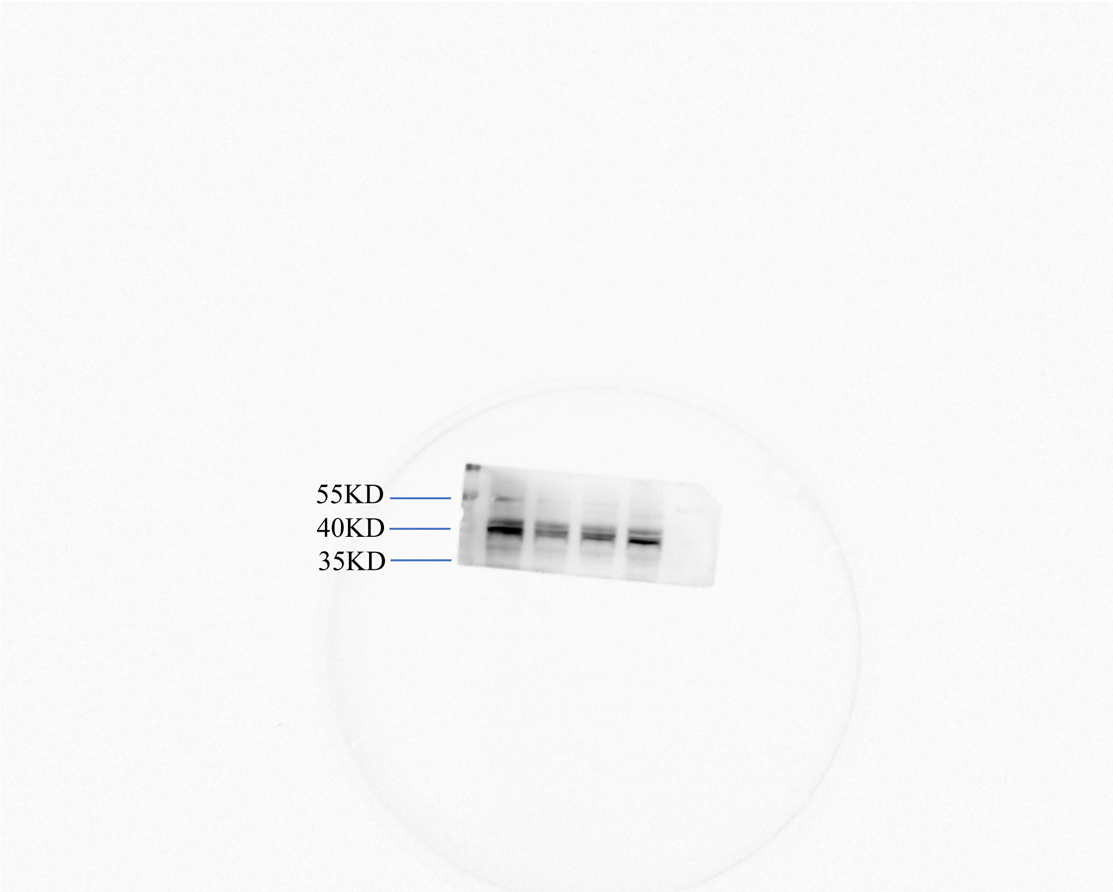

Figure6H HCCLM3 ERK1/2

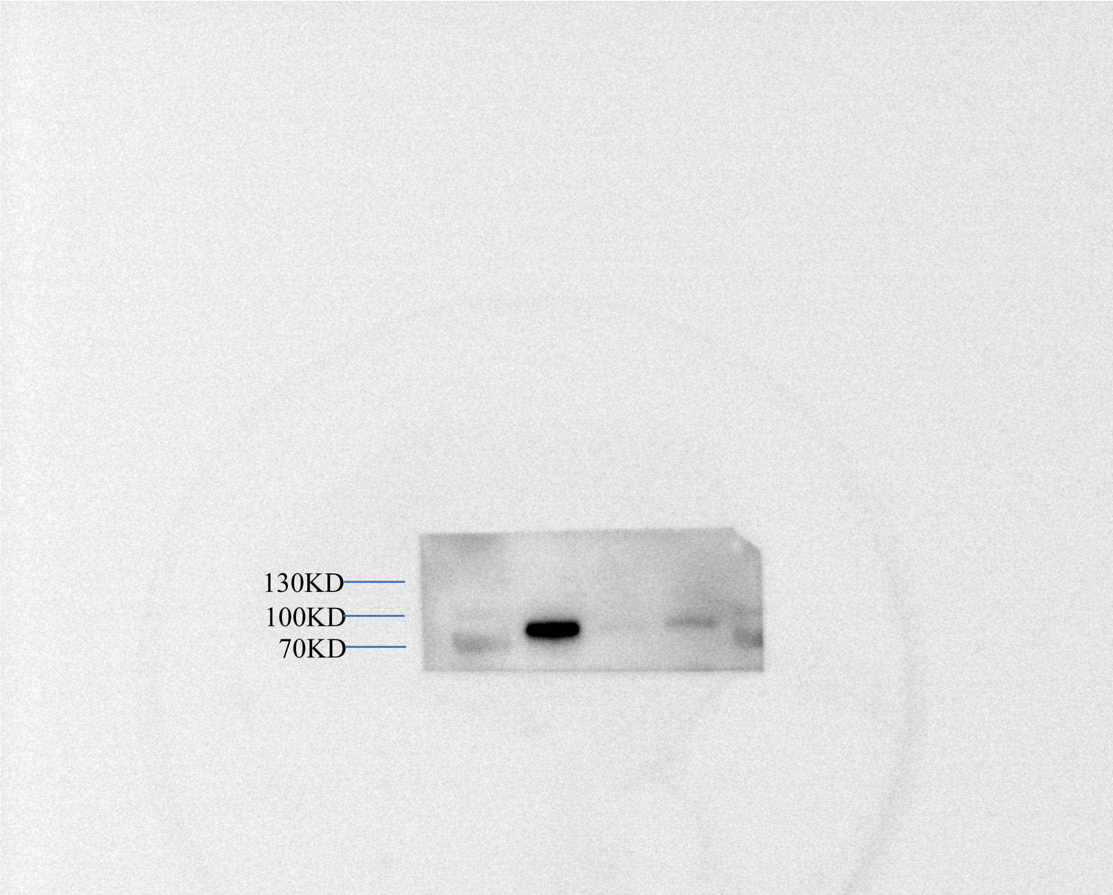

FigureS3A Huh-7 IP-AHSA1 CALD1

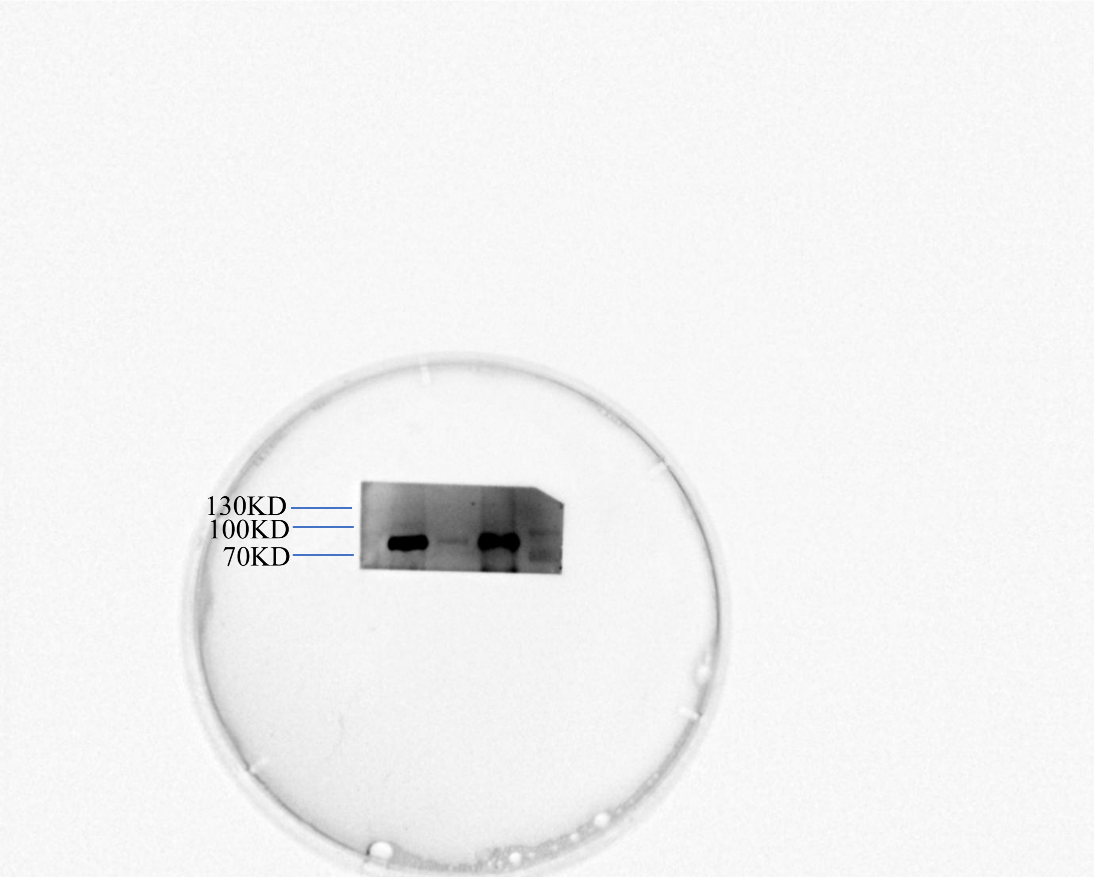

FigureS3A Huh-7 IP-CALD1 CALD1

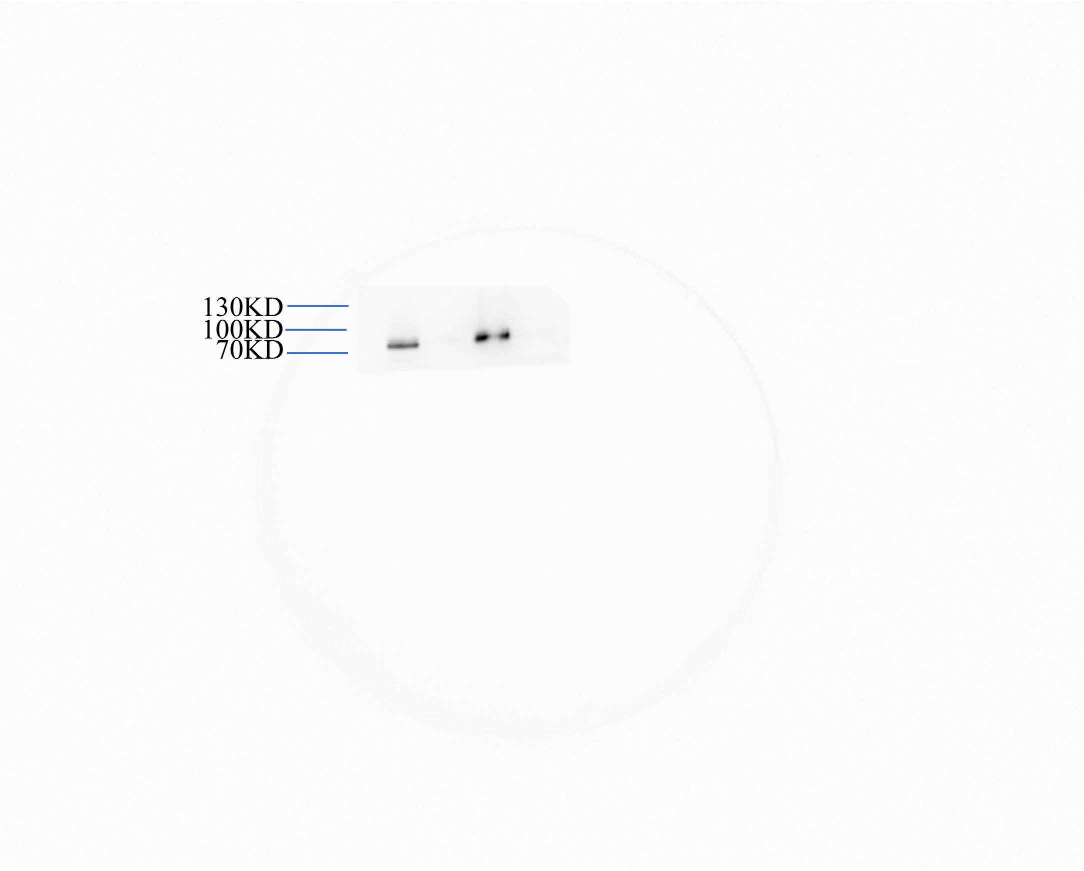

FigureS3A Huh-7 IP-ERK1/2 CALD1

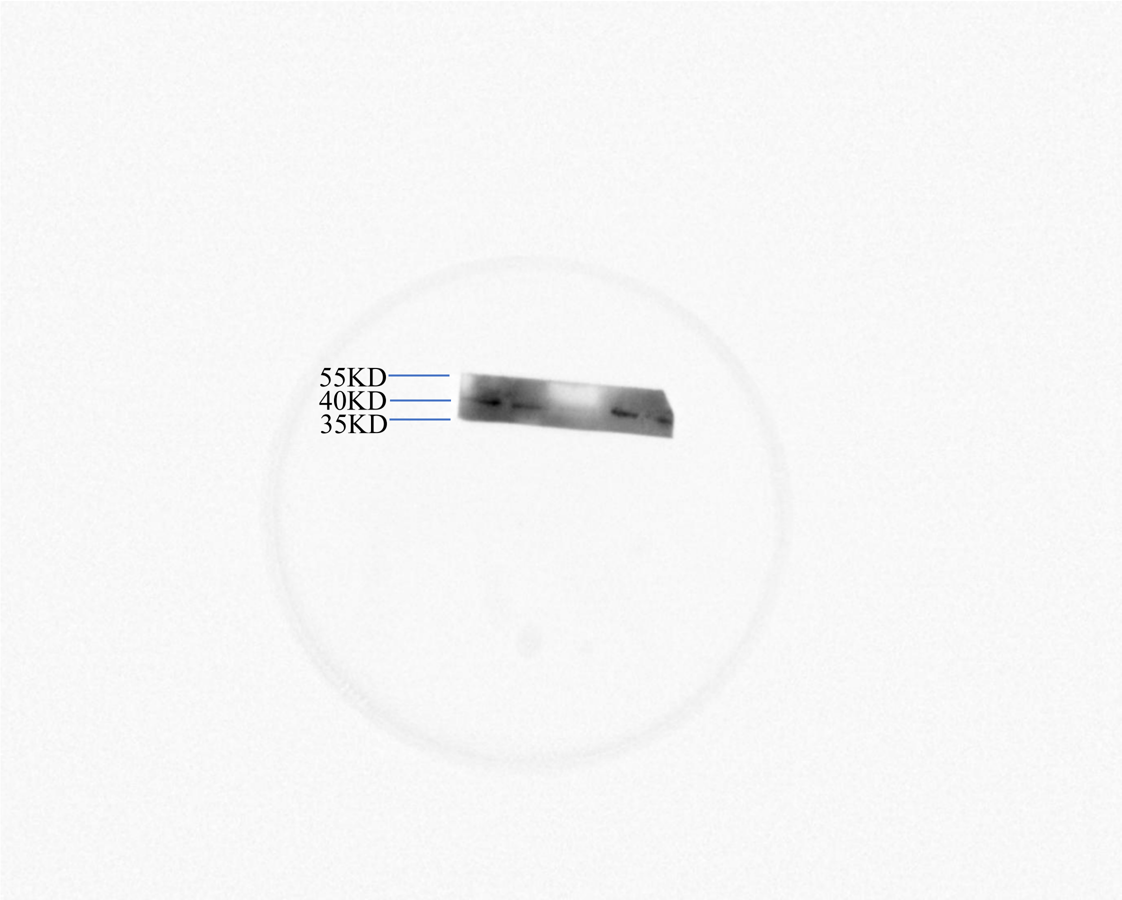

FigureS3A Huh-7 IP-AHSA1 ERK1/2

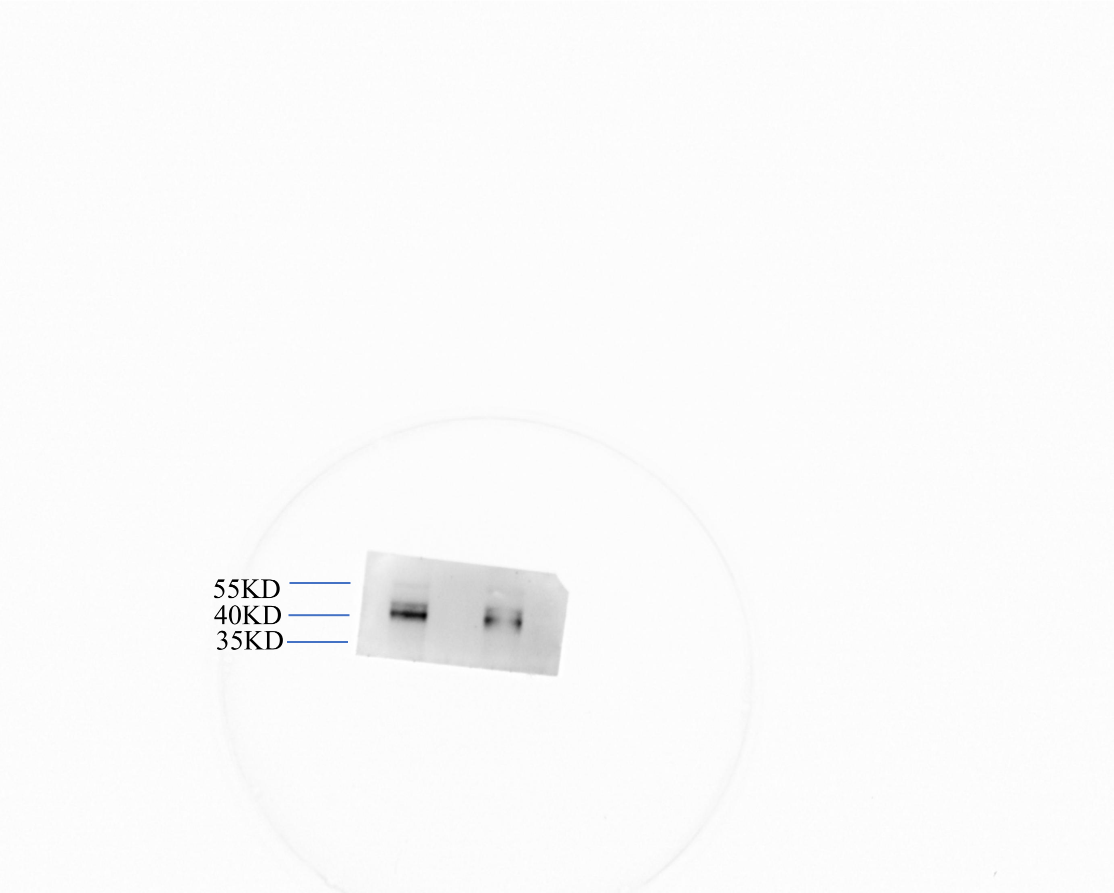

FigureS3A Huh-7 IP-CALD1 ERK1/2

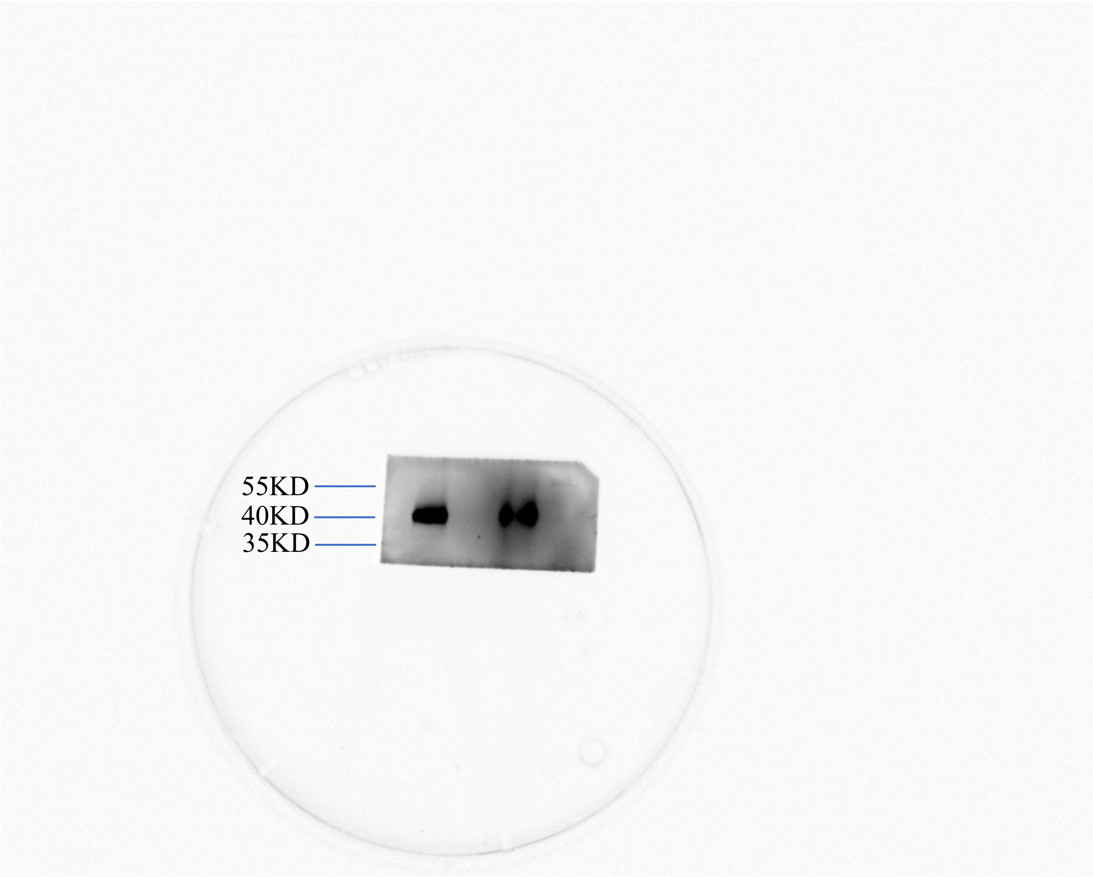

FigureS3A Huh-7 IP-ERK1/2 ERK1/2

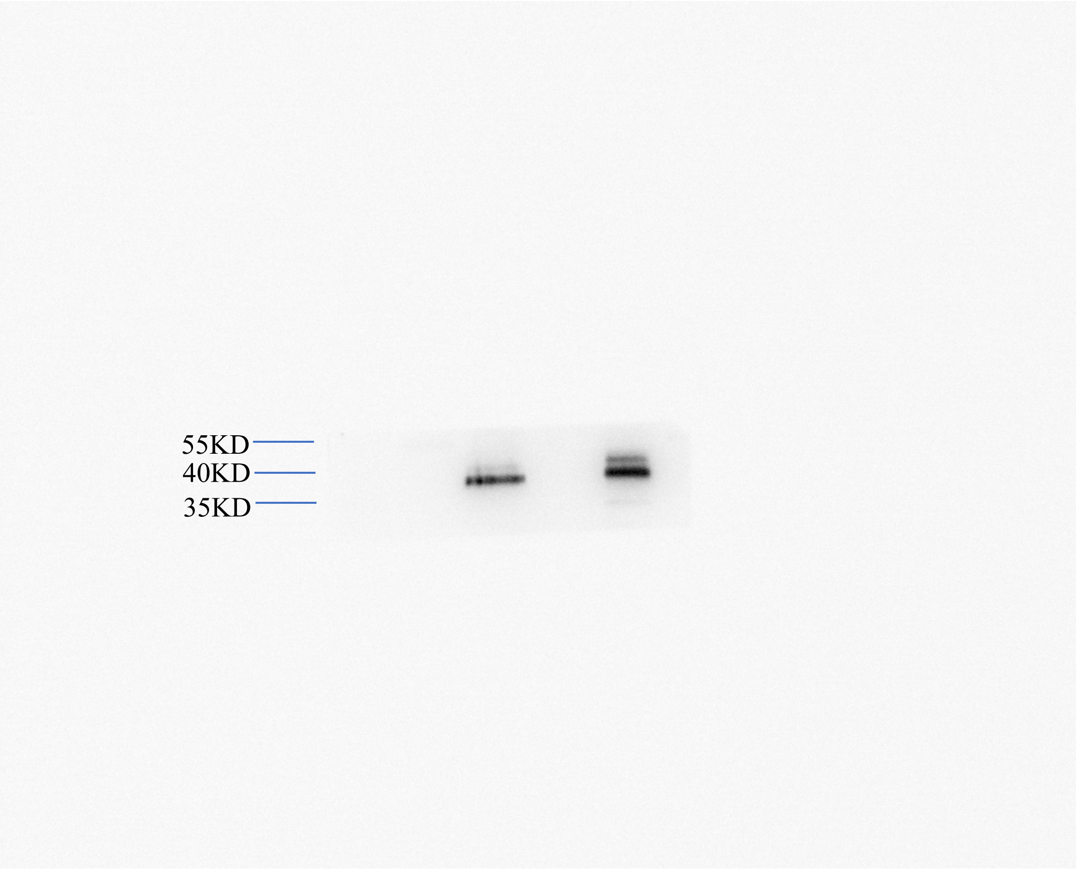

FigureS3A Huh-7 IP-AHSA1 AHSA1

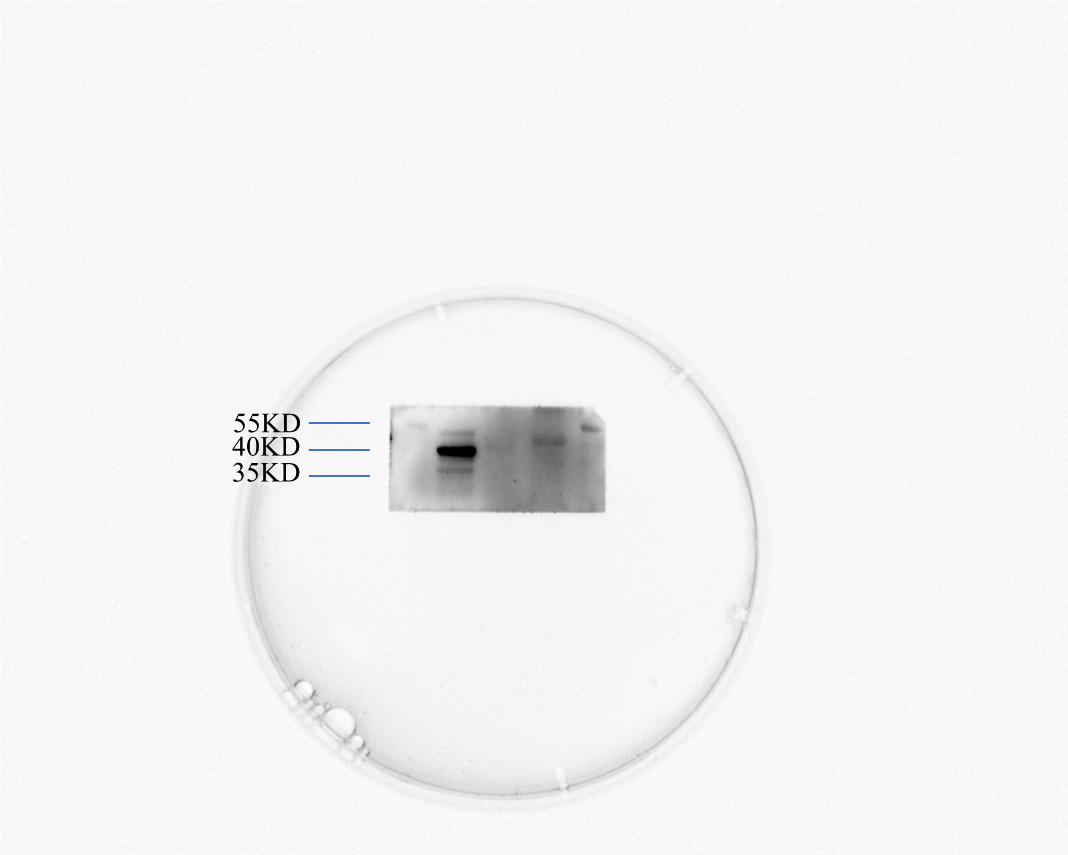

FigureS3A Huh-7 IP-CALD1 AHSA1

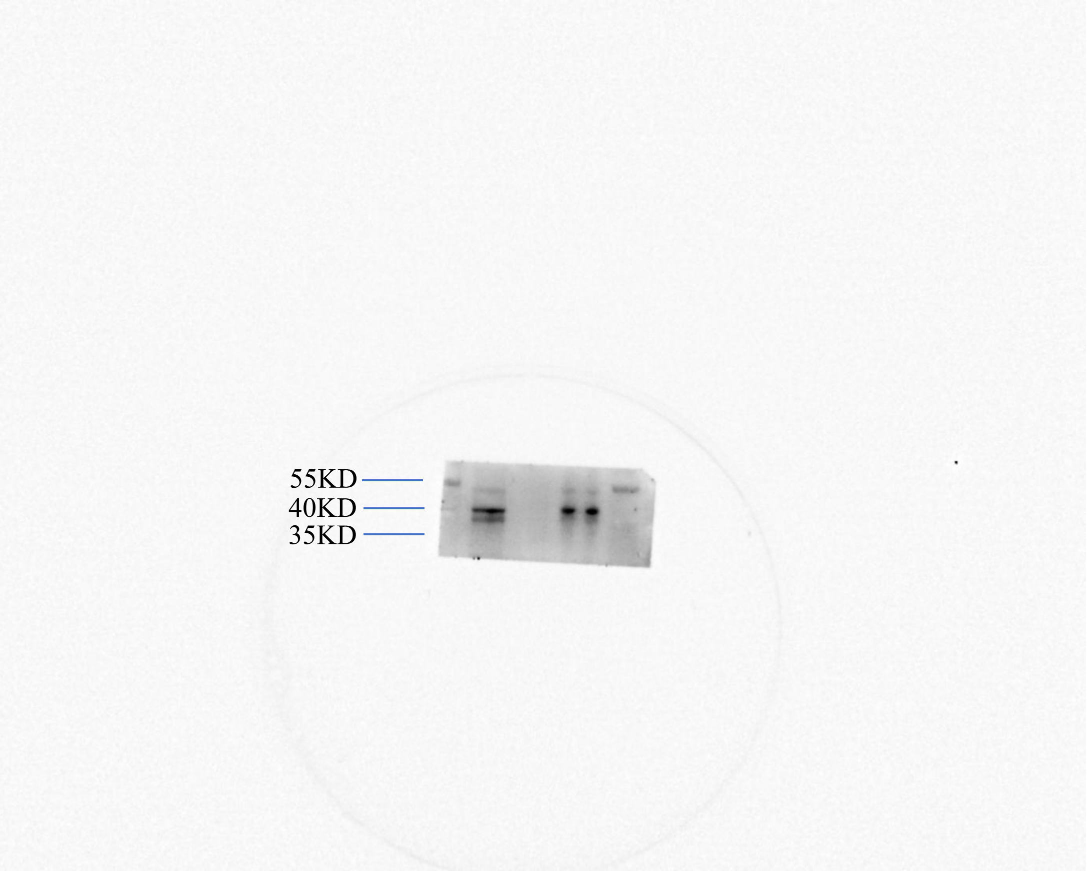

FigureS3A Huh-7 IP-ERK1/2 AHSA1

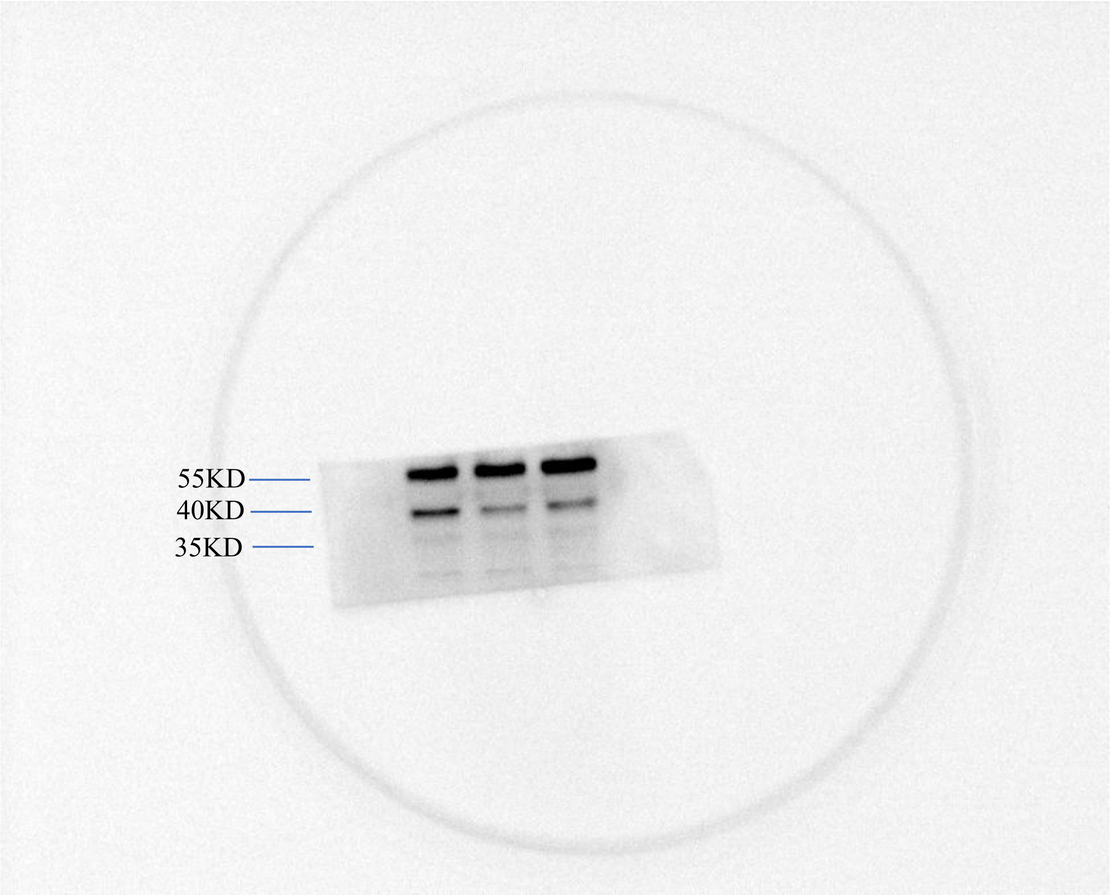

FigureS3C HCCLM3 β-tubulin
FigureS3C HCCLM3 AHSA1

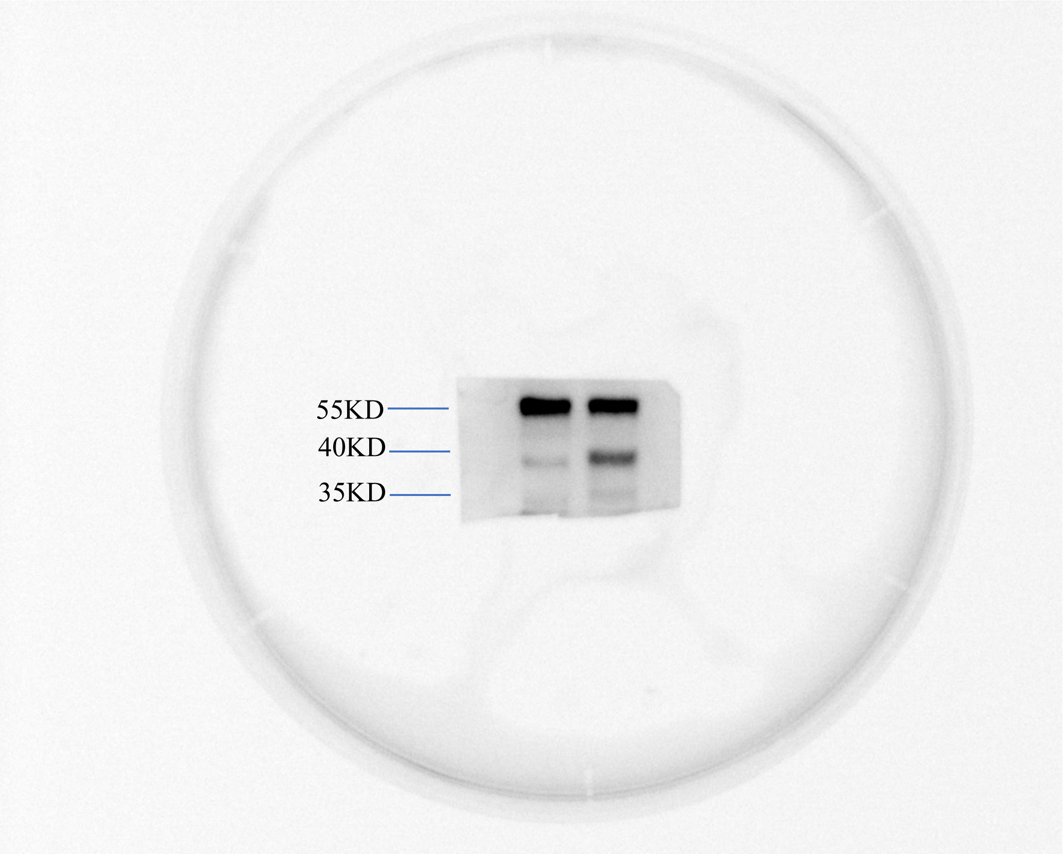

FigureS3C Hep3B β-tubulin
FigureS3C Hep3B AHSA1

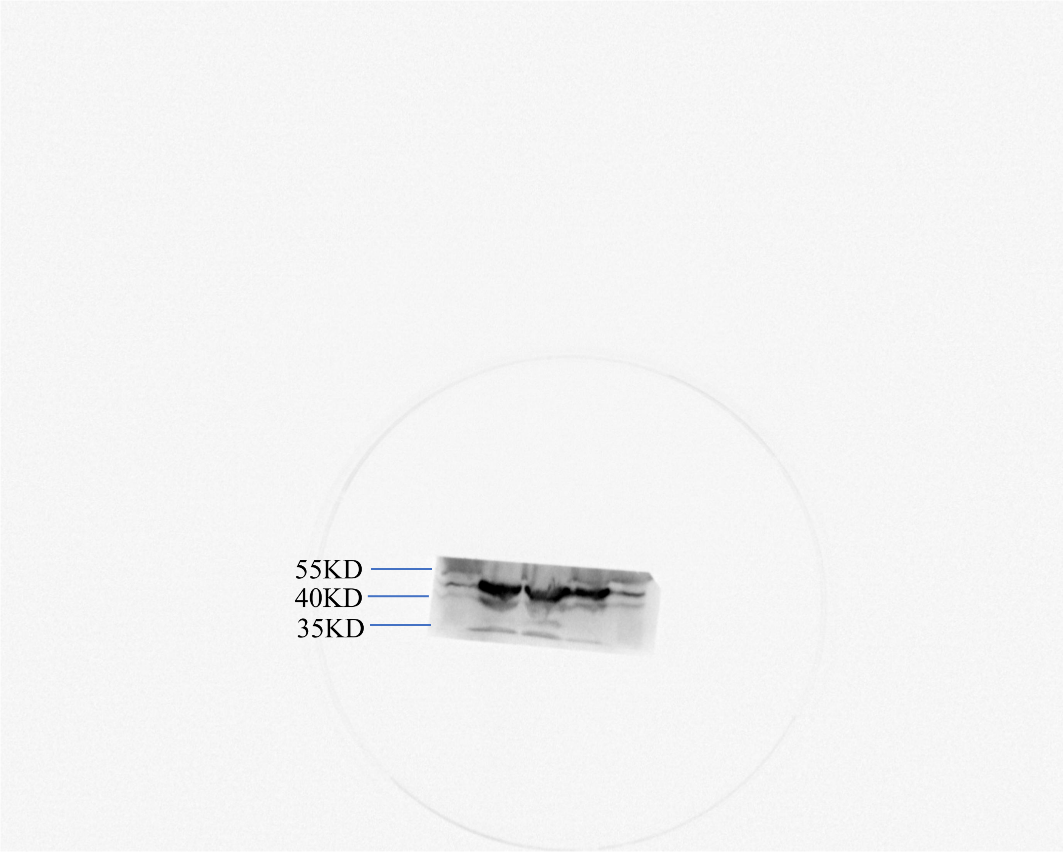

FigureS3C HCCLM3 MEK

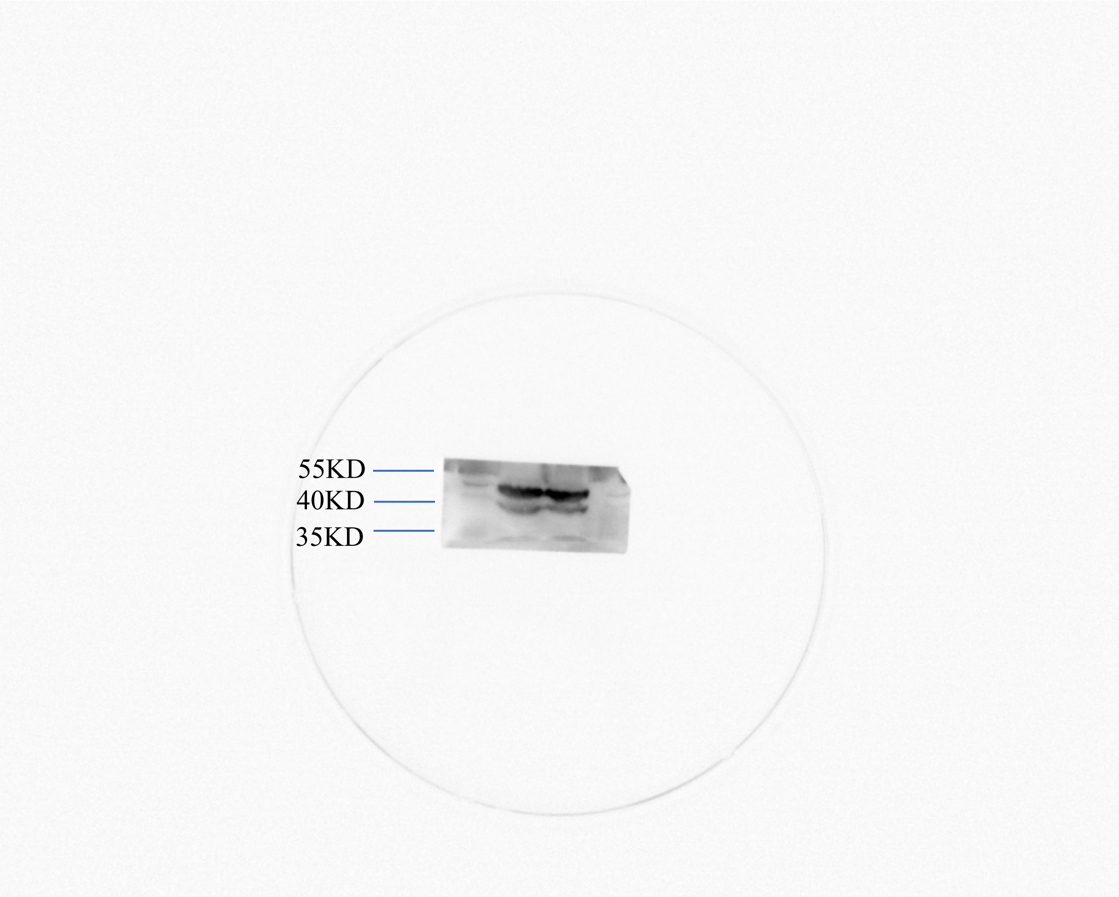

FigureS3C Hep3B MEK

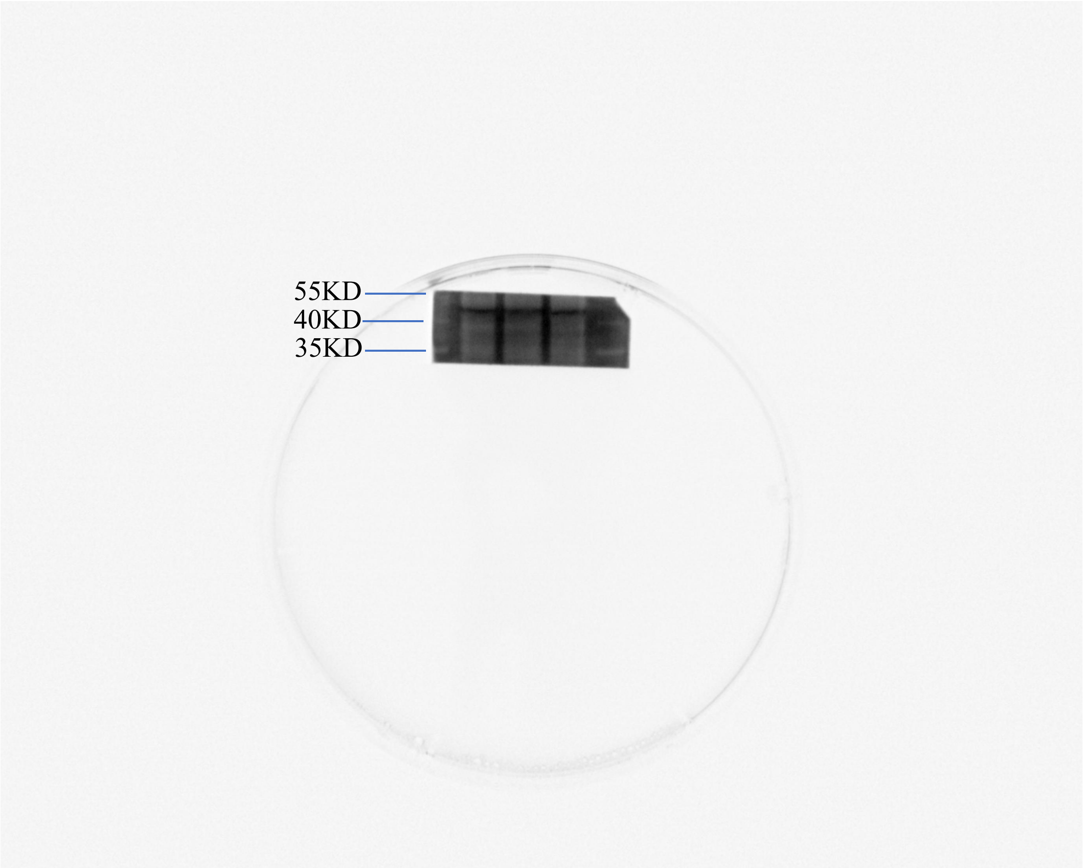

FigureS3C HCCLM3 MEK(phosphoSer217/221)

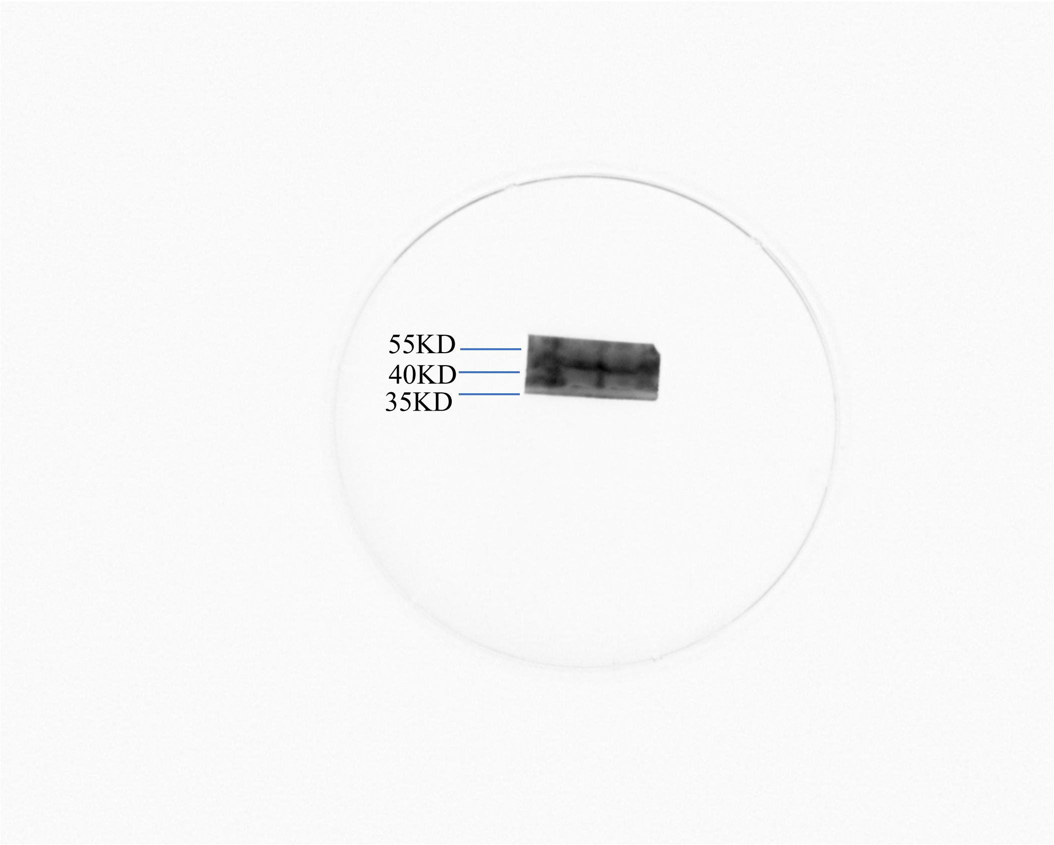

FigureS3C Hep3B MEK(phosphoSer217/221)

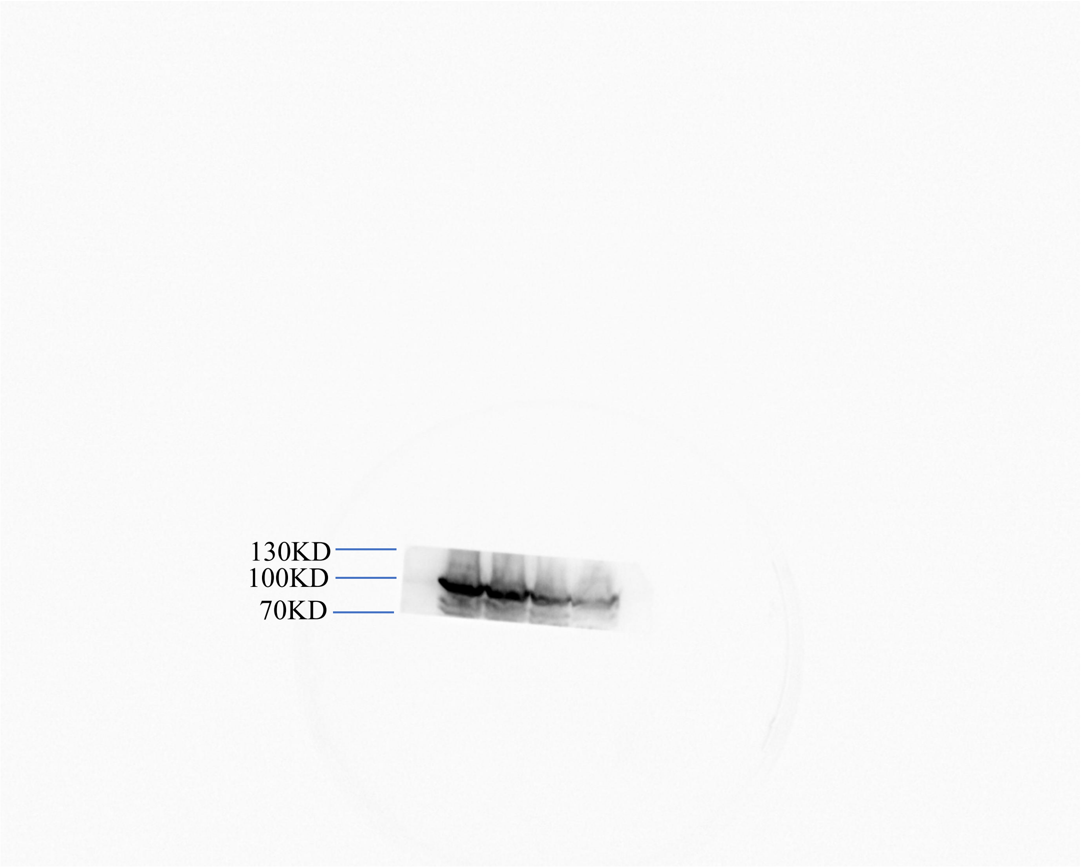

FigureS3D Hep3B HSP90

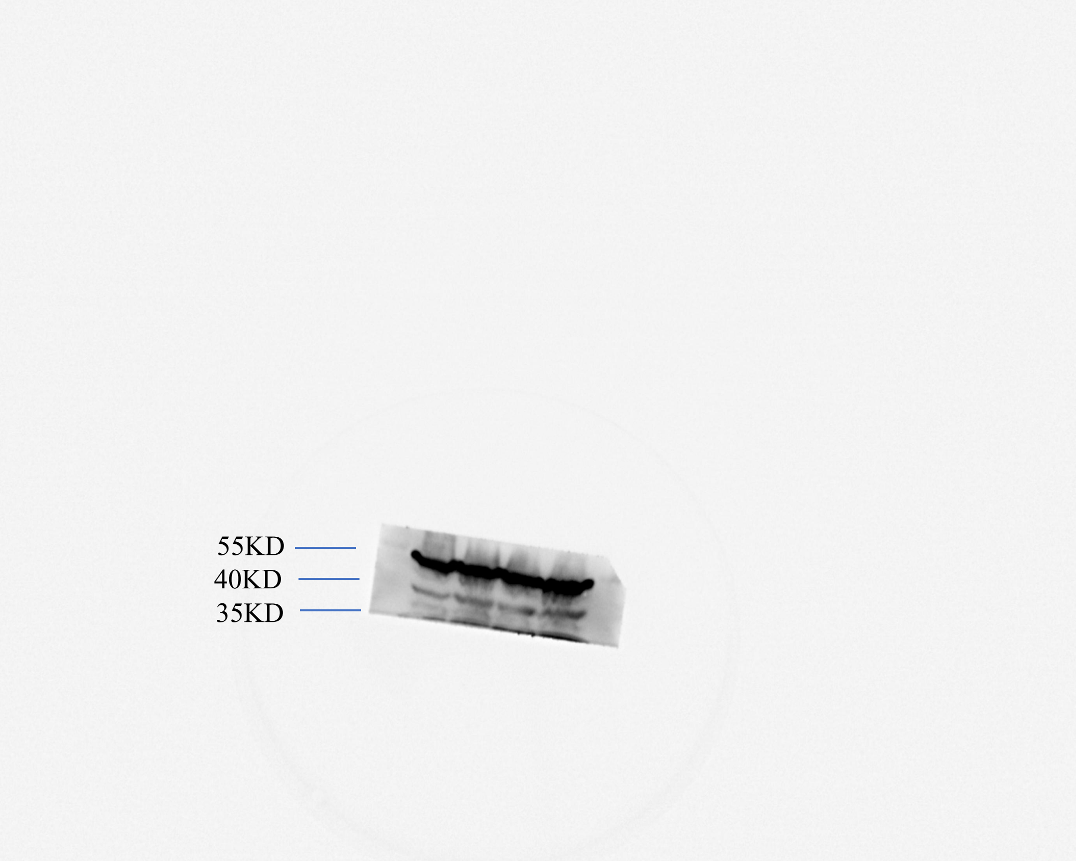

FigureS3D Hep3B β-tubulin

FigureS3D Hep3B AHSA1

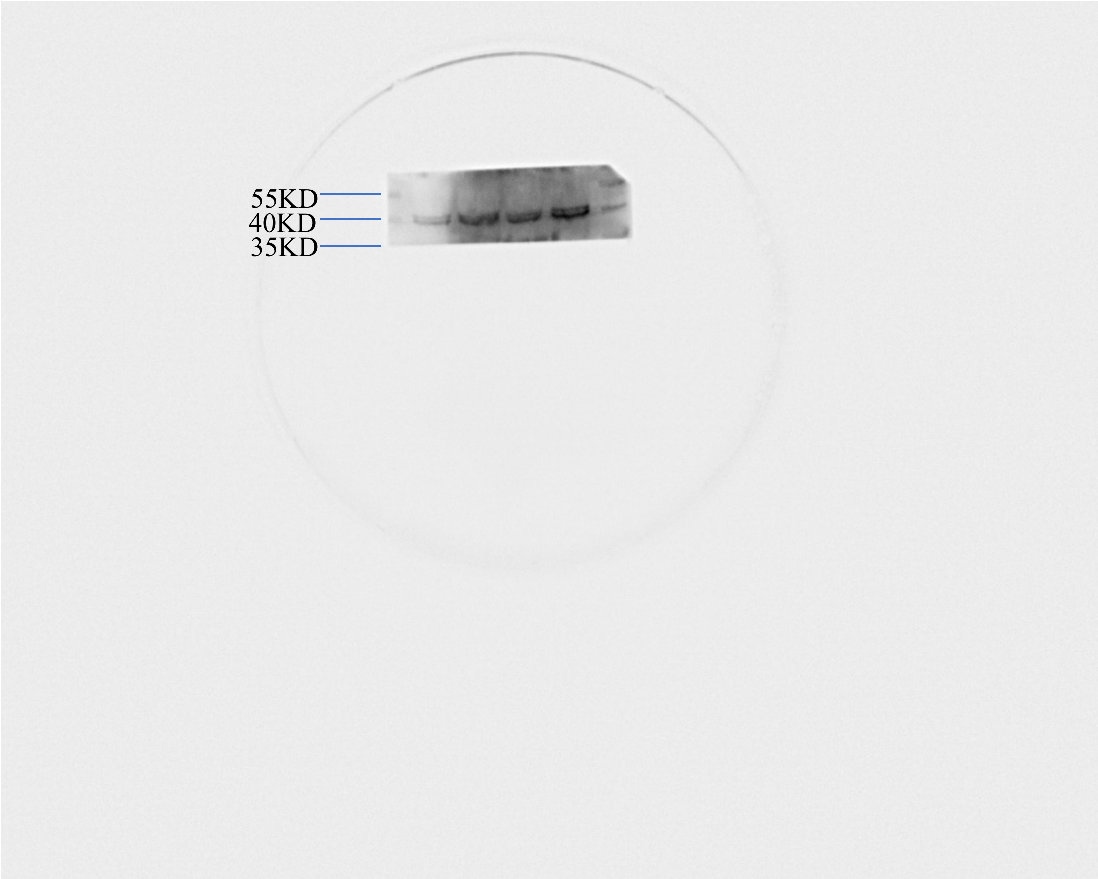

FigureS3D Hep3B ERK1/2(phosphoThr202/Tyr204)

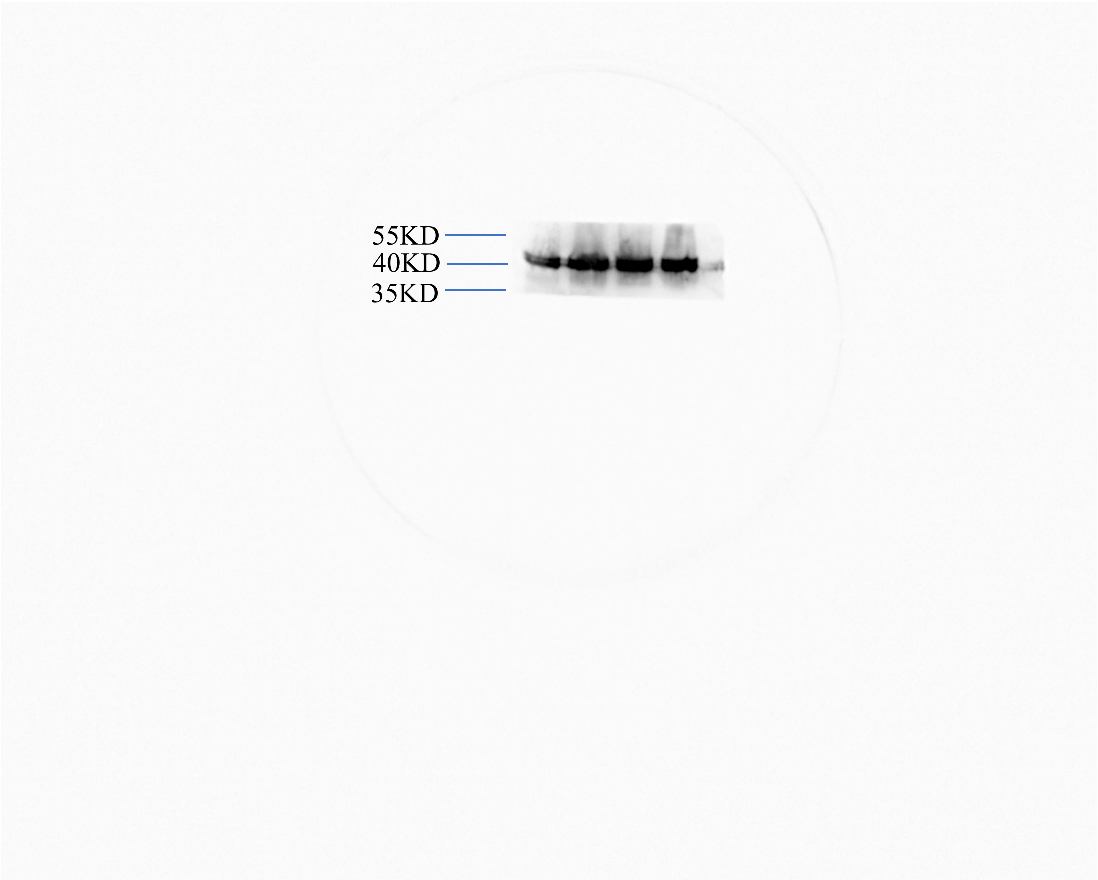

FigureS3D Hep3B ERK1/2
